# Genome-wide conserved non-coding microsatellite (CNMS) marker-based integrative genetical genomics for quantitative dissection of seed weight in chickpea

**DOI:** 10.1093/jxb/eru478

**Published:** 2014-12-10

**Authors:** Deepak Bajaj, Maneesha S. Saxena, Alice Kujur, Shouvik Das, Saurabh Badoni, Shailesh Tripathi, Hari D. Upadhyaya, C. L. L. Gowda, Shivali Sharma, Sube Singh, Akhilesh K. Tyagi, Swarup K. Parida

**Affiliations:** ^1^National Institute of Plant Genome Research (NIPGR), Aruna Asaf Ali Marg, New Delhi 110067, India; ^2^Division of Genetics, Indian Agricultural Research Institute (IARI), New Delhi 110012,India; ^3^International Crops Research Institute for the Semi-Arid Tropics (ICRISAT), Patancheru 502324, Telangana, India

**Keywords:** Chickpea, *Cicer arietinum*, CNMS, eQTLs, microsatellites, seed weight, SNPs, transcription factor.

## Abstract

Phylogenetic footprinting identified 666 genome-wide paralogous and orthologous CNMS (conserved non-coding microsatellite) markers from 5′-untranslated and regulatory regions (URRs) of 603 protein-coding chickpea genes. The (CT)_n_ and (GA)_n_ CNMS carrying CTRMCAMV35S and GAGA8BKN3 regulatory elements, respectively, are abundant in the chickpea genome. The mapped genic CNMS markers with robust amplification efficiencies (94.7%) detected higher intraspecific polymorphic potential (37.6%) among genotypes, implying their immense utility in chickpea breeding and genetic analyses. Seventeen differentially expressed CNMS marker-associated genes showing strong preferential and seed tissue/developmental stage-specific expression in contrasting genotypes were selected to narrow down the gene targets underlying seed weight quantitative trait loci (QTLs)/eQTLs (expression QTLs) through integrative genetical genomics. The integration of transcript profiling with seed weight QTL/eQTL mapping, molecular haplotyping, and association analyses identified potential molecular tags (GAGA8BKN3 and RAV1AAT regulatory elements and alleles/haplotypes) in the LOB-domain-containing protein- and KANADI protein-encoding transcription factor genes controlling the *cis*-regulated expression for seed weight in the chickpea. This emphasizes the potential of CNMS marker-based integrative genetical genomics for the quantitative genetic dissection of complex seed weight in chickpea.

## Highlight

Development and an integrated utilization of genome-wide conserved non-coding microsatellite (CNMS) markers in genetical genomics for quantitative dissection of seed weight in chickpea are described.

## Introduction

The utility of non-coding microsatellites, specifically in the 5′-untranslated and regulatory regions (URRs) of genes has been widely documented in diverse plant and mammalian genomes for various marker-based large-scale genotyping applications (Hulzink *et al.*, 2002; Sangwan and O’Brian, 2002; Fujimori *et al.*, 2003; Iglesias *et al.*, 2004; Martin *et al.*, 2004; Meister *et al.*, 2004; Kooiker *et al.*, 2005; Lawson and Zhang, 2006; Zhang *et al.*, 2006; Parida *et al.*, 2009). The alterations of such microsatellite have implicated them in controlling transcription/translation functions. A number of studies in crop plants have already demonstrated the association of altered non-coding regulatory microsatellites in specific genes with traits of agronomic importance. This is clearly evident from the correlation of the varied lengths of (CT)_n_/(GA)_n_ microsatellite repeats in the 5ʹ-UTR (untranslated region) of the *waxy* gene with amylose content in rice (Bao *et al.*, 2002). Further, alterations of non-coding microsatellite repeats, such as (CT)_n_/(GA)_n_ and (CTT)_n_/(GAA)_n_, in the *cis-*regulatory element-binding regions (TCA-element and GAGA-binding factor) of genes controlling light and salicylic acid responses, respectively, in *Arabidopsis* and *Brassica* have been reported (Zhang *et al.*, 2006). Early intra-/intergenomic phylogenetic footprinting studies that specifically investigated monocot and dicot plant genomes have indicated the presence of non-coding microsatellites in the functionally constrained regulatory sequence element regions and association of such microsatellite repeat motifs with evolutionarily conserved non-coding sequences (CNS) (Tagle *et al.*, 1988; Levy *et al.*, 2001; Koch *et al*., 2000, 2001; Colinas *et al.*, 2002; Morishige *et al.*, 2002; Guo and Moose, 2003; Santi *et al.*, 2003; Creux *et al.*, 2008; Spensley *et al.*, 2009; Freeling and Subramaniam, 2009; Baxter *et al.*, 2012). This suggests their utility as conserved non-coding microsatellite (CNMS) markers in defining gene regulatory functions and understanding non-coding regulatory sequence evolution in many crop plants (Zhang *et al.*, 2006; Parida *et al.*, 2009). Considering the inherent genetic attributes of CNMS markers, the development of such informative markers by targeting the microsatellite repeat motifs in the known regulatory elements/functional transcription factor-binding sites (TFBS) from the non-coding upstream sequence components of protein-coding genes would be of practical significance. They could thus be effectively utilized for the rapid detection of marker–trait linkages and the dissection of qualitative and complex quantitative traits, such as yield and/or stress tolerance in the chickpea.

With the availability of structurally and functionally annotated protein-coding genes (27 571–28 269) from the recently released draft genome sequences of the *desi* and *kabuli* chickpea genotypes (Jain *et al.*, 2013; Varshney *et al.*, 2013), it is now possible to mine diverse known regulatory elements/TFBS (such as GAGA8HVBKN3, RAV1AAT, CTRMCAMV35S, ANAERO2CONSENSUS, and GATABOX) from the URRs using available databases (~500–1000 characterized regulatory elements/TFBS), including PLACE, PlantCARE, and TRANSFAC (http://www.dna.affrc.go.jp/PLACE, http://bioinformatics.psb.ugent.be/webtools/plantcare/html; Wingender *et al.*, 2000) in crop plants. Consequently, microsatellite repeat motifs from these regulatory elements/TFBS identified in genes can be targeted to develop large-scale genome-wide *in silico* CNMS markers. Currently, an integrated approach of gene expression profiling and genetic/quantitative trait loci (QTL) mapping called ‘genetical genomics/expression genetics’ (Jansen and Nap, 2001; Gupta and Rustgi, 2004) has successfully delineated trait-specific functionally relevant gene targets rapidly from a larger set of differentially expressed known/candidate genes controlling various agronomic traits (yield and stress tolerance) in many crop plants. Alternatively, this approach is recognized as ‘eQTL (expression QTL) mapping’ in which the measured transcript levels in individuals that have undergone advanced generation mapping has been utilized to map the polymorphic genomic loci harbouring the QTLs that alter the expression/accumulation of specific transcripts (Schadt *et al.*, 2003; Gibson and Weir, 2005). eQTL mapping largely helps to identify ‘*cis*’ and ‘*trans*’ eQTLs based on the linkage of polymorphic genomic loci that have been identified in the eQTLs with the physical positions of the measured gene-encoding transcripts. Moreover, this integrative approach is useful for dissecting the molecular basis of most quantitative traits and deciphering the complex gene/QTL regulatory networks controlling expression polymorphisms that underlie both simple and complex traits, including phosphate sensing, flowering time, and growth/development in crop plants (Johanson *et al.*, 2000; Schadt *et al.*, 2003; Werner *et al.*, 2005; Clark *et al.*, 2006; Potokina *et al.*, 2006; Caicedo *et al.*, 2007; Salvi *et al.*, 2007; Svistoonoff *et al.*, 2007; Emilsson *et al.*, 2008; Boerjan and Vuylsteke, 2009; Kim *et al.*, 2010; Kloosterman *et al.*, 2010; Terpstra *et al.*, 2010). Thus, the CNMS markers that regulate gene expression on the basis of variation in the microsatellite repeat length in the known functional regulatory elements/TFBS of these genes may be of great relevance for the targeted mapping of differentially expressed genes in the chickpea genome by combining transcript profiling in segregating mapping individuals with genetic/QTL mapping studies. This information would expedite the identification and mapping of genes in addition to eQTLs and their regulatory sequences that are specifically involved in the expression of complex quantitative agronomic traits. Moreover, the identification of gene-based CNMS markers on a genome-wide scale will serve as a cost-effective approach to facilitate the more widespread use of genetical genomics/eQTL mapping for the multifactorial dissection of the gene/QTL expression profile levels governing various genetic components of complex agronomic traits in the chickpea.

Therefore, the present study was undertaken to identify and characterize the CNMS repeat motifs from the 5ʹURRs of protein-coding chickpea genes on a genome-wide scale. The potential of these validated CNMS markers was evaluated for various applications, including the construction of physical and functional transcript maps and the elucidation of gene regulatory functions. The utility of these markers was further assessed to identify and map the novel potential regulatory elements and alleles in the candidate genes controlling the complex quantitative trait of 100-seed weight in the chickpea using an integrative genetical genomics approach (by combining differential expression profiling with QTL and eQTL mapping, selective genotyping, molecular haplotyping, and genetic association analyses) in natural and mapping populations.

## Materials and methods

### Discovery, characterization, and physical mapping of genic CNMS markers in chickpea

The transcript FASTA sequences of two chickpea genotypes, ICCV2 (*kabuli*; 43389 transcripts, Agarwal *et al.*, 2012) and PI489777 (wild *Cicer reticulatum*; 37 265, Jhanwar *et al.*, 2012), and 14 486 unigenes were retrieved from the Chickpea Transcriptome Database (CTDB) version 1.0 (http://www.nipgr.res.in/ctdb.html). The structural annotations for different coding and non-coding URR sequence components of these transcripts were performed using the NCBI ORF Finder (http://www.ncbi.nlm.nih.gov/projects/gorf), FGNESH (www.softberry.com), ORF Finder (http://bioinformatics.org/sms/orf_find.html), and UTRScan (http://itbtools.ba.itb.cnr.it/utrscan), respectively. Additionally, 1000bp FASTA sequences localized upstream from the annotated translation start site/initiation codon (ATG) of 28 269 and 27 571 protein-coding genes as predicted from the draft genome sequences of the chickpea *kabuli* (CDC Frontier; Varshney *et al.*, 2013) and *desi* (ICC 4958; Jain *et al.*, 2013) genotypes, respectively, were batch downloaded. Likewise, 1000-bp upstream FASTA sequences were also acquired of 230 161 genes annotated from four legumes (*Medicago truncatula*, *Lotus japonicus*, *Cajanus cajan*, and *Glycine max*) and two non-legume sequenced dicot species (*Arabidopsis thaliana* and *Vitis venifera*) (Singh *et al.*, 2012; Varshney *et al.*, 2012; http://www.plantgdb.org; http://www.arabidopsis.org). The sequences located upstream from the initiation codons of protein-coding genes for all seven dicot species, including the chickpea, were acquired taking into account their structural (orientation) annotation information across the chromosomal pseudomolecules of the sequenced genomes.

**Fig. 1.**
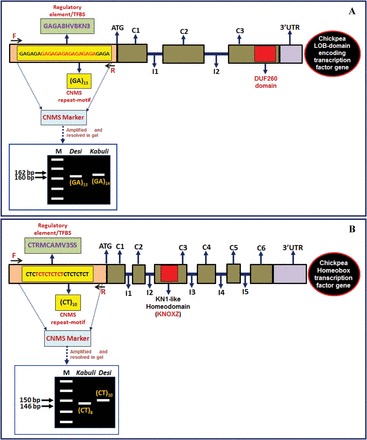
Development of CNMS markers from the known regulatory elements/TFBS present in the sequences upstream of the translation initiation codons (ATG) of protein-coding chickpea genes. The forward and reverse primers designed from the sequences flanking the (GA)_n_ and (CT)_n_ CNMS repeat motifs carrying known GAGA8HVBKN3 and CTRMCAMV35S regulatory elements/TFBS of LOB**-** (A) and homeobox (B) domain protein-encoding TF genes, respectively, were used to develop CNMS markers in chickpea. (This figure is available in colour at *JXB* online.)

To reveal potential and robust CNS in the chickpea on a genome-wide scale, upstream gene sequences obtained from seven plant species were analysed using the alignment plot/dot plot method (seaweed algorithm; Krusche and Tiskin, 2010) of APPLES (http://www2.warwick.ac.uk/fac/sci/systemsbiology/staff/ott/tools_and_software/apples) as described by Baxter *et al.* (2012) and Reineke *et al.* (2011). The CNS identified in the chickpea genes were further correlated/compared with 1865 previously documented CNS from 1643 *Arabidopsis* genes and 602 CNS from 554 orthologous genes of four dicot plants (*A. thaliana*, *Carica papaya*, *Populus trichocarpa*, and *V. vinifera*; Baxter *et al.*, 2012). To derive the over-representation of the CNS for known regulatory elements/TFBS, the CNS from chickpea gene sequences were analysed with the intra-/intergenomic phylogenetic footprinting method of rVISTA (Loots *et al.*, 2002; http://genome.lbl.gov/vista/rvista/submit.shtml) with default parameters using known TFBS databases, such as TRANSFAC (Wingender *et al.*, 2000), PLACE (http://www.dna.affrc.go.jp/PLACE/), and PlantCARE (http://bioinformatics.psb.ugent.be/webtools/plantcare/html/) as references. The CNS identified in the chickpea genes were mined for microsatellites using MISA (Microsatellite; http://pgrc.ipk-gatersleben.de/misa). Those in which the localization of microsatellite repeat motifs corresponded to the positions of known regulatory elements/TFBS that are conserved in at least one of the orthologous/paralogous genes of the seven dicot species studied were defined as ‘conserved non-coding microsatellites (CNMS)’. The unique forward and reverse primers with amplification product sizes of 100–300bp were designed from the sequences flanking these CNMS repeat motifs carrying regulatory elements using BatchPrimer3 (http://probes.pw.usda.gov/batchprimer3) and developed as gene-based CNMS markers (Fig. 1). The frequency distributions of the CNMS markers were determined on the basis of their localizations in average 100bp sequence intervals upstream from the initiation codons of chickpea genes. The CNMS markers in the genes were functionally annotated based on their correlations with known/predicted functional characteristics of TFBS/regulatory elements with which the microsatellite repeat motifs co-localized, and putative functions of the corresponding CNMS marker-associated genes were assigned. A gene ontology (GO) enrichment analysis (biological process, molecular function, and cellular component) of CNMS marker-associated genes was performed using the BiNGO plugin of Cytoscape V2.6 (Maere *et al.*, 2005). The Benjamini and Hochberg false discovery rate correction was implemented for the GO enrichment significance test (*P*≤0.05). To ascertain the physical positions (bp) of the designed genic CNMS markers across the chromosomes, the non-coding upstream gene regulatory sequences flanking the CNMS repeat motifs were analysed according to the methods of Kujur *et al.* (2013).

### Validation of CNMS markers

To access the amplification and polymorphic potential of the *in silico* designed genic CNMS markers, a total of 666 markers were PCR amplified using the genomic DNA of 24 *desi* and *kabuli* genotypes and one wild (*C. reticulatum*) genotype (Supplementary Table S1 available at *JXB* online) and resolved through a gel-based assay and fluorescent dye-labelled automated fragment analyser following the methods of Kujur *et al.* (2013). The validated polymorphic CNMS markers distributed over the eight chickpea chromosomes were used to estimate their amplified allele numbers, percentage polymorphism, and polymorphism information content (PIC) using PowerMarker version 3.51 (Liu and Muse, 2005).

### Evaluation of CNMS marker-associated genes for differential expression

A set of 256 informative CNMS marker-associated genes showing polymorphism among 25 chickpea genotypes were selected for the differential expression profiling. The total RNA isolated (Trizol; Invitrogen, USA) from the vegetative (root, shoot and leaf) and reproductive (flower bud and young pod) tissues and two different seed developmental stages [early cell division phase occurring 10–20 days after podding (DAP) and late maturation phase occurring 21–30 DAP] of three contrasting low and high/very high seed weight *desi* and *kabuli* genotypes, including ICCX-810800 (100-seed weight 11g), ICC 4958 (35g), and ICC 20268 (47g). These samples were used in the semi-quantitative and quantitative real-time PCR (RT-PCR) assays according to Kujur *et al.* (2013). A housekeeping gene, elongation factor 1-alpha (*EF1α*), suitable for consistent expression across diverse tissues and developmental stages of chickpea genotypes (Garg *et al.*, 2010) was utilized as the internal control in the RT-PCR assay. In quantitative RT-PCR, three independent biological replicates of each sample and two technical replicates of each biological replicate with no template and primer as control were included. The expression values across various tissues and seed developmental stages of genotypes were normalized against endogenous control *EF1α*. Significant differences in gene expression at two seed developmental stages of low and high seed weight genotypes as compared with leaf (considered as the reference calibrator and assigned as 1) was performed by least significant differene (LSD)-analysis of variance (ANOVA) significance test using SPSS 17.0 (http://www.spss.com/statistics). The differential expression data generated by the CNMS marker-associated gene-specific primers were compared and correlated to construct a heat map using the TIGR MultiExperiment Viewer (MeV; http://www.tm4.org/mev.html). Based upon this, the CNMS marker-associated genes showing differential, in addition to preferential and tissue-specific, expression in the diverse tissues and seed developmental stages of genotypes were identified.

To infer the molecular basis of the differential CNMS marker-associated gene expression, fragment length polymorphism based on the expansion/contraction of the CNMS repeat units in the regulatory elements/TFBS of the genes were correlated with their differential expression profiles. For this, the cDNA and genomic DNA fragments amplifying the total coding and non-coding 5ʹ upstream sequence components (1000bp) of the differentially expressed genes in ICCX-810800, ICC 4958, and ICC 20268 were purified, cloned, and sequenced in both the forward and reverse directions twice on a capillary-based Automated DNA Sequencer (Applied Biosystems, ABI 3730xl DNA Analyzer, Vernon Hills, IL, USA). The high-quality consensus sequences obtained for each CNMS marker-associated gene were aligned and compared among the three genotypes. The presence of predicted CNMS repeat motifs in the regulatory elements/TFBS of the genes and the correspondence of fragment length polymorphism based on CNMS repeat unit expansion/contraction with the differential expression profiling of each gene were inferred among the chickpea genotypes.

### Transcript map construction and QTL mapping

To construct the genetic linkage (transcript) map, the genotyping data of a selected set of physically mapped gene-based CNMS markers showing polymorphism between two parental chickpea genotypes, ICC 4958 (100-seed weight 35g) and ICC 17163 (15g), and 190 segregating individuals of a F_7_ recombinant inbred line (RIL) mapping population (ICC 4958×ICC 17163), were analysed using the JoinMap 4.1 (http://www.kyazma.nl/index.php/mc.JoinMap) at an LOD (logarithm of odds) threshold of >4.0 with the Kosambi function. The CNMS markers in each gene were assigned to their defined linkage groups (LGs) in accordance with their physical positions (bp) and groupings shared on the corresponding chromosomes as determined by the physical mapping analysis. The genetic linkage (transcript) map was constructed according to the centiMorgan (cM) map distances of the genic CNMS markers integrated on the chickpea LGs/chromosomes.

For the genetic/QTL mapping, 190 RIL mapping individuals (ICC 4958×ICC 17163) along with their parental genotypes were phenotyped in the field across two geographical locations in India with at least three replicates (following randomized complete block design) for three consecutive years. The 100-seed weight (g) was measured by estimating the average weight of 100 seeds with 10% moisture content. This was performed by selecting 10–12 representative plants from each RIL in addition to the parents at maturity. The frequency distribution, range, and ANOVA of 100-seed weight-specific phenotypic data of the RILs were analysed using SPSS 17.0. The QTL analysis was performed by integrating the genotyping data of gene-based CNMS markers mapped on the eight chickpea chromosomes with the 100-seed weight-specific phenotyping data of RIL mapping individuals and the parental genotypes. The single marker analysis and composite interval mapping functions of MapQTL 6 (Van Ooijen, 2009) with a LOD threshold score >3.0 at 1000 permutations was considered to be significant (*P*<0.05) for the identification and mapping of the major CNMS marker-associated candidate genes/QTLs regulating the 100-seed weight in chickpea.

### eQTL mapping, expression profiling, selective genotyping, molecular haplotyping, and trait association analysis

The relative expression levels of seed weight-regulating CNMS marker-associated genes at two seed developmental stages (10–20 DAP and 21–30 DAP, as described above) of each of the 190 individuals, including 30 low (100-seed weight 13.5–18g) and high (32–37.2g) seed weight homozygous RIL mapping individuals (ICC 4958×ICC 17163) along with the parental genotypes, were determined according to previously described methods. The differential expression-based genotyping and 100-seed weight-specific phenotyping data of RIL mapping individuals and genetic positions (cM) of CNMS markers mapped on eight chromosomes were analysed in the eQTL window interface module of QGene 4.3 (Joehanes and Nelson, 2008) to identify the effects and positions (cM) of the eQTLs controlling the relative expression levels of CNMS marker-associated genes for seed weight.

Additionally, the differential expression analysis of the identified seed weight-regulating CNMS marker-associated genes was performed at two seed developmental stages of 12 contrasting low (100 seed weight 5–13g) and high (31–41g)/very high (47–55g) seed weight *desi* and *kabuli* germplasm lines (ICCX-810800, ICC 2507, ICC 5845, ICC 1052, ICC 3761, ICC 4926, ICC 20268, ICC 19707, ICC 11883, ICC 20141, ICC 7410, and ICC 11301) that were selected from 300 global reference core germplasm collections of chickpea (Upadhyaya *et al.*, 2008). In addition to these, low and high/very high seed weight parental genotypes (ICC 4958 and ICC 17163) and 10 homozygous individuals of a RIL mapping population (ICC 4958×ICC 17163) were included for CNMS marker-associated gene expression profiling. To deduce the molecular basis of the differential gene expression patterns, the cDNA and genomic DNA fragments amplifying the entire coding and non-coding intronic, 3ʹ UTR, and 5ʹ upstream sequence (1000bp) components of the seed weight-governing CNMS marker-associated genes were cloned, sequenced, and compared among a selected set of 10 low and high seed weight homozygous RIL mapping individuals, parental genotypes, and 12 contrasting germplasm lines using the methods described above.

These high-quality gene sequences generated were further aligned with the corresponding genomic sequences (pseudomolecules) of two low seed weight *desi* (ICC 4958 and ICC 4951; Jain *et al.*, 2013), two very high seed weight *kabuli* (CDC Frontier and ICCV2; Varshney *et al.*, 2013; Jain *et al.*, 2013), and one low seed weight wild (PI 489777; Jain *et al.*, 2013) genotype. All of the CNMS and SNP marker genotyping information obtained from these seed weight-regulating genes based on cloned amplicon sequencing and *in silico* comparative sequence analyses was used for constitution of haplotypes and linkage disequilibrium (LD) mapping.

For the trait association analysis, the CNMS and SNP marker-based haplotype genotyping information in the genes, population genetic structure data, and seed weight-specific phenotypic information on 96 contrasting low and high/very high seed weight reference core germplasm lines were analysed (http://www.maizegenetics.net) according to Kujur *et al*. (2013, 2014). Specifically, the population structure and 100-seed weight-specific phenotyping data of 96 germplasm lines belonging to an association panel were obtained from a previous study (Kujur *et al.*, 2013). To reveal the potential of different haplotypes constituted in the CNMS marker-associated gene to regulate seed weight, differential expression profiling in two seed developmental stages of the contrasting germplasm lines representing diverse seed weight haplotype groups was performed using gene haplotype-specific primers.

## Results and discussion

### Characteristics of CNMS in chickpea genome

**Fig. 2.**
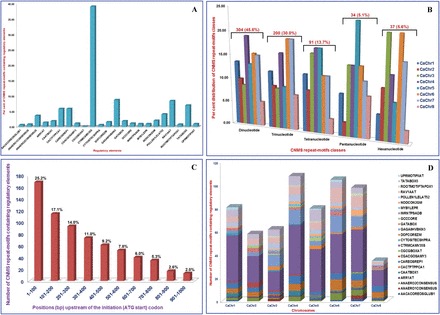
(A) Distribution frequency of CNMS repeat motifs containing gene regulatory elements in the chickpea genome. (B) Proportionate distribution of different CNMS repeat motif classes on the eight chickpea chromosomes. Dinucleotide CNMS repeats (45.6%) were the abundant microsatellite classes, followed by trinucleotide CNMS (30%) in the chickpea genome. (C) Positional distribution of CNMS repeat motifs carrying regulatory elements in the non-coding sequences upstream of the translation initiation codons (ATG) of chickpea genes. (D) Proportionate distribution of different CNMS repeat motifs containing gene regulatory elements on the eight chickpea chromosomes. The detailed characteristics of these regulatory elements and CNMS are described in Supplementary Table S2 at *JXB* online. (This figure is available in colour at *JXB* online.)

The alignment plot and intra-/intergenomic phylogenetic footprinting identified 419 and 247 (paralogous and orthologous) non-redundant/unique CNMS repeat motifs from 47 different regulatory elements that were located upstream of the initiation codons of 407 (1.4% of 28 269) and 197 (0.7% of 27 571) protein-coding genes from the *kabuli* and *desi* genomes, respectively. A total of 627 (94.2%) of the 666 CNMS repeat motifs were confined to 23 diverse known gene regulatory elements (e.g. RAV1AAT, ANAERO2CONSENSUS, CACTFTPPCA1, CAREOSREP1, GATABOX, MYB1LEPR, NODCON2GM, and POLLEN1LELAT52). The (CT)_n_ CNMS repeat motifs carrying CTRMCAMV35S (245, 39.1%) regulatory elements were the most abundant in the chickpea genome, followed by the (GA)_n_ and (CAA)_n_ repeat motifs containing GAGA8HVBKN3 (55, 8.8%) and RAV1AAT (53, 8.5%) regulatory elements, respectively (Fig. 2A). The CT/GA-rich dinucleotide repeats (304, 45.6%) were thus the predominant microsatellite classes in the CNMS (Fig. 2B). This is in accordance with previous reports on abundant pyrimidine-rich microsatellite repeat distributions in the upstream (promoter) sequences of genes in the rice and *Arabidopsis* genomes (Fujimori *et al.*, 2003; Zhang *et al.*, 2006; Parida *et al.*, 2009). Remarkably, a higher frequency (168, 25.2%) of CNMS repeats was observed near the first 100–200bp upstream from the translation initiation codons (ATG) in both the orthologous and paralogous chickpea genes (Fig. 2C). Overall, the frequency of CNMS repeats decreased gradually with the increase in their physical distances (bp) from the initiation codons of the genes, suggesting the existence of an inverse correlation between the two. This is in agreement with previous studies on genome-wide CNS, including CNMS identification in many monocot and dicot crop plants, such as rice and *Arabidopsis* (Zhang *et al.*, 2006; Parida *et al.*, 2009; Baxter *et al.*, 2012). The abundance of CNMS repeats (93%) localized upstream of the initiation codons of genes with similarities to known *cis*-regulatory elements may be due to functional constraints on the regulatory elements for imparting specialized functions in the chickpea. For example, the (CT)_n_ CNMS repeats within the TCTCTCTCT signal sequence-binding sites of known regulatory element (CTRMCAMV35S) of the *Cauliflower mosaic virus* (CaMV) 35S promoter are known to be involved in enhancing gene expression/transcriptional regulation in plants by their interactions with nuclear proteins (Pauli *et al.*, 2004). The occurrence of such CT-rich CNMS motifs in the 5ʹUTRs of plant housekeeping genes, such as those encoding the ribosomal proteins L12 and L13, suggests their involvement in a universally valid mechanism of gene regulation (Bolle *et al.*, 1996). Thus, the abundance of (CT)_n_ CNMS repeat motifs carrying CTRMCAMV35S regulatory elements in the chickpea genome is expected. Moreover, a higher frequency of (GA)_n_ CNMS repeat motifs containing GAGA8HVBKN3 regulatory elements in the chickpea genome may be due to the functional significance of such widely distributed elements in the transcriptional regulation of numerous developmental genes (Bevilacqua *et al.*, 2000; Busturia *et al.*, 2001). The (GA)_n_ CNMS repeats within the (GA)_8_ signal sequence-binding sites of known regulatory elements (GAGA8HVBKN3) that are located upstream of the start codon of the class I *Knox* homeobox gene *Bkn3* have played major roles in the transcriptional regulation of its expression, thus affecting cell growth, differentiation, and development in diverse crop plants, including barley, rice, and *Arabidopsis* (Chan *et al.*, 1998; Muller and Leutz, 2001; Santi *et al.*, 2003; Jain *et al.*, 2008; Mukherjee *et al.*, 2009). Several studies have also established the correlation between the patterns of origin and differentiation of homeobox genes in different plant groups with their increasing developmental complexities. These known regulatory elements containing homeobox genes, which are involved in many essential processes such as growth and development, are evolutionarily conserved, existing in organisms ranging from simple unicellular prokaryotes to highly complex multicellular eukaryotes, including animals and plants (Bharathan *et al.*, 1997; Kappen, 2000; Garcia-Fernandez, 2005; Holland, 2013). Thus, the higher retention of such CNMS repeats carrying regulatory elements in the chickpea genome was expected. Overall, the results suggest the non-random and strongly biased distribution of CNMS repeat motifs across the non-coding regulatory regions of chickpea genes.

### Development of CNMS markers and their genome-wide distributions

A total of 666 markers were developed, including 235 (35.3%) hypervariable class I and 43 (64.7%) class II non-redundant CNMS markers from the 5ʹURRs of 603 chickpea genes (Supplementary Table S2 at *JXB* online). All of the 666 designed gene-based CNMS markers were submitted to the freely accessible NCBI Probe database (accession nos PUID 28366769 to PUID 28367434). The genomic distributions of the 666 CNMS markers were based on their physical positions (bp) on the eight chickpea chromosomes and possessed an average marker density of 521.4kb (Supplementary Fig. S1; Supplementary Table S3). The CNMS repeat motifs carrying the CTRMCAMV35S and GAGA8HVBKN3 regulatory elements occurred predominantly on chromosomes 4 (43, 17.6%) and 6 (15, 27.3), respectively (Fig. 2D). In summary, the genomic distribution of CNMS repeat motifs carrying known regulatory elements determined on the eight chromosomes through the construction of marker-based physical maps could greatly expedite the wider use of genetical genomics for the identification and mapping of genes/QTLs (eQTLs) to control important agronomic traits in the chickpea by the rapid selection of such desired genic microsatellite markers at the chromosomal level. It would also accelerate genome-wide comparative mapping and phylogenetics, involving the chickpea and other legumes.

### Functional significance of CNMS marker-associated genes

To assess the functional significance of the CNMS marker-associated genes, the known/predicted functional characteristics of 666 CNMS repeat motifs containing regulatory elements and the putative functions of the corresponding marker-associated protein-coding genes were correlated. The analysis identified 666 CNMS marker-associated genes that corresponded to genes encoding various transcription and translation factors, metabolic enzymes, growth/development factors, and signal transduction pathway proteins (Supplementary Table S2 at *JXB* online). The 666 CNMS marker-associated genes were further functionally annotated based on their GO classifications, and the enrichment/depletion of specific GO terms in the genes was analysed statistically. The significant enrichment for TF activity (591, 88.7%, *P*≤8×10^–6^) as well as vegetative and reproductive developmental processes (425, 63.8%, *P*≤8×10^–6^) was observed for these genes. Based on the cellular component GO analysis, it was observed that the most over-represented GO term in the CNMS marker-associated genes was the nucleus. The findings from the GO term analysis and the TFBS over-representation collectively suggest the involvement of the CNMS in the transcriptional regulation of growth and developmental processes in plants (Zhang *et al.*, 2006; Freeling and Subramanium, 2009; Baxter *et al.*, 2012). Conversely, CNMS were more abundant in the genes encoding TFs and regulatory genes, which typically clustered within the known TFBS that were present upstream of the translation initiation codons.

Plants are used to relying on regulatory machinery for the integration of signals from internal and external environmental stimuli (abiotic and biotic stress) ultimately to carry out complex decision-making and response processes. Thus, the preferential selection for the retention of more of such CNMS repeat motifs containing regulatory elements/TFBS under strict control/evolutionary pressure may be essential to control the transcription of a large number of CNMS-associated genes in the chickpea. The enrichment of CNMS marker-associated gene orthologues/paralogues for TFs further implies that the CNMS and corresponding protein-coding genes have played an intrinsic role in the conserved ancient transcriptional regulatory networks, which are shared among the seven dicot species investigated. Interestingly, six (AP2, AP1, H2A.Z, ICE1, PI, and SHOOT MERISTEMLESS) of the 666 CNMS marker-associated genes were predicted to function as master regulatory TFs for controlling the mechanisms of diverse developmental processes in monocot and dicot plants, particularly in *Arabidopsis* (Supplementary Table S4 at *JXB* online). The occurrence of multiple TFBS, specifically in the 100–200bp long stretch of sequences upstream of the translation initiation codons, may facilitate manifold binding events for multiple proteins in the DNA-binding regulatory regions, which act as templates for the effective assembly of transcriptional/regulatory protein complexes to control complex developmental-responsive gene functions in the chickpea (Baxter *et al.*, 2012). Seventy-five (11.3%) of 666 CNMS-associated genes showed depletion for metabolism (at significance level *P*≤2×10^–2^) based on GO. The significant depletion of CNMS-associated gene expression involving metabolic activity reflects the limited requirements for controlling metabolism-related genes and suggests that these genes may be capable of adequately controlling the basic processes of life and survival (Zhang *et al.*, 2006). As a whole, these designed CNMS markers that are derived specifically from the regulatory sequence components of genes could essentially act as functional markers for rapidly establishing marker–trait linkages and identifying genes/QTLs (eQTLs) for many traits of agricultural importance in the chickpea.

### Amplification and polymorphic potential of CNMS markers

**Fig. 3.**
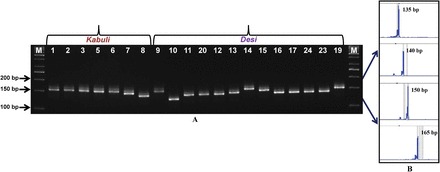
Allelic variation detected among a representative set of 20 *desi* and *kabuli* genotypes using the normal unlabelled and fluorescent dye-labelled CNMS marker designed from the flanking sequences of the (GAA)_18_ repeat motif present in the signal sequence-binding site (CTGAAGAAGAA) of the TL1ATSAR regulatory element containing the bZIP (basic leucine zipper) TF gene by gel-based assay (A) and automated fragment analyser (B), respectively. A maximum of four polymorphic alleles were amplified by the genic CNMS marker among chickpea genotypes using the gel-based assay (A) and automated fragment analyser (B), respectively. The fragment sizes (bp) of all amplified polymorphic alleles are indicated. The identities of genotypes correspond to serial numbers that are provided in Supplementary Table S1 at *JXB* online. (This figure is available in colour at *JXB* online.)

The evaluation of the amplification efficiency of 666 CNMS markers revealed clear and reproducible amplicons for 631 (94.7%) with fragments of the expected product sizes (Supplementary Table S2 at *JXB* online). The polymorphic potential of these CNMS markers was determined among a set of 25 *desi*, *kabuli*, and wild genotypes using both gel-based assay and a fluorescent dye-labelled automated fragment analyser (Fig. 3). A total of 256 (40.6%) of the 631 CNMS markers showed polymorphism among the 25 genotypes. The extent of polymorphism detected by the genic CNMS markers between the *desi* and *kabuli* genotypes (38.7%) was higher compared with that obtained within *desi* and *kabuli* (35.8%). The CNMS markers overall detected 1–6 alleles (average of four alleles) per marker locus (40.6% polymorphic, PIC 0.51) with a total of 996 alleles in the 25 chickpea genotypes. The intraspecific (37.6%) polymorphism as detected by the CNMS markers among the 25 genotypes is slightly higher/comparable with previous estimates using the EST-derived genic microsatellite markers and TF gene-based markers (25–37%; Choudhary *et al.*, 2009, 2012; Nayak *et al.*, 2010; Gujaria *et al.*, 2011; Kujur *et al.* 2013), but lower than the levels reported for the genomic (40–65%; Sethy *et al.*, 2006; Bharadwaj *et al.*, 2011; Gaur *et al.*, 2011) microsatellite markers. The higher polymorphic potential of the CNMS markers compared with the EST-/gene-based markers may be due to their exclusive derivation from the URRs of the genes, which are under less selection pressure. Moreover, this finding is possibly due to the design of such markers from abundant polymorphic classes of dinucleotide CNMS repeat motifs (45.6%) carrying regulatory elements, which are more prone to replication slippage (Parida *et al.*, 2009, 2010*a*, *b*).

**Fig. 4.**
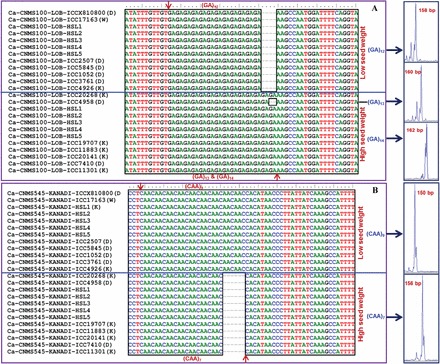
Multiple sequence alignments of cDNA and genomic DNA amplicons amplified from 10 low and high/very high seed weight homozygous RIL mapping individuals, parental genotypes (ICC 4958 and ICC 17163), and 12 contrasting germplasm lines using the two seed weight-regulating differentially expressed CNMS marker-associated LOB-domain proten- (A) and KANADI protein-encoding (B) genes validated the presence of microsatellite repeat motifs. The presence of variable CNMS repeat units, such as (GA)_n_ and (CAA)_n_, in the signal sequence-binding sites of the GAGA8HVBKN3 and RAV1AAT regulatory elements of these two genes, respectively, which differentiated all of the low seed weight germplasm lines, parents, and homozygous mapping individuals (amplifying 158bp and 150bp alleles) from the high/very high seed weight germplasm lines, parents, and homozygous RILs (160/162bp and 156bp alleles) was evident. HSL, homozygous lines. (This figure is available in colour at *JXB* online.)

The cloning, sequencing, and high quality sequence alignments of size variant amplicons of each of the 12 CNMS markers (Table 1) from the selected genotypes revealed the presence of expected microsatellite repeat motifs. They also corresponded exactly with the differences in the numbers of repeat units in the signal sequences of the regulatory element-binding sites of the genes and the fragment length variations of the sequenced alleles (Fig. 4). Several studies have correlated such fragment length polymorphism with the expansion/contraction of CNMS repeat units and the regulation of gene expression for many agronomic traits in diverse crop plants (Zhang *et al.*, 2006; Parida *et al.*, 2009). It would thus be interesting to study the functional significance of CNMS markers based on their repeat expansion/contractions in the known regulatory elements/TFBS of protein-coding genes to understand further the molecular mechanisms regulating complex quantitative traits in the chickpea.

**Table 1.** Characteristics of 17 informative CNMS marker-associated genes showing strong seed tissue-/developmental stage-specific expression in chickpea

CNMS marker IDs^*a*^	NCBI Probe IDs	Chromosome physical positions (bp)	Microsatellite repeat motifs	Known regulatory elements/TFBS	Signal sequences of regulatory element- binding sites	CNMS repeat variation between low and high seed weight contrasting genotypes	Forward primer (5ʹ–3ʹ)	Reverse primer (5ʹ–3ʹ)	Annealing temperature (°C)	Amplified fragment size (bp) range	Putative function
Ca-CNMS98^*b*^	28367433	CaChr2-3541793	(AG)12	GAGA8HVBKN3	(GA)8	(AG)12 and (AG)10	TACCACTTTTATACGCTGCAC	ATGAATGAACGAATGTGACTC	56	145–149	HAIRY MERISTEM 3 (*HAM3*) cell division/ differentiation and GRAS family transcription factor
Ca-CNMS99^*b*^	28367434	CaChr2-3544818	(GA)10	GAGA8HVBKN3	(GA)8	(GA)10 and (GA)12	CCTCTGAAATGGGACTGTT	AACACTTCCCCACACAAAC	55	137–141	ERF/AP2 transcription factor family
Ca-CNMS100^*b*,,*c*^	28366771	CaChr2-3547678	(GA)13	GAGA8HVBKN3	(GA)8	(GA)12 and (GA)13/(GA)14	ATGGAGGCGAATATATAGGAG	TTTTAAGAAACAACTGCGTTC	55	158–164	LOB domain- containing protein 40 (*LBD40*)
Ca-CNMS242^*b*^	28366928	CaChr4-9449939	(TTCT)3	CTRMCAMV35S	TCTCTCTCT	(CT)6/(CT)7 and (CT)8	ATTTTGATTCTCCTCAGATCC	GAAATCTTTTGCTGCTTATGA	55	141–145	Myb-like transcription factor family protein
Ca-CNMS310^*c*,*d*^	28367004	CaChr4- 41986507	(AG)6	CTRMCAMV35S	TCTCTCTCT	(CT)6 and (CT)8	TTTTTCTTCTCACTTCGATCA	TAGAAACGAAAGCTGAAAAGC	55	123–127	HTA9, a histone H2A protein
Ca-CNMS316	28367010	CaChr4- 44655063	(AG)10	GAGA8HVBKN3	(GA)8	(AG)10 and (AG)12	GGACACACAACAGAGAGAAAA	ACGAGGATTGTAAGGAGTACC	55	163–167	AL4 the Alfin-Like family of nuclear- localized PHD domain and Domain of unknown function (DUF3594)
Ca-CNMS352	28367050	CaChr5- 27283973	(ATGGT)3	S1FBOXSORPS1L21	ATGGTA	(ATGGT)3 and (ATGGT)2	TCCCATCAAGATGCTAAAATA	AATCAATCTTTGAGTTGTTGC	55	149–154	*SHY2/IAA3* regulates multiple auxin responses and AUX/IAA transcription factor family
Ca-CNMS380^*b*^	28367081	CaChr5- 34505910	(TC)8	CTRMCAMV35S	TCTCTCTCT	(CT)8 and (CT)7	CTTCACCCCTTGGATTTT	GTTGTTGTTGATGCTGTGAC	55	130–132	Med15 subunit of Mediator complex and LEUNIG, a putative transcriptional co-repressor that regulates AGAMOUS expression during reproductive development
Ca-CNMS402^*b*^	28367106	CaChr5- 42499426	(AG)6	CTRMCAMV35S	TCTCTCTCT	(CT)6 and (CT)7	ATGGTTTTGCTCACATTCTTA	AACTCACACAGAACACTGCTT	55	149–151	MADS box transcription factor family
Ca-CNMS506	28367221	CaChr6- 45703332	(GA)18	GAGA8HVBKN3	(GA)8	(GA)18 and (GA)15	GACATGGTTCGATTTGGAT	CACACTCACTCTCTTCTTCTTCT	56	153–159	*WRKY* transcription factor
Ca-CNMS539	28367257	CaChr7-4624457	(AG)8	GAGA8HVBKN3	(GA)8	(AG)8 and (AG)6	GAAGTTCTGCCACTGAGTTC	TTAGGAACAGCCTTGTCAAT	55	153–159	Pollen-specific transcription factor and Response regulator receiver domain-containing protein
Ca-CNMS544^*b*^	28367263	CaChr7-6052379	(CT)11	CTRMCAMV35S35S	TCTCTCTCT	(CT)11 and (CT)9	TCTCTCATCCTCTTTCTTTCC	GATCCAAAGAATGGAATAACC	55	147–151	KH domain- containing protein/ zinc finger family protein
Ca-CNMS545^*b*,*c*^	28367264	CaChr7-6054880	(CAA)7	RAV1AAT	CAACA	(CAA)7 and (CAA)9	CAAGCTCACACTGAACTCTCT	GTTGTTGAGTTGTGGTGATTT	55	150–156	KANADI protein (*KAN*)
Ca-CNMS546^*b*^	28367265	CaChr7-6057925	(TC)7	CTRMCAMV35S35S	TCTCTCTCT	(TC)7 and (TC)9	TGAGAACAAGACCAGAGTCAC	GTGTTATTGTTGGACCTCAAG	55	140–144	Myb-like transcription factor family protein controlling reproductive development
Ca-CNMS547^*b*^	28367266	CaChr7-6060830	(CT)10	CTRMCAMV35S35S	TCTCTCTCT	(CT)10 and (CT)8	ATTCTCCTCATAAGCCATTCT	TTCTCGATCTATACGTCAACC	55	150–154	A Class II KN1-like homeodomain transcription factor
Ca-CNMS562^*b*^	28367283	CaChr7- 12021223	(ACACAA)4	CANBNNAPA	CNAACAC	(ACACAA)4 and (ACACAA)6	CCTCTCTTATTTTGTTTGTCAAC	TTCCTTCGAAATTGAGTAAAG	55	153–165	WRKY DNA- binding protein 15 (*WRKY15*)
Ca-CNMS642	28367372	CaChr8-3997981	(AAG)4	CTRMCAMV35S	TCTCTCTCT	(TC)9 and (TC)13	CACCTTCCATCTTCTTCTTCT	TGACAAACTTAATGGGAGAGA	55	147–155	DREB subfamily and ERF/AP2 transcription factor family

^*a*^ Detailed information on CNMS markers are provided in Supplementary Table S2 at JXB online.

^*b*^ Validated through cloned amplicon sequencing.

^*c*^ CNMS marker-associated genes validated through differential expression profiling, QTL and eQTL mapping, selective genotyping, and association analysis.

^*d*^ Ultra-CNMS marker-associated gene functioning as master regulator transcription factor.

### Differential expression profiling of CNMS marker-associated genes

A set of 256 informative CNMS marker-associated genes (showing polymorphisms among the *desi* and *kabuli* genotypes) were used for differential expression profiling in six different vegetative and reproductive tissues of ICC 4958 via semi-quantitative and quantitative RT-PCR assays. Of these, 220 (85.9%) genes were expressed in all of the chickpea tissues that were analysed. A higher proportion (98.2%, 216) of CNMS marker-associated genes were differentially expressed (≥2-fold) in at least one of the tissues. For example, 117 genes were differentially up- (76 genes)-and down- (41) regulated (≥2-fold) in the seed tissue of ICC 4958 compared with its three vegetative tissues (root, leaf, and shoot). The maximum preferential expression was observed in the flower bud, followed by the root, young pod, and seed when compared with the leaf (Supplementary Fig. S2 at *JXB* online). Twelve CNMS marker-associated genes (≥5-fold) showed tissue-specific expression in the flower bud, whereas four, three, and two genes exhibited expression in the root, young pod, and seed, respectively.

**Fig. 5.**
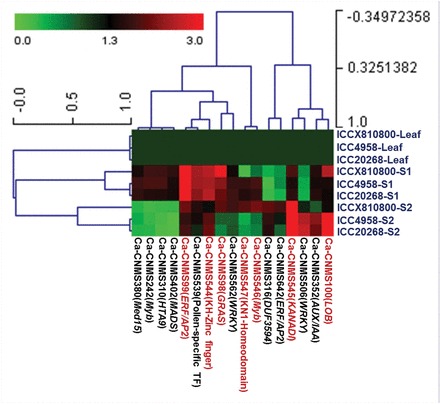
Hierarchical cluster display of the expression profile for 17 CNMS marker-associated genes showing high levels of seed-specific expression at two seed developmental stages/tissues compared with vegetative leaf tissues of three contrasting low and high/very high seed weight chickpea genotypes (ICCX-810800, ICC 4958, and ICC 20268). The scale at the top represents the average log of the signal expression values (expression levels) of the genes in the various tissues and developmental stages. The tissues/seed developmental stages of the contrasting genotypes and CNMS marker-associated genes that were used for the expression profiling analysis are indicated on the right side and top of the expression map, respectively. Details of CNMS marker-associated genes are given in Table 1. Seven CNMS marker-associated genes harbouring the seed weight QTLs/eQTLs are marked with arrows. S1, seed development stage 1 (10–20 DAP); S2, seed development stage 2 (21–30 DAP). The expression values across different tissues/developmental stages of genotypes were normalized against the endogenous control *elongation factor-1 alpha* in the quantitative RT-PCR assay. The gene expression in leaf tissues of all the genotypes was considered as the reference calibrator and assigned as 1. (This figure is available in colour at *JXB* online.)

To understand the regulation of CNMS marker-associated genes showing differential expression during seed development, a set of 51 genes showing preferential/specific expression (≥3-fold) in the seeds of ICC 4958 compared with the leaf were selected. These genes were further analysed at two seed developmental stages by comparing three low and high/very high seed weight chickpea genotypes, including ICCX-810800, ICC 4958, and ICC 20268, using both semi-quantitative and quantitative RT-PCR assays. Forty-seven (92.2%) of 51 genes were preferentially expressed in at least one of the seed developmental stages compared with the leaf in the three chickpea genotypes. Seventeen (36.2%) genes exhibited seed tissue- and/developmental stage-specific expression (≥4-fold) in contrast to their respective vegetative tissue (leaf) (Fig. 5). Recently, the genome-wide transcriptome profiling of diverse *desi*, *kabuli*, and wild chickpea genotypes representing different tissues/developmental stages has identified a larger set of differentially expressed transcripts/genes with preferential and tissue-specific expression (Garg *et al*., 2011*a*, *b*; Hiremath *et al.*, 2011; Agarwal *et al.*, 2012; Jhanwar *et al.*, 2012). To narrow down the functionally relevant gene targets rapidly from such large numbers of differentially expressed genes at specific developmental stages/tissues, a marker-based integrated genetic and association mapping approach combining differential transcript profiling may be a feasible strategy. In this context, a set of preferentially expressed informative CNMS marker-associated genes, including those showing fragment length polymorphism and specific expression in different tissues/seed developmental stages of contrasting low and high/veryhigh seed weight genotypes identified in this study, would be of significance. These smaller sets of screened informative CNMS marker-associated genes could thus serve as targets for rapid genetic/association mapping for the identification of novel regulatory elements and alleles in the genes/QTLs (eQTLs) controlling seed weight and the understanding of their regulatory functions during seed development.

### CNMS marker-based functional transcript map and mapping of genes/QTLs for seed weight

A total of 238 gene-based CNMS markers showing parental polymorphism between ICC 4958 and ICC 17163 (parents of a RIL mapping population) were selected for the construction of a genetic linkage map and seed weight-specific QTL mapping. These markers were genotyped among 190 individuals of a RIL mapping population (ICC 4958×ICC 17163) to construct the genetic linkage map (transcript map) of the chickpea. The linkage analysis of marker genotyping data enabled the genetic mapping of all 238 CNMS markers across the eight chromosomes according to their physical positions (bp) on the respective chickpea LGs/chromosomes (Supplementary Fig. S3 at *JXB* online). The transcript map consisting of the eight chromosomes covered a total map length of 766.9 cM with an average intermarker distance of 3.22 cM (Table 2). The longest LG with a map length of 140.3 cM (40 CNMS markers) was observed in chromosome 4, followed by chromosome 1 (27 markers spanning 124.3 cM). Chromosome 8 had the shortest LG, spanning 63.5 cM (19 markers). The average intermarker distance for the transcript map varied from 2.31 cM (chromosome 6) to 4.60 cM (chromosome 1) (Table 2). This is in agreement with the range of average intermarker distances that was estimated (1.77–8.01 cM) previously for microsatellite marker-based genetic linkage maps of chickpea (Winter *et al.*, 2000; Nayak *et al.*, 2010; Gaur *et al.*, 2011; Gujaria *et al.*, 2011; Choudhary *et al.* 2012; Kujur *et al.* 2013). The genic CNMS marker-based transcript map constructed in the present study would thus expedite the identification and targeted mapping of genes/QTLs (eQTLs), particularly those that are associated with yield and stress tolerance in the chickpea.

**Table 2.** CNMS marker-associated genes mapped on the eight chromosomes of the chickpea [Cicer arietinum (Ca)] transcript map

Linkage groups/chromosomes	Mapped CNMS markers	Map length covered (cM)	Average intermarker distance (cM)
CaLG(Chr)1	27	124.3	4.60
CaLG(Chr)2	20	78.9	3.94
CaLG(Chr)3	28	70.6	2.52
CaLG(Chr)4	40	140.3	3.50
CaLG(Chr)5	29	103.4	3.56
CaLG(Chr)6	37	85.4	2.31
CaLG(Chr)7	38	100.5	2.64
CaLG(Chr)8	19	63.5	3.34
Total	238	766.9	3.22

**Fig. 6.**
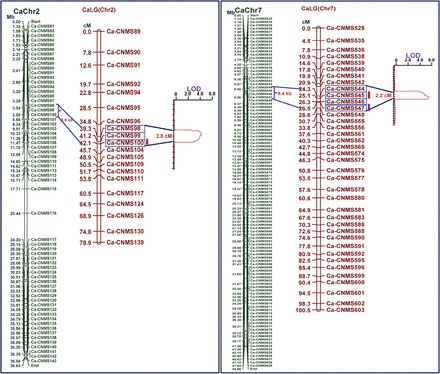
Integration of the genetic and physical maps of the target genomic regions underlying seed weight QTLs and eQTLs identified on chickpea chromosomes 2 and 7. The target seed weight *cis*- and *trans*-eQTLs (LOD >5.0) for three CNMS marker-associated genes identified on chromosomes 2 and 7 are indicated. The genetic (cM) and physical (bp) distances and identities of the CNMS marker loci integrated on the chromosomes are indicated on the left and right sides of the chromosomes, respectively. (This figure is available in colour at *JXB* online.)

Significant differences in 100-seed weight between the parental chickpea genotypes and the RIL mapping individuals (13.5–37.2g) was observed based on the ANOVA. A normal frequency distribution of seed weight among the 190 RIL mapping individuals and parental genotypes (Supplementary Fig. S4 at *JXB* online) indicated the utility of the developed mapping population for seed weight QTL mapping studies. The QTL mapping of 238 CNMS markers in the RIL mapping population allowed for the identification of two major and significant (LOD >4.0) QTLs on chromosomes 2 [phenotypic variation explained (PVE) *R* ^2^=27.8%] and 7 (*R* ^2^=23.6%) in association with the 100-seed weight (Fig. 6). These target seed weight QTL regions identified on chromosomes 2 (39.3–42.1 cM) and 7 (24.3–25.6 cM) included three (Ca-CNMS 98–100) and four (Ca-CNMS 544–547) gene-based CNMS markers, respectively (Fig. 6). To establish correlations between the genetic map and the sequence-based physical map, the seed weight-associated target regions identified by genetic/QTL mapping were combined with those that were derived from the latest available genomic sequence information for the chickpea (Varshney *et al.*, 2013). The integration of the genetic and physical maps indicated that the seed weight QTL regions identified on chromosomes 2 and 7 corresponded to physical intervals of ~5885bp and 8451bp, respectively (Fig. 6). Interestingly, the seven CNMS marker-associated genes harbouring the seed weight QTL regions showed strikingly high seed-specific expression (≥4-fold) compared with the leaf in the three low and high seed weight chickpea genotypes (ICCX-810800, ICC 4958, and ICC 20268) based on differential expression profiling (Fig. 5). Therefore, these seven genes were selected as candidates for dissecting the complex quantitative seed weight in the chickpea.

### CNMS marker-based eQTL mapping, expression profiling, and selective genotyping

Seven CNMS marker-associated genes selected based on their seed-specific expression and localization in seed weight QTL regions were further analysed for eQTL mapping, large-scale expression profiling, and selective genotyping. A differential expression analysis of the seven seed weight-regulating CNMS marker-associated genes at the two seed developmental stages of the parental genotypes (ICC 4958 and ICC 17163) and 190 individuals, including 30 low and high seed weight homozygous RIL mapping individuals, was performed. The correlation of differential expression profiling with CNMS marker-based seed weight QTL mapping identified significant (LOD >5.0) eQTLs for three CNMS marker-associated genes (Ca-CNMS 99, 100, and 545) on chromosomes 2 and 7 governing 100-seed weight (Fig. 6). The target eQTLs identified for the three genes explained a total of 26.3% of the variation in their expression levels (Fig. 6). One of the high seed weight parental genotypes (ICC 4958) exhibited increased relative expression levels during seed development for eQTLs of all three of the CNMS marker-associated genes. The mapping of a single eQTL in the URRs of two CNMS marker-associated genes (Ca-CNMS 100 and 545) suggested that allelic polymorphism of microsatellite repeat units at known regulatory element regions/TFBS of such genes directly impact their expression levels. Despite the quantitative genetic inheritance patterns of the seed weight in the chickpea, it was observed that each of the two seed weight-governing genes was independently regulated by a single eQTL for chromosomes 2 and 7, with their major effect (*R* ^2^=25.7%) being trait expression variation. This correlates well with earlier eQTL mapping studies involving the dissection of complex quantitative agronomic traits in maize (Schadt *et al.*, 2003) and barley (Potonika *et al*., 2006). The co-localization of CNMS marker-associated TF genes (Ca-CNMS100/LOB-domain-containing protein and Ca-CNMS545/KANADI protein) and their eQTLs (LOD >5.0) based on congruent genetic map positions for two chromosomes (Chr 2, 42.1 cM; and Chr 7, 25.1 cM) indicated the *cis*-regulation of these CNMS-containing known regulatory elements for controlling differential gene expression involving seed weight in the chickpea. Such *cis*-regulation-mediated differential gene expression has commonly been observed in mammals and plants at higher LOD scores (Doss *et al.*, 2005; Hubner *et al.*, 2005; Salvi *et al.*, 2007; Boerjan and Vuylsteke, 2009; Terpstra *et al.*, 2010). However, the genetic position (Chr 2, 41.2 cM) of the single remaining CNMS marker-associated gene [Ca-CNMS 99/AP2/ERF (APETALLA 2/ethylene response factor) TF] and its eQTL was not congruent. Interestingly, the eQTL of this gene was positioned with another CNMS marker-associated gene (Ca-CNMS 547/homeodomain TF) that was mapped to chromosome 7 (Chr 7, 26.5 cM), thus indicating that both of these genes are *trans-*regulated for the control of the differential expression of seed weight. The four *cis* and *trans* eQTLs regulating seed weight identified on two chromosomes by the differential expression profiling were well correlated and situated within the genomic regions harbouring seed weight QTLs (as detected in the QTL mapping studies using CNMS marker-based genotyping information) (Fig. 6). These findings confirm that four seed weight-governing CNMS marker-associated genes identified at both the QTL and eQTL target regions for seed weight could serve as potential candidates for the regulation of seed weight expression in the chickpea.

**Fig. 7.**
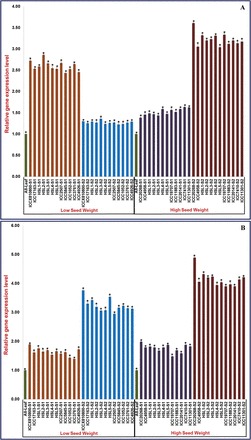
Differential expression profiling of seed weight-regulating CNMS marker-associated LOB-domain protein- (A) and KANADI (B) protein-encoding genes at two seed developmental stages of 10 low and high/very high seed weight homozygous RIL mapping individuals, parental genotypes (ICC 4958 and ICC 17163), and 12 contrasting germplasm lines compared with their vegetative leaf tissues using quantitative RT-PCR assay. HL, homozygous lines; S1, seed development stage 1 (10–20 DAP); S2, seed development stage 2 (21–30 DAP). The expression values across different tissues/developmental stages of germplasm lines, parents, and mapping individuals were normalized against the endogenous control *elongation factor-1 alpha* in the quantitative RT-PCR assay. The gene expression in leaf tissues (indicated as All-Leaf) of all the germplasm lines and mapping individuals was considered as the reference calibrator and assigned as 1. Each bar represents the mean (± SE) of three independent biological replicates with two technical replicates for each sample used in the quantitative RT-PCR assay. *Significant differences in expression of CNMS marker-associated genes at two seed developmental stages of low and high seed weight germplasm lines, parents, and mapping individuals as compared with leaf at *P*<0.01 (LSD-ANOVA significance test). (This figure is available in colour at *JXB* online.)

To validate further the results of the eQTL mapping and the *cis*-regulation of the genes, the eQTLs that were identified for the four CNMS marker-associated genes were analysed for differential expression at two seed developmental stages of low (100-seed weight 5–13g) and high/very high (47–55g) seed weight in 12 contrasting germplasm lines along with parents (ICC 4958 and ICC 17163) and 10 homozygous RIL mapping individuals using semi-quantitative and quantitative RT-PCR assays. Two of these genes (*cis*-regulated eQTLs) (one on chromosome 2 and another on chromosome 7) were differentially expressed during seed development (as compared with the leaf). The two genes remaining showed almost equal levels of gene expression at the seed developmental stages of both the low and high/very high seed weight germplasm lines, parents, and mapping individuals. The CNMS marker-associated gene [LOB-domain protein 40 (*LBD40*)] on chromosome 2 was down-regulated (at least 2-fold) at the seed developmental stages of the low seed weight germplasm lines, parents, and mapping individuals (as compared with the leaf), while for the high/very high seed weight germplasm lines, parents, and mapping individuals, this gene was up-regulated during seed development (as compared with the leaf) (Fig. 7A). In contrast, another CNMS marker-associated gene [KANADI protein (*KAN*)] on chromosome 7 was up-regulated (at least 2-fold) at the seed developmental stages of both the low and high/very high seed weight germplasm lines, parents, and mapping individuals under study (Fig. 7B).

The comparison of 17 seed tissue-/developmental stage-specific genomic and cDNA sequences, including two seed weight-regulating differentially expressed CNMS marker-associated genes (LOB-domain protein- and KANADI protein-encoding genes) (Fig. 4) in the low and high/very high seed weight *desi* and *kabuli* germplasm lines and homozygous RIL mapping individuals and parental genotypes revealed the presence of the expected microsatellite repeat motif sequences. The detailed comparative sequence analysis with the existing genome sequence database of five low and high/very high seed weight genotypes (ICC 4958, ICC 4951, CDC Frontier, ICCV2, and PI489777) confirmed the occurrences of variable numbers of microsatellite repeat units in the known regulatory elements/TFBS of all 17 of the CNMS marker-associated genes that differentiated at least two of the low and high/very high seed weight contrasting germplasm lines and mapping individuals studied (Table 1). Notably, the presence was observed of variable repeat units, such as (GA)_n_ and (CAA)_n_, in the signal sequence binding sites of the regulatory elements (GAGA8HVBKN3 and RAV1AAT) of two seed weight-regulating differentially expressed CNMS marker-associated genes (LOB-domain protein- and KANADI protein-encoding genes), respectively, which differentiated all of the low seed weight germplasm lines, homozygous RIL mapping individuals, and parental genotype (ICC 17163) from the high/very high seed weight germplasm lines, parent (ICC 4958), and homozygous RILs (Fig. 4). These findings thus infer the significant correlation between fragment length polymorphism due to microsatellite repeat unit variability at known regulatory elements/TFBS of genes with their differential expression during seed development in the chickpea. The expansion and contraction of microsatellite repeats in the regulatory regions of genes have significance in controlling the gene expression of many traits, including amylose content in rice (Bao *et al.*, 2002), protein quality in maize (Dresselhaus *et al.*, 1999), and light and salicylic responses in *Brassica* (Zhang *et al.*, 2006).

### Molecular haplotyping and association analysis in seed weight-regulating CNMS marker-associated genes

A genetic association analysis was conducted by correlating the genotyping data of two seed weight-regulating CNMS marker-associated TF genes with the seed weight-specific phenotyping information (5–55g) of 96 germplasm lines (association panel). This analysis revealed a significantly high degree of association of two of these CNMS markers derived from the LOB-domain protein- (*P*=1.2×10^–4^ with *R* ^2^=0.39) and KANADI protein- (*P*=3.1×10^–2^ with *R* ^2^=0.18) encoding TF genes with 100-seed weight in the chickpea. The estimation of marker allele effects in these two CNMS marker-associated genes depicted their overall strong effect on seed weight regulation in the germplasm lines (Fig. 4). There was a >3-fold increase in the marker allele effects of the LOB-domain protein- and KANADI protein-encoding TF gene-derived CNMS markers, amplifying 160/162bp and 156bp alleles in the high/very high seed weight germplasm lines compared with the low seed weight germplasm lines amplifying 158bp and 150bp alleles, respectively (Fig. 4). The differential marker allele effects of the CNMS markers in the regulatory regions of the genes thus reflect their significance for rapid trait association analyses in the chickpea. Collectively, the results of the differential expression profiling, seed weight QTL and eQTL mapping, selective genotyping, and association analysis identified one regulatory element in each of the two seed weight-governing CNMS marker-associated TF genes (LOB-domain protein- and KANADI protein-encoding genes) controlling seed weight in the chickpea. The significance of these two identified TF genes was clearly understood by correlating their differential expression profiling and the *cis*-regulation of the varied CNMS repeats in the known regulatory elements/TFBS (GAGA8BKN3 and RAV1AAT) with strong biased CNMS marker allele effects in the contrasting high/very high seed weight rather than low seed weight RILs, segregating mapping individuals and germplasm lines.

**Fig. 8.**
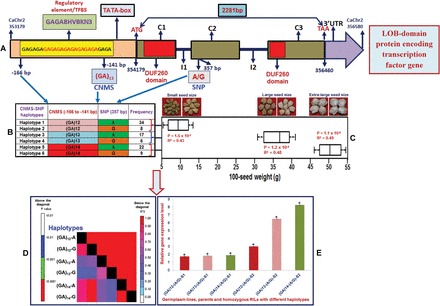
The seed weight-specific molecular haplotyping, LD mapping, genetic association analysis, and gene haplotype-specific expression profiling of a CNMS marker-associated LOB-domain protein-encoding TF gene, validating its potential for seed weight regulation in chickpea. The genotyping of one CNMS marker in the regulatory element/TFBS and one synonymous SNP (A/G) identified in the coding sequence component of this TF gene (A) among 96 contrasting low and high seed weight germplasm lines (association panel) constituted six haplotypes (B). Three specific haplotype (B) groups [(GA)_12_-A/G], [(GA)_13_-A/G], and [(GA)_14_-A/G], which were represented by 42, 23, and 31 germplasm lines showed significant association potentials for low (100 seed weight varied from 5g to 13g) and high (31–41g)/very high (47–55g) seed weight differentiation (C), respectively. (D) The LD mapping with genotyping data of six haplotypes produced higher LD estimates (*r* ^2^>0.20 and *P*<0.0001) across the entire 3400bp sequenced region of the TF gene. The columns below the diagonal indicate the correlation frequency (*r* ^2^) among a pair of six different haplotypes constituted in a gene, whereas columns above the diagonal specify the *P*-value (*P*<0.01) of LD estimates (*r* ^2^) for these haplotype combinations at 1000 permutations. (E) The differential up-regulated expression of the haplotypes of this gene in seed developmental stages of low and high/very high seed weight germplasm lines, parents, and homozygous RIL mapping individuals representing haplotype groups compared with the leaf was also observed. Each bar represents the mean (± SE) of three independent biological replicates with two technical replicates for each sample used in the quantitative RT-PCR assay. *Significant differences in expression of gene haplotypes at two seed developmental stages of low and high seed weight germplasm lines, parents, and mapping individuals as compared with leaf at *P*<0.01 (LSD-ANOVA significance test). (This figure is available in colour at *JXB* online.)

To perform the seed weight-regulating gene haplotype-specific association mapping, the genotyping information of the CNMS marker in the regulatory element/TFBS and one synonymous SNP identified in the coding sequence of the CNMS marker-associated LOB-domain protein-encoding TF gene (showing the potential for strong association with seed weight) among 96 germplasm lines (association panel) were utilized. It enabled six haplotypes in the entire 3400bp sequenced amplicon of this gene to be constituted (Fig. 8). The haplotype-specific LD mapping and genetic association analysis using these CNMS-SNP marker-based haplotypes in the LOB-domain protein-encoding TF gene revealed a higher association potential (*P*=1.7×10^–5^ with *R* ^2^=0.47) of the gene haplotypes with seed weight compared with that of the individual CNMS marker (Fig. 8). This suggests that the CNMS-SNP marker-based gene–haplotype combinations efficiently provide higher LD resolution (*r* ^2^>0.20 with *P*<0.0001) and enhance the association potential of genes for seed weight in the chickpea. Interestingly, three specific haplotype groups, [(GA)_12_-A/G], [(GA)_13_-A/G], and [(GA)_14_-A/G], differentiating the low (42 germplasm lines, 100-seed weight varied from 5g to 13g), high (23, 31–41g), and very high (31, 47–55) seed weight chickpea germplasm lines were identified, respectively (Fig. 8). These three gene haplotypes thus showed significantly higher association potentials for low (*P*=1.5×10^–3^ with *R* ^2^=0.43) and high (*P*=1.2×10^–4^ with *R* ^2^=0.48)/very high (*P*=1.1×10^–6^ with *R* ^2^=0.49) seed weight differentiation among the chickpea genotypes. The differential expression profiling at the two seed developmental stages of the contrasting germplasm lines representing the three varying seed weight haplotype groups of the CNMS marker-associated gene (LOB-domain protein-encoding TF) revealed their up-regulated expression (at least 2.5-fold), specifically at the seed developmental stages of 12 germplasm lines, parents, and 10 homozygous RIL mapping individuals (selected randomly from each of the low and high/very high seed weight haplotype groups) compared with that of the leaf (Fig. 8). These findings confirm the association potential of (GA)_n_ CNMS repeat motifs carrying GAGA8HVBKN3 regulatory elements and the haplotypes identified in the LOB-domain protein-encoding TF gene for seed weight differentiation and their implications in deciphering transcriptional regulatory gene functions during seed development in the chickpea.

The (GA)_n_ CNMS repeat motif in the regulatory element region (GAGA8HVBKN3) of the LOB-domain protein-encoding TF gene (*LBD40*, plant-specific LOB TF gene family) has been implicated in a variety of transcriptional activities that occur during developmental processes in plants, such as *Arabidopsis* and rice (Shuai *et al.*, 2002; Yang *et al.*, 2006; Husbands *et al.*, 2007; Majer and Hochholdinger, 2011). Likewise, the involvement of the (CAA)_n_ CNMS repeat motif in the regulatory element region (RAV1AAT) of the KANADI protein-encoding TF gene (higher plant-specific class III HD-Zip gene family) in controlling growth and development has also been reported in *Arabidopsis* (Hawker and Bowman, 2004; Izhaki and Bowman, 2007; Ilegems, 2010). Two seed weight-governing *cis*-regulated CNMS marker-associated genes (LOB-domain protein- and KANADI protein-encoding genes), identified and validated by integrating the differential expression profiling, QTL and eQTL mapping, selective genotyping, haplotyping, and association mapping, were analysed in detail using the AGRIS regulatory network database (AtRegNet; http://arabidopsis.med.ohio-state.edu). The direct interaction of the (CAA)_n_ CNMS repeat motifs carrying the C-terminal domain of the RAV1 TF DNA-binding protein (B3; *VP1*/ABI3 TF) that is present in the upstream sequences of KANADI protein-encoding genes was observed with one of the seed weight-regulating CNMS marker-associated LOB-domain protein-encoding TF genes, *LBD40* (as identified in the present study) along with the *MADS* (*SEPALLATA3*) TF gene. These interacting genes have been established to be key regulators of embryogenesis during seed development in *Arabidopsis* (Zheng *et al.*, 2009; Le *et al.*, 2010; Agarwal *et al.*, 2011; Majer and Hochholdinger, 2011). Therefore, it would be interesting to identify the upstream (upstream factors interacting/regulating the TF genes) and downstream (TFs regulating/interacting with the downstream structural genes) targets of these two seed weight-regulating CNMS marker-associated TF genes and their interactions specifically at CNMS repeat-containing known regulatory element-binding sites for understanding the transcriptional regulation of such potential regulatory elements (alleles and haplotypes) during seed development in the chickpea.

In summary, the 666 informative functional CNMS markers possessing relatively high intraspecific polymorphic potentials that were developed from the non-coding URRs of genes have been shown to have pronounced applicability in chickpea genome analyses, including the construction of a functional transcript map and QTL/eQTL mapping and for the quantitative assessment of seed weight (Supplementary Fig. S5 at *JXB* online). Significantly, most of these mapped CNMS markers controlling *cis-*/*trans*-regulatory gene expression and functions are based on differential expression profiling, strong biased marker allele/haplotype effects, and fragment length polymorphism due to varied CNMS repeats at TFBS, which could expedite the comprehensive use of integrative genetical genomics in the chickpea. Consequently, it would pin down the potential candidate gene targets (regulatory elements, alleles, and haplotypes) underlying QTLs (eQTLs) involved in transcriptional regulation of complex seed weight. The CNMS markers (allelic variants and haplotypes) in the seven genes, including the two (LOB-domain protein- and KANADI protein-encoding genes) harbouring seed weight QTLs/eQTLs identified in this study, once validated via large-scale fine-mapping/map-based gene cloning and transgenics (overexpression/knock-down), could be utilized for marker-assisted varietal improvement in chickpea. Although good candidates, the causal genes for seed weight QTLs/eQTLs may not necessarily be among the seven CNMS marker-associated genes found in the confidence intervals of QTLs/eQTLs.

## Supplementary data

Supplementary data are available at *JXB* online.

Figure S1. A physical map of the chickpea constructed using 666 CNMS marker-associated genes with an average density of 521.4kb.

Figure S2. Hierarchical cluster display representing the expression profile of 220 CNMS marker-associated genes that were differentially expressed in six different vegetative and reproductive tissues of the chickpea genotype ICC 4958.

Figure S3. A functional transcript map (genetic linkage map) of the chickpea (ICC 4958×ICC 17163) constructed using 238 parental polymorphic CNMS marker-associated genes..

Figure S4. The frequency distribution of 100-seed weight (g) among 190 individuals of a RIL mapping population (ICC 4958×ICC 17163) revealed the significant variation of seed weight between parental genotypes and among mapping individuals, and depicted a goodness-of-fit to the normal distribution.

Figure S5. Characteristics, functional significance, and utility of genome-wide CNMS marker-associated genes for large-scale genotyping applications, including integrative genetical genomics for understanding the complex quantitative trait of seed weight in chickpea.

Table S1. Chickpea genotypes used in the study for evaluating the amplification and polymorphic potential of CNMS markers among 25 chickpea genotypes.

Table S2. Characteristics of 666 CNMS markers developed from the non-coding upstream regulatory sequence components of 603 protein-coding genes of chickpea.

Table S3. Genomic distribution of physically mapped CNMS marker-associated genes on eight chickpea chromosomes.

Table S4. CNMS marker-associated chickpea genes predicted to function as master regulatory transcription factors in plants.

Supplementary Data

## References

[CIT0001] Agarwal G Jhanwar S Priya P Singh VK Saxena MS Parida SK Garg R Tyagi AK Jain M 2012 Comparative analysis of *kabuli* chickpea transcriptome with *desi* and wild chickpea provides a rich resource for development of functional markers. PLoS One 7, e52443. 23300670 10.1371/journal.pone.0052443 PMC3531472

[CIT0002] Agarwal P Kapoor S Tyagi AK 2011 Transcription factors regulating the progression of monocot and dicot seed development. Bioessays 33, 189–202. 21319185 10.1002/bies.201000107

[CIT0003] Bao S Corke H Sun M 2002 Microsatellites in starch-synthesizing genes in relation to starch physicochemical properties in waxy rice (*Oryza sativa* L.). Theoretical and Applied Genetics 105, 898–905. 12582915 10.1007/s00122-002-1049-3

[CIT0004] Baxter L Jironkin A Hickman R 2012 Conserved noncoding sequences highlight shared components of regulatory networks in dicotyledonous plants. The Plant Cell 24, 3949–3965. 23110901 10.1105/tpc.112.103010 PMC3517229

[CIT0005] Bevilacqua A Fiorenza MT Mangia F 2000 A developmentally regulated GAGA box-binding factor and Sp1 are required for transcription of the hsp70.1 gene at the onset of mouse zygotic genome activation. Development 127, 1541–1551. 10704399 10.1242/dev.127.7.1541

[CIT0006] Bharadwaj C Srivastava R Chauhan SK Satyavathi CT Kumar J Faruqui A Yadav S Rizvi AH Kumar T 2011 Molecular diversity and phylogeny in geographical collection of chickpea (*Cicer* sp.) accessions. Journal of Genetics 90, e94–e100. 22232200

[CIT0007] Bharathan G Janssen BJ Kellogg EA Sinha N 1997 Did homeodomain proteins duplicate before the origin of angiosperms, fungi, and metazoa? Proceedings of the National Academy of Sciences, USA 94, 13749–13753 10.1073/pnas.94.25.13749 PMC28378 9391098

[CIT0008] Boerjan W Vuylsteke M 2009 Integrative genetical genomics in *Arabidopsis* . Nature Genetics 41, 144–145. 19174836 10.1038/ng0209-144

[CIT0009] Bolle C Herrmann RG Oelmuller R 1996 Different sequences for 5ʹ-untranslated leaders of nuclear genes for plastid proteins affect the expression of the beta-glucuronidase gene. Plant Molecular Biology 32, 861–868. 8980537 10.1007/BF00020483

[CIT0010] Busturia A Lloyd A Bejarano F Zavortink M Xin H Sakonju S 2001 The MCP silencer of the *Drosophila* Abd-B gene requires both pleiohomeotic and GAGA factor for the maintenance of repression. Development 128, 2163–2173. 11493537 10.1242/dev.128.11.2163

[CIT0011] Caicedo AL Williamson SH Hernandez RD 2007 Genome-wide patterns of nucleotide polymorphim in domesticated rice. PLoS Genetics 3, e163. 10.1371/journal.pgen.0030163 PMC1994709 17907810

[CIT0012] Chan RL Gago GM Palena CM Gonzalez DH 1998 Homeoboxes in plant development. Biochimica et Biophysica Acta 1442, 1–19. 9767075 10.1016/s0167-4781(98)00119-5

[CIT0013] Choudhary S Gaur R Gupta S 2012 EST-derived genic molecular markers: development and utilization for generating an advanced transcript map of chickpea. Theoretical and Applied Genetics 124, 1449–1462. 22301907 10.1007/s00122-012-1800-3

[CIT0014] Choudhary S Sethy NK Shokeen B Bhatia S 2009 Development of chickpea EST-SSR markers and analysis of allelic variation across related species. Theoretical and Applied Genetics 118, 591–608. 19020854 10.1007/s00122-008-0923-z

[CIT0015] Clark RM Wagler TN Quijada P Doebley J 2006 A distant upstream enhancer at the maize domestication gene *tb1* has pleiotropic effects on plant and inflorescent architecture. Nature Genetics 38, 594–597. 16642024 10.1038/ng1784

[CIT0016] Colinas J Birnbaum K Benfey PN 2002 Using cauliflower to find conserved non-coding regions in *Arabidopsis* . Plant Physiology 129, 451–454. 12068091 10.1104/pp.002501 PMC1540231

[CIT0017] Creux NM Ranik M Berger DK Mybrug AA 2008 Comparative analysis of orthologous cellulose synthase promoters from *Arabidopsis*, *Populus* and *Eucalyptus*: evidence of conserved regulatory elements in angiosperms. New Phytologist 179, 722–737. 18547376 10.1111/j.1469-8137.2008.02517.x

[CIT0018] Doss S Schadt EE Drake TA Lusis AJ 2005 *Cis*-acting expression quantitative trait loci in mice. Genome Research 15, 681–691. 15837804 10.1101/gr.3216905 PMC1088296

[CIT0019] Dresselhaus T Cordts S Heuer S Sauter M Lorz H Kranz E 1999 Novel ribosomal genes from maize are differentially expressed in the zygotic and somatic cell cycles. Molecular and General Genetics 261, 416–427. 10102378 10.1007/s004380050983

[CIT0020] Emilsson V Thorleifsson G Zhang B 2008 Genetics of gene expression and its effect on disease. Nature 452, 423–428. 18344981 10.1038/nature06758

[CIT0021] Freeling M Subramaniam S 2009 Conserved noncoding sequences (CNSs) in higher plants. Current Opinion in Plant Biology 12, 126–132. 19249238 10.1016/j.pbi.2009.01.005

[CIT0022] Fujimori S Washio T Higo K 2003 A novel feature of microsatellites in plants: a distribution gradient along the direction of transcription. FEBS Letters 554, 17–22. 14596907 10.1016/s0014-5793(03)01041-x

[CIT0023] Garcia-Fernàndez J 2005 The genesis and evolution of homeobox gene clusters. Nature Reviews Genetics 6, 881–892. 10.1038/nrg1723 16341069

[CIT0024] Garg R Patel RK Jhanwar S Priya P Bhattacharjee A Yadav G Bhatia S Chattopadhyay D Tyagi AK Jain M 2011a Gene discovery and tissue-specific transcriptome analysis in chickpea with massively parallel pyrosequencing and web resource development. Plant Physiology 156, 1661–1678. 21653784 10.1104/pp.111.178616 PMC3149962

[CIT0025] Garg R Patel RK Tyagi AK Jain M 2011b *De novo* assembly of chickpea transcriptome using short reads for gene discovery and marker identification. DNA Research 18, 53–63. 21217129 10.1093/dnares/dsq028 PMC3041503

[CIT0026] Garg R Sahoo A Tyagi AK Jain M 2010 Validation of internal control genes for quantitative gene expression studies in chickpea (*Cicer arietinum* L.). Biochemical and Biophysical Research Communications 396, 283–288. 20399753 10.1016/j.bbrc.2010.04.079

[CIT0027] Gaur R Sethy NK Choudhary S Shokeen B Gupta V Bhatia S 2011 Advancing the STMS genomic resources for defining new locations on the intra-specific genetic linkage map of chickpea (*Cicer arietinum* L.). BMC Genomics 12, 117. 21329497 10.1186/1471-2164-12-117 PMC3050819

[CIT0028] Gibson G Weir B 2005 The quantitative genetics of transcription. Trends in Genetics 21, 616–623. 16154229 10.1016/j.tig.2005.08.010

[CIT0029] Gujaria N Kumar A Dauthal P 2011 Development and use of genic molecular markers (GMMs) for construction of a transcript map of chickpea (*Cicer arietinum* L.). Theoretical and Applied Genetics 122, 1577–1589. 21384113 10.1007/s00122-011-1556-1 PMC3082040

[CIT0030] Guo H Moose SP 2003 Conserved non-coding sequences among cultivated cereal genomes identify candidate regulator sequence elements and patterns of promoter evolution. The Plant Cell 15, 1143–1158. 12724540 10.1105/tpc.010181 PMC153722

[CIT0031] Gupta PK Rustgi S 2004 Molecular markers from the transcribed/expressed region of the genome in higher plants. Functional and Integrative Genomics 4, 139–162. 15095058 10.1007/s10142-004-0107-0

[CIT0032] Hawker NP Bowman JL 2004 Roles for Class III HD-Zip and KANADI genes in *Arabidopsis* root development. Plant Physiology 135, 2261–2270. 15286295 10.1104/pp.104.040196 PMC520795

[CIT0033] Hiremath PJ Farmer A Cannon SB Woodward J Kudapa H Tuteja R Kumar A Bhanu Prakash A Mulaosmanovic B Gujaria N 2011 Large-scale transcriptome analysis in chickpea (*Cicer arietinum* L.), an orphan legume crop of the semi-arid tropics of Asia and Africa. Plant Biotechnology Journal 9, 922–931. 21615673 10.1111/j.1467-7652.2011.00625.x PMC3437486

[CIT0034] Holland PWH 2013 Evolution of homeobox genes. Wiley Interdisciplinary Reviews: Developmental Biology 2, 31–45. 23799629 10.1002/wdev.78

[CIT0035] Hubner N Wallace CA Zimdahl H 2005 Integrated transcriptional profiling and linkage analysis for identification of genes underlying disease. Nature Genetics 37, 243–253. 15711544 10.1038/ng1522

[CIT0036] Hulzink RJ de Groot PF Croes AF Quaedvlieg W Twell D Wullems GJ Van Herpen MM 2002 The 5ʹ-untranslated region of the ntp303 gene strongly enhances translation during pollen tube growth, but not during pollen maturation. Plant Physiology 129, 342–353. 12011364 10.1104/pp.001701 PMC155897

[CIT0037] Husbands A Bell EM Shuai B Smith HM Springer PS 2007 LATERAL ORGAN BOUNDARIES defines a new family of DNA-binding transcription factors and can interact with specific bHLH proteins. Nucleic Acids Research 35, 6663–6671. 17913740 10.1093/nar/gkm775 PMC2095788

[CIT0038] Iglesias AR Kindlund E Tammi M Wadelius C 2004 Some microsatellites may act as novel polymorphic *cis*-regulatory elements through transcription factor binding. Gene 341, 149–165. 15474298 10.1016/j.gene.2004.06.035

[CIT0039] Ilegems M Douet V Meylan-Bettex M Uyttewaal M Brand L Bowman JL Stieger PA 2010 Interplay of auxin, KANADI and Class III HD-ZIP transcription factors in vascular tissue formation. Development 137, 975–984. 20179097 10.1242/dev.047662

[CIT0040] Izhaki A Bowman JL 2007 KANADI and class III HD-Zip gene families regulate embryo patterning and modulate auxin flow during embryogenesis in Arabidopsis. The Plant Cell 19, 495–508. 17307928 10.1105/tpc.106.047472 PMC1867338

[CIT0041] Jain M Misra G Patel RK 2013 A draft genome sequence of the pulse crop chickpea (*Cicer arietinum* L.). The Plant Journal 74, 715–729. 23489434 10.1111/tpj.12173

[CIT0042] Jain M Tyagi AK Khurana JP 2008 Genome-wide identification, classification, evolutionary expansion and expression analyses of homeobox genes in rice. FEBS Journal 275, 2845–2861. 18430022 10.1111/j.1742-4658.2008.06424.x

[CIT0043] Jansen RC Nap JP 2001 Genetical genomics: the added value from segregation. Trends in Genetics 17, 388–391. 11418218 10.1016/s0168-9525(01)02310-1

[CIT0044] Jhanwar S Priya P Garg R Parida SK Tyagi AK Jain M 2012 Transcriptome sequencing of wild chickpea as a rich resource for marker development. Plant Biotechnology Journal 10, 690–702. 22672127 10.1111/j.1467-7652.2012.00712.x

[CIT0045] Joehanes R Nelson JC 2008 QGene 4.0, an extensible Java QTL-analysis platform. Bioinformatics 24, 2788–2789. 18940826 10.1093/bioinformatics/btn523

[CIT0046] Johanson U West J Lister C Michaels S Amasino R Dean C 2000 Molecular analysis of *FRIGIDA*, a major determinant of natural variation in *Arabidopsis* flowering time. Science 290, 344–347. 11030654 10.1126/science.290.5490.344

[CIT0047] Kappen C 2000 The homeodomain: an ancient evolutionary motif in animals and plants. Journal of Computational Chemistry 24, 95–103. 10.1016/s0097-8485(99)00049-2 10642882

[CIT0048] Kim TY Kim HU Lee SY 2010 Data integration and analysis of biological networks. Current Opinion in Plant Biology 21, 78–84. 10.1016/j.copbio.2010.01.003 20138751

[CIT0049] Kloosterman B Oortwijn M uitdeWilligen J America T de Vos R Visser RG Bachem CW 2010 From QTL to candidate gene: genetical genomics of simple and complex traits in potato using a pooling strategy. BMC Genomics 11, 158. 20210995 10.1186/1471-2164-11-158 PMC2843620

[CIT0050] Koch MA Haubold B Mitchell-Olds T 2000 Comparative evolutionary analysis of chalcone synthase and alcohol dehydrogenase loci in *Arabidopsis*, *Arabis*, and related genera (Brassicaceae). Molecular Biology and Evolution 17, 1483–1498. 11018155 10.1093/oxfordjournals.molbev.a026248

[CIT0051] Koch MA Weisshaar B Kroymann J Haubold B Mitchell-Olds T 2001 Comparative genomics and regulatory evolution: conservation and function of the Chs and Apetala3 promoters. Molecular Biology and Evolution 18, 1882–1891. 11557794 10.1093/oxfordjournals.molbev.a003729

[CIT0052] Kooiker M Airoldi CA Losa A Manzotti PS Finzi L Kater MM Colombo L 2005 BASIC PENTACYSTEINE1, a GA binding protein that induces conformational changes in the regulator region of the homeotic *Arabidopsis* gene SEEDSTICK. The Plant Cell 17, 722–729. 15722463 10.1105/tpc.104.030130 PMC1069694

[CIT0053] Krusche P Tiskin A 2010 New algorithms for efficient parallel string comparison. In: The 22nd ACM symposium. New York: ACM Press, 209–216.

[CIT0054] Kujur A Bajaj D Saxena MS Tripathi S Upadhyaya HD Gowda CLL Singh S Jain M Tyagi AK Parida SK 2013 Functionally relevant microsatellite markers from chickpea transcription factor genes for efficient genotyping applications and trait association mapping. DNA Research 20, 355–373. 23633531 10.1093/dnares/dst015 PMC3738162

[CIT0055] Kujur A Bajaj D Saxena MS Tripathi S Upadhyaya HD Gowda CLL Singh S Tyagi AK Jain M Parida SK 2014 An efficient and cost-effective approach for genic microsatellite marker-based large-scale trait association mapping: identification of candidate genes for seed weight in chickpea. Molecular Breeding 34, 241–265.

[CIT0056] Lawson MJ Zhang L 2006 Distinct patterns of SSR distribution in the *Arabidopsis thaliana* and rice genomes. Genome Biology 7, R14. 16507170 10.1186/gb-2006-7-2-r14 PMC1431726

[CIT0057] Le BH Cheng C Bui AQ 2010 Global analysis of gene activity during *Arabidopsis* seed development and identification of seed-specific transcription factors. Proceedings of the National Academy of Sciences, USA 107, 8063–8070. 10.1073/pnas.1003530107 PMC2889569 20385809

[CIT0058] Levy S Hannennalli S Workman C 2001 Enrichment of regulatory signals in the conserved non-coding genomic sequences. Bioinformatics 17, 871–877. 11673231 10.1093/bioinformatics/17.10.871

[CIT0059] Liu K Muse SV 2005 PowerMarker: an integrated analysis environment for genetic marker analysis. Bioinformatics 21, 2128–2129. 15705655 10.1093/bioinformatics/bti282

[CIT0060] Loots GG Ovcharenko I Pachter L Dubchak I Rubin EM 2002 rVista for comparative sequence-based discovery of functional transcription factor binding sites. Genome Research 12, 832–839. 11997350 10.1101/gr.225502 PMC186580

[CIT0061] Maere S Heymans K Kuiper M 2005 BiNGO: a Cytoscape plugin to assess overrepresentation of gene ontology categories in biological networks. Bioinformatics 21, 3448–3449. 15972284 10.1093/bioinformatics/bti551

[CIT0062] Majer C Hochholdinger F 2011 Defining the boundaries: structure and function of LOB domain proteins. Trends in Plant Science 16, 47–52. 20961800 10.1016/j.tplants.2010.09.009

[CIT0063] Martin P Makepeace K Hill SA Hood DW Moxon ER 2004 Microsatellite instability regulates transcription factor binding and gene expression. Proceedings of the National Academy of Sciences, USA 102, 3800–3804. 10.1073/pnas.0406805102 PMC553301 15728391

[CIT0064] Meister RJ Williams LA Monfared MM Gallagher TL Kraft EA Nelson CG Gasser CS 2004 Definition and interactions of a positive regulatory element of the *Arabidopsis* INNER NO OUTER promoter. The Plant Journal 37, 426–438. 14731261 10.1046/j.1365-313x.2003.01971.x

[CIT0065] Morishige DT Childs KL Moore LD Mullet JE 2002 Targeted analysis of orthologous phytochrome A regions of the sorghum, maize, and rice genomes using comparative gene-island sequencing. Plant Physiology 130, 1614–1625. 12481045 10.1104/pp.012567 PMC166677

[CIT0066] Mukherjee K Brocchieri L Bürglin TR 2009 A comprehensive classification and evolutionary analysis of plant homeobox genes. Molecular Biology and Evolution 26, 2775–2794. 19734295 10.1093/molbev/msp201 PMC2775110

[CIT0067] Muller C Leutz A 2001 Chromatin remodeling in development and differentiation. Current Opinion in Genetics and Development 11, 167–174. 11250140 10.1016/s0959-437x(00)00175-1

[CIT0068] Nayak SN Zhu H Varghese N 2010 Integration of novel SSR and gene-based SNP marker loci in the chickpea genetic map and establishment of new anchor points with *Medicago truncatula* genome. Theoretical and Applied Genetics 120, 1415–1441. 20098978 10.1007/s00122-010-1265-1 PMC2854349

[CIT0069] Parida SK Dalal V Singh AK Singh NK Mohapatra T 2009 Genic non-coding microsatellites in the rice genome: characterization, marker design and use in assessing genetic and evolutionary relationships among domesticated groups. BMC Genomics 10, 140. 19335879 10.1186/1471-2164-10-140 PMC2680414

[CIT0070] Parida SK Pandit A Gaikwad K Sharma TR Srivastava PS Singh NK Mohapatra T 2010a Functionally relevant microsatellites in sugarcane unigenes. BMC Plant Biology 10, 251. 21083898 10.1186/1471-2229-10-251 PMC3017843

[CIT0071] Parida SK Yadava DK Mohapatra T 2010b Microsatellites in *Brassica* unigenes: relative abundance, marker design and use in comparative physical mapping and genome analysis. Genome 53, 55–67. 20130749 10.1139/g09-084

[CIT0072] Pauli S Rothnie HM Chen G He X Hohn T 2004 The cauliflower mosaic virus 35S promoter extends into the transcribed region. Journal of Virology 78, 12120–12128. 15507598 10.1128/JVI.78.22.12120-12128.2004 PMC525061

[CIT0073] Potokina E Prasad M Malysheva L Röder MS Graner A 2006 Expression genetics and haplotype analysis reveal *cis* regulation of serine carboxypeptidase I (*Cxp1*), a candidate gene for malting quality in barley (*Hordeum vulgare* L.). Functional and Integrative Genomics 6, 25–35. 16283224 10.1007/s10142-005-0008-x

[CIT0074] Reineke AR Bornberg-Bauer E Gu J 2011 Evolutionary divergence and limits of conserved non-coding sequence detection in plant genomes. Nucleic Acids Research 39, 6029–6043. 21470961 10.1093/nar/gkr179 PMC3152334

[CIT0075] Salvi S Sponza G Morgante M 2007 Conserved noncoding genomic sequences associated with a flowering-time quantitative trait locus in maize. Proceedings of the National Academy of Sciences, USA 104, 11376–11381. 10.1073/pnas.0704145104 PMC2040906 17595297

[CIT0076] Sangwan I O’Brian MR 2002 Identification of a soybean protein that interacts with GAGA element dinucleotide repeat DNA. Plant Physiology 129, 1788–1794. 12177492 10.1104/pp.002618 PMC166767

[CIT0077] Santi L Wang Y Stile MR Berendzen K Wanke D Roig C Pozzi C Muller K Muller J Rohde W 2003 The GA octodinucleotide repeat binding factor BBR participates in the transcriptional regulation of the homeobox gene Bkn3. The Plant Journal 34, 813–826. 12795701 10.1046/j.1365-313x.2003.01767.x

[CIT0078] Schadt EE Monks SA Drake TA 2003 Genetics of gene expression surveyed in maize, mouse and man. Nature 422, 297–302. 12646919 10.1038/nature01434

[CIT0079] Sethy NK Shokeen B Edwards KJ Bhatia S 2006 Development of microsatellite markers and analysis of intra-specific genetic variability in chickpea (*Cicer arietinum* L.). Theoretical and Applied Genetics 112, 1416–1428. 16534564 10.1007/s00122-006-0243-0

[CIT0080] Shuai B Reynaga-Peña CG Springer PS 2002 The lateral organ boundaries gene defines a novel, plant-specific gene family. Plant Physiology 129, 747–761. 12068116 10.1104/pp.010926 PMC161698

[CIT0081] Singh NK Gupta DK Jayaswal PK 2012 The first draft of the pigeonpea genome sequence. Journal of Plant Biochemistry and Biotechnology 21, 98–112. 24431589 10.1007/s13562-011-0088-8 PMC3886394

[CIT0082] Spensley M Kim JY Picot E Reid J Ott S Helliwell C Carré IA 2009 Evolutionarily conserved regulatory motifs in the promoter of the *Arabidopsis* clock gene LATE ELONGATED HYPOCOTYL. The Plant Cell 21, 2606–2623. 19789276 10.1105/tpc.109.069898 PMC2768935

[CIT0083] Svistoonoff S Creff A Reymond M 2007 Root tip contact with low-phosphate media reprograms plant root architecture. Nature Genetics 39, 792–796. 17496893 10.1038/ng2041

[CIT0084] Tagle DA Koop BF Goodman F Slightom JL Hess DL Jones RT 1988 Embryonic epsilon and gamma globin genes of a prosimian primate (*Galago crassicaudatus*): nucleotide and amino acid sequences, developmental regulation and phylogenetic foot-printing. Journal of Molecular Biology 20, 439–455. 3199442 10.1016/0022-2836(88)90011-3

[CIT0085] Terpstra IR Snoek LB Keurentjes JJ Peeters AJ van den Ackerveken G 2010 Regulatory network identification by genetical genomics: signaling downstream of the *Arabidopsis* receptor-like kinase ERECTA. Plant Physiology 154, 1067–1078. 20833726 10.1104/pp.110.159996 PMC2971588

[CIT0086] Upadhyaya HD Dwivedi SL Baum M Varshney RK Udupa SM Gowda CL Hoisington D Singh S 2008 Genetic structure, diversity, and allelic richness in composite collection and reference set in chickpea (*Cicer arietinum* L.). BMC Plant Biology 8, 106. 18922189 10.1186/1471-2229-8-106 PMC2583987

[CIT0087] Van Ooijen JW 2009 *MapQTL 6: software for the mapping of quantitative trait loci in experimental populations of diploid species*. Wageningen, The Netherlands: Kyazma BV.

[CIT0088] Varshney RK Chen W Li Y 2012 Draft genome sequence of pigeonpea (*Cajanus cajan*), an orphan legume crop of resource-poor farmers. Nature Biotechnology 30, 83–89. 10.1038/nbt.2022 22057054

[CIT0089] Varshney RK Song C Saxena RK 2013 Draft genome sequence of chickpea (*Cicer arietinum*) provides a resource for trait improvement. Nature Biotechnology 31, 240–246. 10.1038/nbt.2491 23354103

[CIT0090] Werner JD Borevitz JO Warthmann N Trainer GT Ecker JR Chory J Weigel D 2005 Quantitative trait locus mapping and DNA array hybridization identify an FLM deletion as a cause for natural flowering-time variation. Proceedings of the National Academy of Sciences, USA 102, 2460–2465. 10.1073/pnas.0409474102 PMC548991 15695584

[CIT0091] Wingender E Chen X Hehl R Karas H Liebich I Matys V Meinhardt T Prüss M Reuter I Schacherer F 2000 TRANSFAC: an integrated system for gene expression regulation. Nucleic Acids Research 28, 316–319. 10592259 10.1093/nar/28.1.316 PMC102445

[CIT0092] Winter P Benko-Iseppon AM Hüttel B 2000 A linkage map of chickpea (*Cicer arietinum* L.) genome based on recombinant inbred lines from a *C. arietinum×C. reticulatum* cross: localization of resistance genes for fusarium wilt races 4 and 5. Theoretical and Applied Genetics 101, 1155–1163.

[CIT0093] Yang X Tuskan GA Cheng MZ 2006 Divergence of the *Dof* gene families in poplar, *Arabidopsis*, and rice suggests multiple modes of gene evolution after duplication. Plant Physiology 142, 820–830. 16980566 10.1104/pp.106.083642 PMC1630746

[CIT0094] Zhang L Zuo K Zhang F Cao Y Wang J Zhang Y Sun X Tang K 2006 Conservation of noncoding microsatellites in plants: implication for gene regulation. BMC Genomics 7, 323. 17187690 10.1186/1471-2164-7-323 PMC1781443

[CIT0095] Zheng Y Ren N Wang H Stromberg AJ Perry SE 2009 Global identification of targets of the *Arabidopsis* MADS domain protein AGAMOUS-Like15. The Plant Cell 21, 2563–2577. 19767455 10.1105/tpc.109.068890 PMC2768919

