# Supplementary material for: Genome-wide conserved non-coding microsatellite (CNMS) marker-based integrative genetical genomics for quantitative dissection of seed weight in chickpea
Source: J Exp Bot. 2014 Dec 10;66(5):1271–90. doi: 10.1093/jxb/eru478 (PMC4339591; doi:10.1093/jxb/eru478)
Supplement: Supplementary Data [file supp_66_5_1271__index.html]

Genome-wide conserved non-coding microsatellite (CNMS) marker-based integrative genetical genomics for quantitative dissection of seed weight in chickpea — Genome-wide conserved non-coding microsatellite (CNMS) marker-based integrative genetical genomics for quantitative dissection of seed weight in chickpea — Supplementary Data 

# Genome-wide conserved non-coding microsatellite (CNMS) marker-based integrative genetical genomics for quantitative dissection of seed weight in chickpea

## Supplementary Data

Data files

**Files in this Data Supplement:**

- Supplementary Data - Supplementary Data
