# Supplementary material for: Genome-wide conserved non-coding microsatellite (CNMS) marker-based integrative genetical genomics for quantitative dissection of seed weight in chickpea
Source: J Exp Bot. 2014 Dec 10;66(5):1271–90. doi: 10.1093/jxb/eru478 (PMC4339591; doi:10.1093/jxb/eru478)
Supplement: Supplementary Data [file supp_eru478_jexbot128363_file001.pdf]

Genome-wide conserved non-coding microsatellite (CNMS) marker-based integrative genetical genomics for quantitative dissection of seed weight in chickpea

Deepak Bajaj, Maneesha S. Saxena, Alice Kujur, Shouvik Das, Saurabh Badoni, Shailesh Tripathi, Hari D. Upadhyaya, C.L.L. Gowda, Shivali Sharma, Sube Singh, Akhilesh K. Tyagi, Swarup K. Parida

Supporting Information

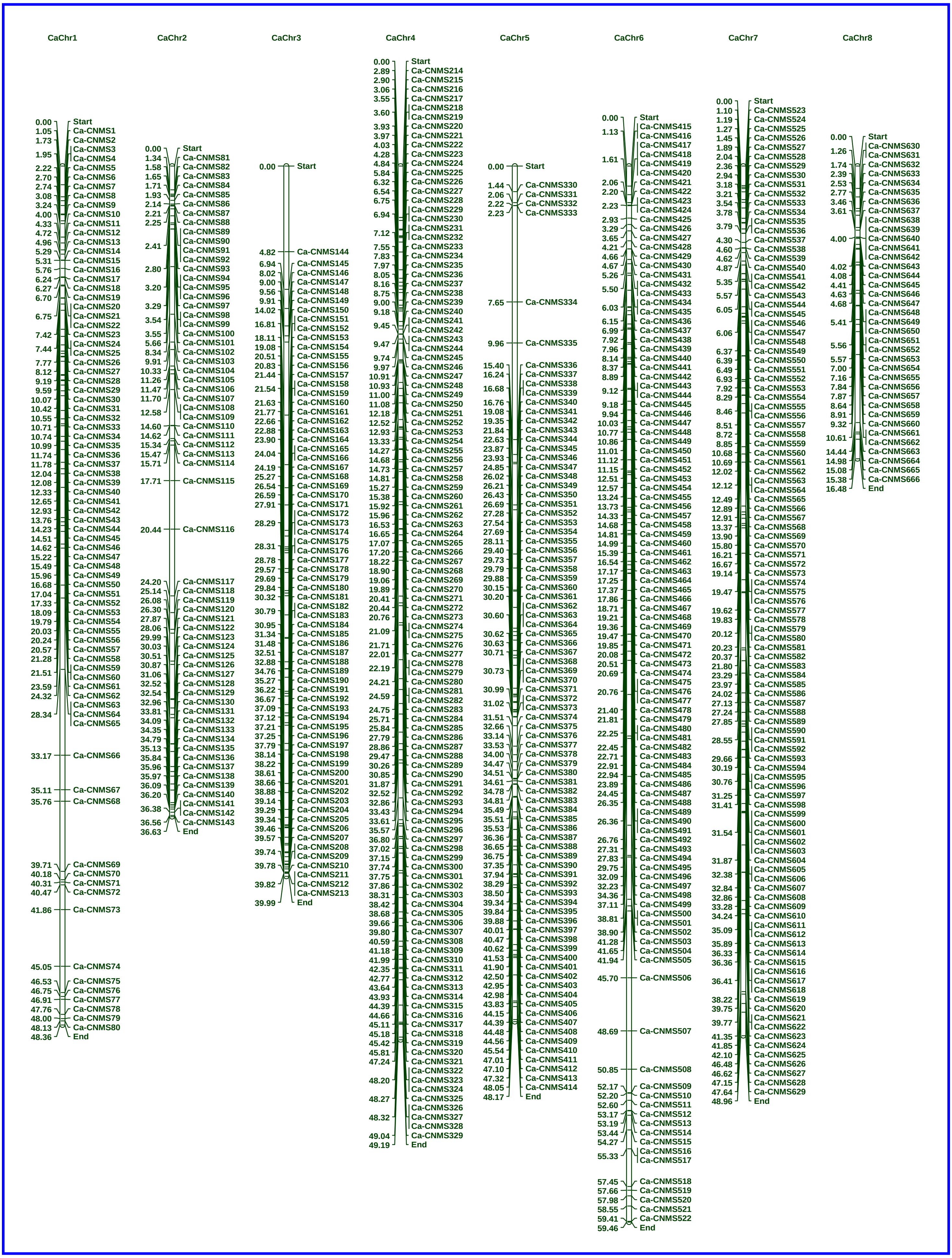

Figure S1: A physical map of the chickpea constructed using 666 CNMS marker-associated genes with an average density of 521.4 kb. The physical distance (Mb) and identity of the marker loci integrated on the eight chromosomes are indicated on the left and right sides of the chromosomes, respectively. The identity of the markers mentioned on the right side of chromosomes correspond with the marker IDs “CNMS” as provided in Table S2. The sizes of the chromosomes (Mb) are based on the recently released draft genome sequence (Pseudomolecule V1.1) of the chickpea (Varshney *et al.*, 2013).

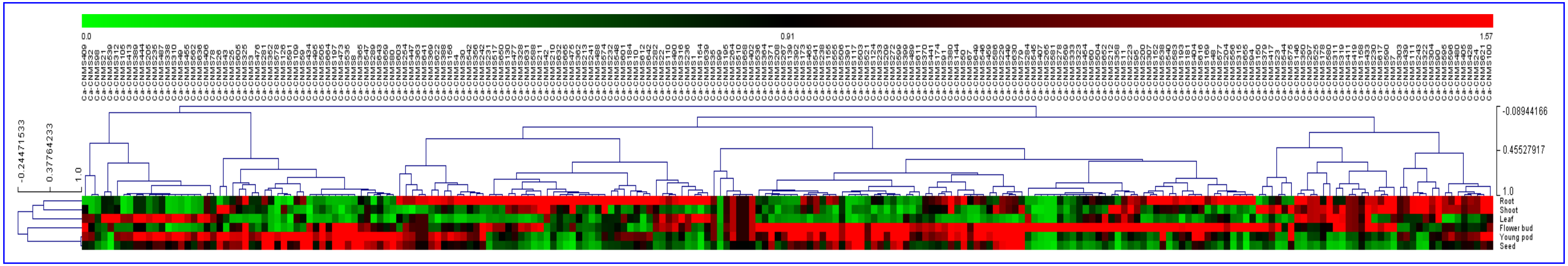

Figure S2: Hierarchical cluster display representing the expression profile of 220 CNMS marker-associated genes that were differentially expressed in six different vegetative and reproductive tissues of the chickpea genotype ICC 4958. The colour scale at the top represents the average log of the signal expression values of the genes in the various tissues, in which green, black and red colours signify low, medium and high expression levels, respectively. The tissues and CNMS marker-associated genes that were used for the expression profiling are mentioned on the right and top sides of expression map, respectively.

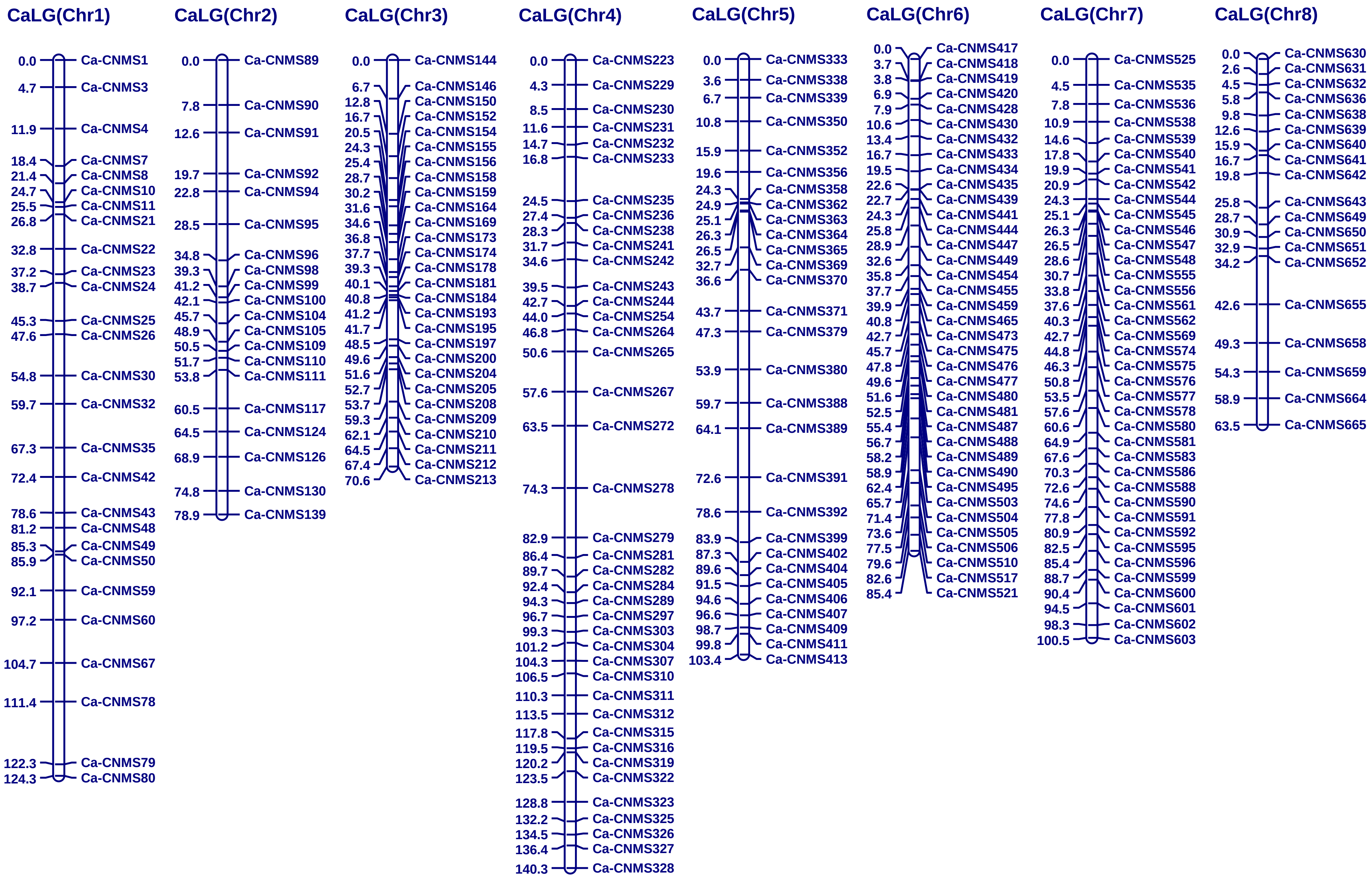

Figure S3: A functional transcript map (genetic linkage map) of the chickpea (ICC 4958 x ICC 17163) constructed using 238 parental polymorphic CNMS marker-associated genes. The genetic distances (cM) and identities of the marker loci integrated on the eight LGs/chromosomes are indicated on the left and right sides of the chromosomes, respectively.

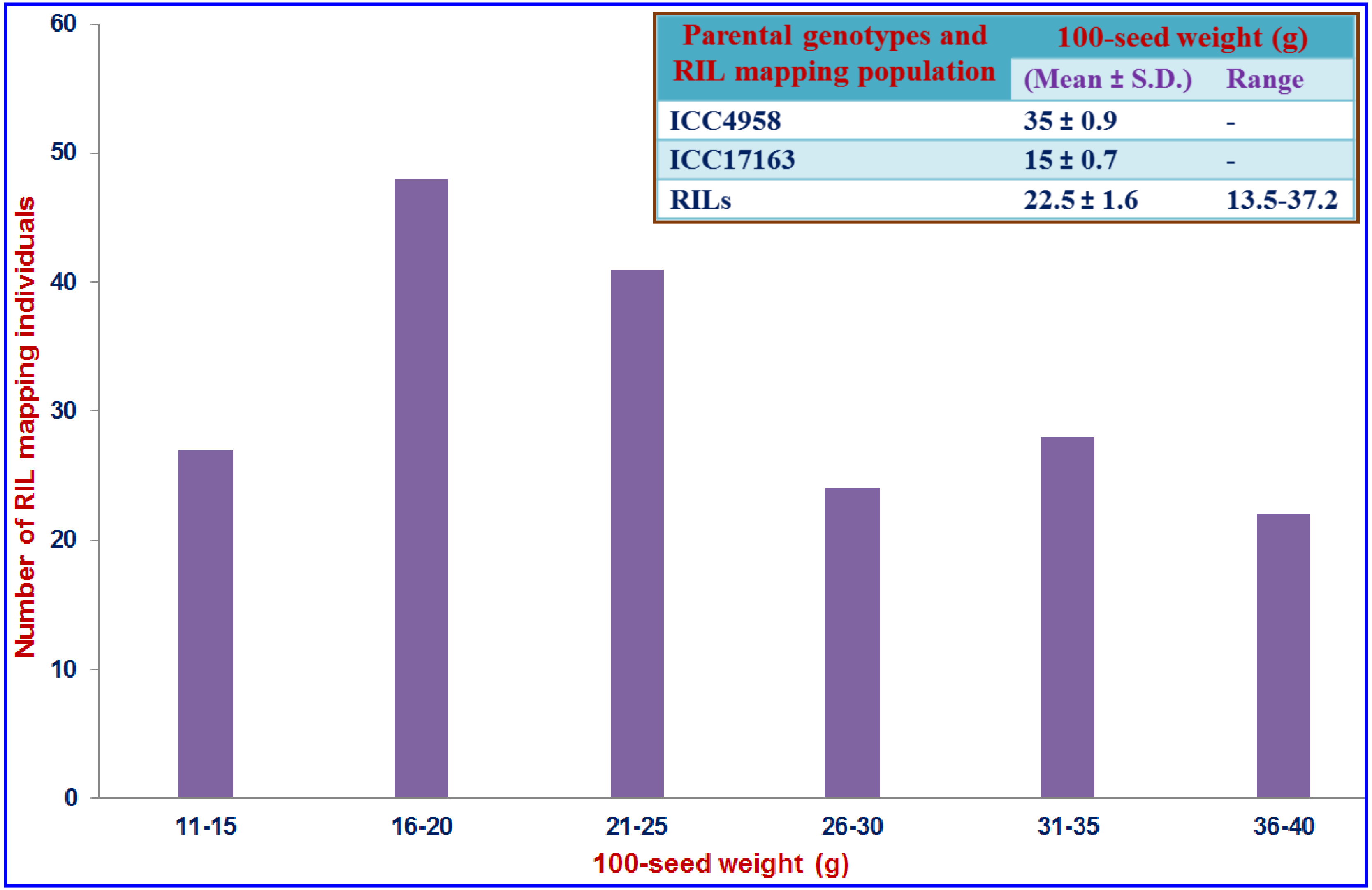

Figure S4: The frequency distribution of 100-seed weight (g) among 190 individuals of a RIL mapping population (ICC 4958 x ICC 17163) revealed the significant variation of seed weight trait between parental genotypes and among mapping individuals, and depicted a goodness-of-fit to the normal distribution.

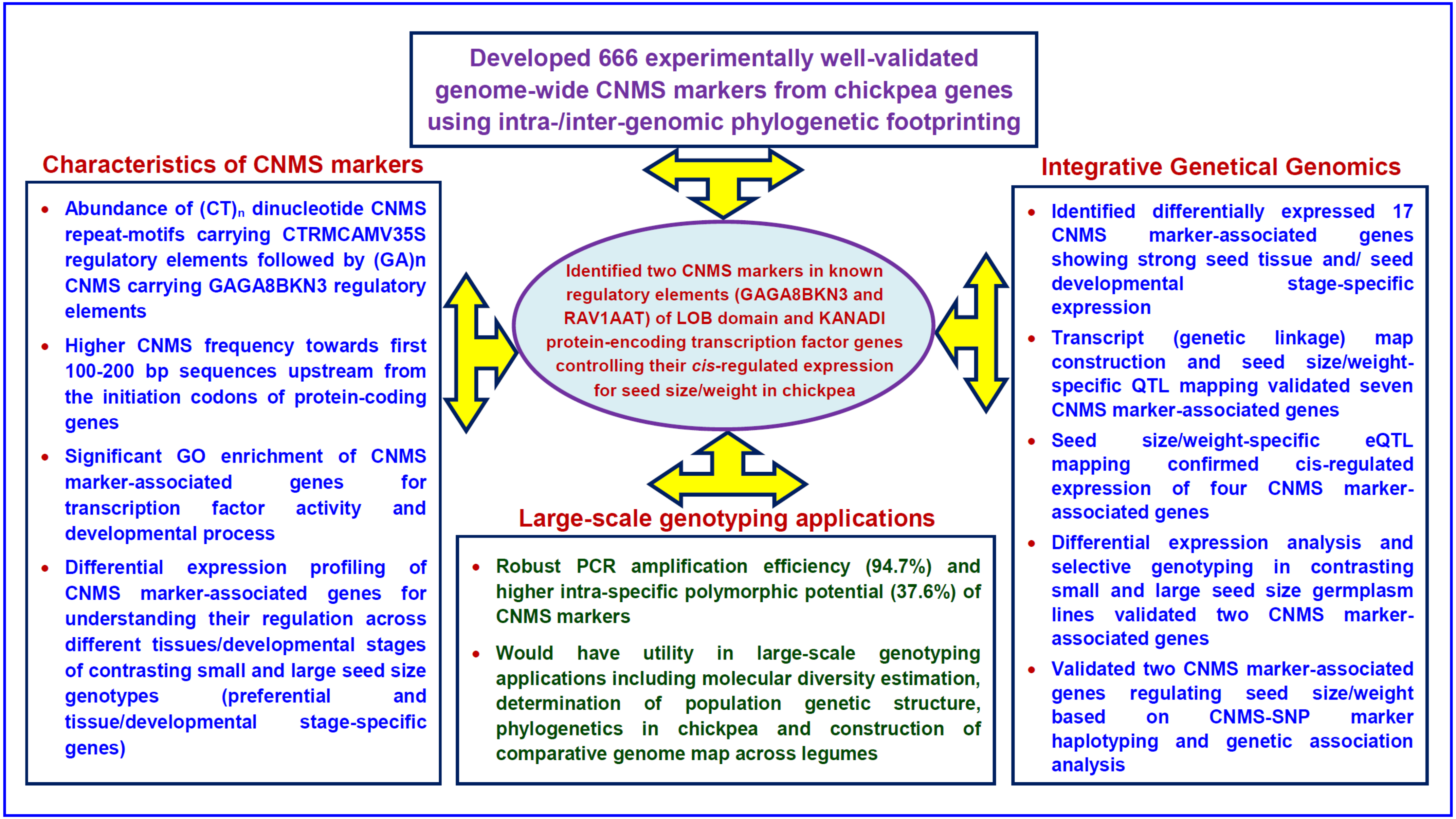

Figure S5: Characteristics, functional significance and utility of genome-wide CNMS marker-associated genes for large-scale genotyping applications, including integrative genetical genomics for understanding the complex quantitative trait of seed weight in chickpea.

Table S1: Chickpea genotypes used in the study for evaluating the amplification and polymorphic potential of CNMS markers among 25 chickpea genotypes

Sl. No.	Accession IDs	Cultivar types	Geographical origin
1	ICCV2	*Cicer arietinum Kabuli* variety	South India
2	ICCV95332	*Cicer arietinum Kabuli* variety	Central India
3	ICCV92311	*Cicer arietinum Kabuli* variety	Central India
4	ICCV92337	*Cicer arietinum Kabuli* variety	Central India
5	ICCV95311	*Cicer arietinum Kabuli* variety	South India
6	ICCV96329	*Cicer arietinum Kabuli* variety	South India
7	ICCV95334	*Cicer arietinum Kabuli* variety	Central India
8	ICCX810800	*Cicer arietinum Desi* variety	South India
9	IC296132	*Cicer arietinum Desi* variety	North India
10	ICCV1	*Cicer arietinum Desi* variety	Central India
11	ICCV89314	*Cicer arietinum Desi* variety	East India
12	BGD112	*Cicer arietinum Desi* landrace	North India
13	ICCV96970	*Cicer arietinum Desi* variety	Central India
14	ICCC37	*Cicer arietinum Desi* variety	South India
15	ICCV94954	*Cicer arietinum Desi* variety	Central India
16	ICCV10	*Cicer arietinum Desi* variety	South/Central India
17	ICCV88202	*Cicer arietinum Desi* variety	North India
18	ICCV93952	*Cicer arietinum Desi* variety	Central India
19	ICCV93954	*Cicer arietinum Desi* variety	South India
20	IC296133	*Cicer arietinum Desi* variety	North India
21	ICCV929944	*Cicer arietinum Desi* variety	Central India
22	ICCV94954	*Cicer arietinum Desi* variety	Central India
23	IC296131	*Cicer arietinum Desi* variety	North India
24	ICC4958	*Cicer arietinum Desi* landrace	Central India
25	PI489777	*Cicer reticulatum* Wild	Turkey

Table S2: Characteristics of 666 CNMS markers developed from the non-coding upstream regulatory sequence components of 603 protein coding genes of chickpea

Marker IDs	NCBI Probe IDs (PUIDs)	Chromosomes	Physical positions (bp)	Microsatellite repeat-motifs	Forward Primer (5'-3')	Reverse Primer (5'-3')	Actual annealing tempearture (0C)	Size (bp) of alleles amplified	Regulatory elements	Signal sequences of known regulatory element-binding sites	Function of known regulatory elements/ transcription factor-binding sites	Putative gene function
Ca-CNMS1	28366769	CaChr1	1050855	(AAG)5	CATCCAAGACAAAATTCCTTA	CTCTGGAAATTTAAACAAGCA	55.0	158	UPRMOTIFIIAT	CC(N)10CCACG	Motif II in conseved UPR element in SAR1B, HSP90, Ca-Atase etc genes	Paralog of NPR1
Ca-CNMS2	28366880	CaChr1	1726298	(CT)8	CAAGACACTCACTCACTCACACC	TGTTGTGTTTGTCTTGGGGA	59.9	276	CTRMCAMV35S	TCTCTCTCT	CaMV 35S (Inverted GAGA) found in a 60-nucleotide region downstream of the transcription start site of the	NADPH-dependent FMN reductase
Ca-CNMS3	28366991	CaChr1	1953960	(CT)8	GCCATTCTTCACTCTTTCTTT	GTTCAAATCCGAAACGTAAAT	55.0	148	CTRMCAMV35S	TCTCTCTCT	CaMV 35S (Inverted GAGA) found in a 60-nucleotide region downstream of the transcription start site of the	HSI2-like 1 (HSL1)
Ca-CNMS4	28367102	CaChr1	1953960	(TGG)5	GTTGTTTCTCCCATTTTTCTT	CATTCTCATCATTCTCTTCCA	55.0	157	PALBOXPPC	YTYYMMCMAMCMMC	Putative cis-acting elements of phenylalanine ammonia-lyase genes in parsley	TCP family transcription factor
Ca-CNMS5	28367213	CaChr1	2219563	(TCG)5	ATTCTCAACGGATCCAGCAC	TGGCATGCAATTTCCAACTA	60.1	262	CGACGOSAMY3	CGACG	Amy 3D & Amy 3E alpha-amylase gene	Tyrosinase
Ca-CNMS6	28367324	CaChr1	2701788	(TTG)5	TGTTGTTTGCGTTCATTCCT	TGGTTGAAAAACAGAACAACG	59.2	246	CAREOSREP1	CAACTC	Motif found in REP-1 gene promoter	Pathogenesis-related transcriptional factor/ERF, DNA-
Ca-CNMS7	28367402	CaChr1	2735621	(AATC)3	AGAGTTGCAATGTTGTTGAGT	ACGAAGAGAGTGTTAGGGTTT	55.0	145	CAATBOX1	CAAT	CAAT promoter consensus sequence" found in legA gene of pea	Mitochondrial transcription termination factor family protein
Ca-CNMS8	28367413	CaChr1	3077603	(AGTTG)3	GCTTTCCTTTCTTATGACCAC	CATGAGGACAATGAAGTCAATA	56.0	150	CAREOSREP1	CAACTC	Motif found in REP-1 gene promoter	DNA-binding bromodomain-containing protein
Ca-CNMS9	28367424	CaChr1	3235246	(GCA)5	TTCACTTCCCCTCCCTTCTT	CGGCGATGGATAATGGTTAC	60.0	176	ANAERO2CONSENSUS	AGCAGC	Motif found in silico in promoters of 13 anaerobic genes involved in the fermentative pathway	Domain of unknown function DUF640
Ca-CNMS10	28366770	CaChr1	3999120	(TTCT)4	AGAGAGTGACGTGTCCTTAGA	CTGAGAGTTCTTCTCCTCGAT	54.0	151	CYTOSITECSHPRA	AAGATTGATTGAG	Motif found in cucumber (C.s.) hydroxypyruvate reductase (hprA) gene promoter	CCCH-type zinc finger protein with ARM repeat domain
Ca-CNMS11	28366781	CaChr1	4325564	(TTC)4	GTGGAATTTCATATGGGTTTA	TCCACTCAGAGATACTCTCCA	54.0	153	POLLEN1LELAT52	AGAAA	One of two co-dependent regulatory elements responsible for pollen specific activation of tomato lat52	Duplicated homeodomain-like superfamily protein
Ca-CNMS12	28366792	CaChr1	4719174	(GA)11	TCAAAGAACCAAAATTCGAACA	CAAAACGGTGCACTTTGAGA	59.6	219	GAGA8HVBKN3	GAGAGAGAGAGAGAGA	Motif found in intron IV of the barley (H.v.) gene Bkn3	CMP/dCMP deaminase, zinc-binding
Ca-CNMS13	28366803	CaChr1	4955275	(AAAC)5	TCCTACTGGCAATTTGCATTT	GCTGAGAAAGCTAAGGTCCG	59.6	239	AACACOREOSGLUB1	AACAAAC	Motif found in rice (O.s.) glutelin genes, involved in controlling the endosperm-specific expression	Guanylate kinase
Ca-CNMS14	28366814	CaChr1	5289320	(TTG)5	TTCCACCCTTTTGACCTTTG	TGTGGACCCAAATGTTCTCA	59.9	170	CAREOSREP1	CAACTC	Motif found in REP-1 gene promoter	Heavy metal transport/detoxification protein
Ca-CNMS15	28366825	CaChr1	5308048	(TTG)7	GGTCAAGGCGCAAAGTATGT	CGACTTCAATACCATCCTTACTATCC	60.1	166	CAREOSREP1	CAACTC	Motif found in REP-1 gene promoter	Zinc finger, RING-type
Ca-CNMS16	28366836	CaChr1	5764643	(TTG)5	AAGCTTCCAAGAGACGGTGA	TCCTGGTTTAGGTTACTATTTTATGGA	60.0	128	CAREOSREP1	CAACTC	Motif found in REP-1 gene promoter	Homeobox
Ca-CNMS17	28366847	CaChr1	6242296	(AG)6	TCCTCCAAAAGTGGAACCAA	CATGGAATTGGGAGCTGATT	60.5	168	CTRMCAMV35S	TCTCTCTCT	CaMV 35S (Inverted GAGA) found in a 60-nucleotide region downstream of the transcription start site of the	Dehydroquinase class I
Ca-CNMS18	28366858	CaChr1	6266048	(ACT)5	CCTGATGATTCCAACCTTGC	CAGTTCTCAATGCTGCTCAAA	60.5	270	CACTFTPPCA1	YACT	Tetranucleotide (CACT) is a key component of Mem1 found in the cis-regulatory element in the distal region of	Transcription factor GRAS
Ca-CNMS19	28366869	CaChr1	6703208	(TC)18	TCCAACACACCCACAAGAAA	TTCAGCTTTGGACAAAAGGG	60.0	238	CTRMCAMV35S	TCTCTCTCT	CaMV 35S (Inverted GAGA) found in a 60-nucleotide region downstream of the transcription start site of the	Glycosyl transferase, family 8
Ca-CNMS20	28366881	CaChr1	6750424	(TC)16	CGAAATAGAAGGGAGCGAAA	AAAATGCTAAAAGGGTCGCC	59.4	194	CTRMCAMV35S	TCTCTCTCT	CaMV 35S (Inverted GAGA) found in a 60-nucleotide region downstream of the transcription start site of the	Myb, DNA-binding
Ca-CNMS21	28366892	CaChr1	6751424	(TC)8	TACCCAGATGAGATCTACGAA	GGATGGTATCACTGTTTGTGT	55.0	152	CTRMCAMV35S	TCTCTCTCT	CaMV 35S (Inverted GAGA) found in a 60-nucleotide region downstream of the transcription start site of the	Myb-like HTH transcriptional regulator family protein
Ca-CNMS22	28366903	CaChr1	6751424	(TCT)6	TACCCAGATGAGATCTACGAA	GGATGGTATCACTGTTTGTGT	55.0	152	CYTOSITECSHPRA	AAGATTGATTGAG	Involved in light responsiveness	Myb-like HTH transcriptional regulator family protein
Ca-CNMS23	28366914	CaChr1	7416773	(TCT)4	GCTGTTTGTTTGATGAATCTC	GGATAGTGAAGGCTCTCTTGT	55.0	159	CYTOSITECSHPRA	AAGATTGATTGAG	Required for cytokinin responsiveness	PHB
Ca-CNMS24	28366925	CaChr1	7435710	(ACA)4	AGAACACACAGAGACAGTGCT	AATTGATGTGTTGTTGTGGTT	55.0	148	RAV1AAT	CAACA	AP2 & B3 like domiane in RAV1	Nuclear factor Y, subunit A7 (NF-YA7)
Ca-CNMS25	28366936	CaChr1	7435710	(CTCTT)4	CAACACTTTCCCTTCACTTC	AGAACAAGCTCTTGAGGAAGT	55.0	153	NODCON2GM	CTCTT	One of two putative nodulin consensus sequences	Nuclear factor Y, subunit A7 (NF-YA7)
Ca-CNMS26	28366947	CaChr1	7771087	(AG)8	TGCAGAGAAAGAGTGAGAAAG	TGCTAACATCTTCAGTTCCTC	55.0	152	CTRMCAMV35S	TCTCTCTCT	CaMV 35S (Inverted GAGA) found in a 60-nucleotide region downstream of the transcription start site of the	TLP family
Ca-CNMS27	28366958	CaChr1	8118508	(TC)8	TACCCGAAGCAACATAAGGC	GAAACGGTGAAGCATTTGGT	60.1	155	CTRMCAMV35S	TCTCTCTCT	CaMV 35S (Inverted GAGA) found in a 60-nucleotide region downstream of the transcription start site of the	YTH domain
Ca-CNMS28	28366969	CaChr1	9188736	(TC)18	TCACTCACTCAACAAAGCGG	CCCACTTCACTGTAGCTCCC	60.0	268	CTRMCAMV35S	TCTCTCTCT	CaMV 35S (Inverted GAGA) found in a 60-nucleotide region downstream of the transcription start site of the	Peptidase S26B, eukaryotic signal peptidase
Ca-CNMS29	28366980	CaChr1	9590348	(TTTA)5	AAACCCTTTCCACAGTTTTTGA	GACGATGACCTTTTGGGAGA	59.9	199	TATABOX5	TTATTT	TATA box found in the 5'upstream region of pea glutamine synthetase gene	F-box domain, cyclin-like
Ca-CNMS30	28366992	CaChr1	10066267	(GT)6	AATAAAAGGTGTTTGGGGTAG	CCTTCATAGGTTGATTTGATG	55.0	143	CACTFTPPCA1	YACT	Tetranucleotide (CACT) is a key component of Mem1 found in the cis-regulatory element in the distal region of	Phytochrome-associated protein 1 (PAP1)
Ca-CNMS31	28367003	CaChr1	10418778	(CTA)8	TGAGTTGACACGCAGAATGA	CCCCTATGGCACAAAATCTC	59.0	163	CACTFTPPCA1	YACT	Tetranucleotide (CACT) is a key component of Mem1 found in the cis-regulatory element in the distal region of	3-Hydroxyisobutyrate dehydrogenase-related, conserved
Ca-CNMS32	28367014	CaChr1	10554319	(TC)6	AAACTGCGTCGTGTGTTT	TTATGGGTTTCGTCAAAGTTA	55.0	155	CTRMCAMV35S	TCTCTCTCT	CaMV 35S (Inverted GAGA) found in a 60-nucleotide region downstream of the transcription start site of the	AL3, Alfin-Like family; PhD domain containing homeodomain proteins
Ca-CNMS33	28367025	CaChr1	10709193	(TC)12	TGGAAGCTTCGGTTTTTCAT	CGGAAGATCGAAGCGTAAAA	59.7	128	CTRMCAMV35S	TCTCTCTCT	CaMV 35S (Inverted GAGA) found in a 60-nucleotide region downstream of the transcription start site of the	Basic-leucine zipper (bZIP) transcription factor
Ca-CNMS34	28367036	CaChr1	10739296	(CAAAAT)5	TCCATTTTTGAGGTCGGAAG	GCCCATTGTTAGATCTGGGA	60.0	186	ARR1AT	NGATT	Motif found in the promoter of rice NSHB gene	Ribokinase
Ca-CNMS35	28367047	CaChr1	10990150	(TGA)6	CTTTATCATGTTCACCAGAGG	TAATCTCTTCTTCCACCCTTT	55.0	156	CAATBOX1	CAAT	CAAT promoter consensus sequence found in legA gene of pea	A component of the PHYA signaling network
Ca-CNMS36	28367058	CaChr1	11740013	(TATT)7	TCGTAACGGCCTTCAAATTC	ACAAAAACACTGCACCACCA	60.1	233	TATABOX5	TTATTT	TATA box found in the 5'upstream region of pea glutamine synthetase gene	Kinesin, motor domain
Ca-CNMS37	28367069	CaChr1	11781665	(GA)7	AGCCCCAATTTGATTTGTTG	TGGTCAGTGAAACATTCTCATGTA	59.8	201	CTRMCAMV35S	TCTCTCTCT	CaMV 35S (Inverted GAGA) found in a 60-nucleotide region downstream of the transcription start site of the	Protein kinase, catalytic domain
Ca-CNMS38	28367080	CaChr1	12043491	(TTTA)5	TGCCAAAATATTAAATGCACAAC	TGACGACAAATATGCATTACAAA	59.0	151	TATABOX5	TTATTT	TATA box found in the 5'upstream region of pea glutamine synthetase gene	Zinc finger, RING-type
Ca-CNMS39	28367091	CaChr1	12079263	(AG)7	TGAGTAAGGCTTGGATGTCCT	TGTGATAAGCTCATTGCTTGAAA	58.8	216	CTRMCAMV35S	TCTCTCTCT	CaMV 35S (Inverted GAGA) found in a 60-nucleotide region downstream of the transcription start site of the	Cytokinin riboside 5'-monophosphate
Ca-CNMS40	28367103	CaChr1	12332559	(CT)9	TGTTTTTCCATCCATTTGTGA	TTTGTTACTGCTTTTTGTACTTGGA	58.9	226	CTRMCAMV35S	TCTCTCTCT	CaMV 35S (Inverted GAGA) found in a 60-nucleotide region downstream of the transcription start site of the	AUX/IAA protein
Ca-CNMS41	28367114	CaChr1	12654512	(GTG)5	GAGCACGTTATTTGGCCATT	CATGATGTGACCCATTTCAA	60.0	232	UPRMOTIFIIAT	CCNNNNNNNNNNNNCCACG	conserved UPR (unfolded protein response) cis-acting element in Arabidopsis	Frigida-like

Marker IDs	NCBI Probe IDs (PUIDs)	Chromosomes	Physical positions (bp)	Microsatellite repeat-motifs	Forward Primer (5'-3')	Reverse Primer (5'-3')	Actual annealing tempearture (0C)	Size (bp) of alleles amplified	Regulatory elements	Signal sequences of known regulatory element-binding sites	Function of known regulatory elements/ transcription factor-binding sites	Putative gene function
Ca-CNMS42	28367125	CaChr1	12929374	(CTTT)3	CCTTTGGTTGTAGATGGTGT	GGAACCAAACGTGAGTATAAA	55.0	141	POLLEN1LELAT52	AGAAA	One of two co-dependent regulatory elements responsible for pollen specific activation of tomato lat52	Putative protein with histone acetyl-transferase activity
Ca-CNMS43	28367136	CaChr1	13755312	(AGAA)3	AAATTGAACCTGAACAACGTA	ATCCTTACATTCCTCTGCTGT	55.0	148	POLLEN1LELAT52	AGAAA	One of two co-dependent regulatory elements responsible for pollen specific activation of tomato lat52	SET domain protein 2 (SDG2)
Ca-CNMS44	28367147	CaChr1	14226593	(GA)89	CCCTCTGGCCATAAATGAGA	CCGAGTCCAATTGCAACATT	60.0	246	GAGA8HVBKN3	GAGAGAGAGAGAGAGA	Motif found in intron IV of the barley (H.v.) gene Bkn3	DNA-binding WRKY
Ca-CNMS45	28367158	CaChr1	14505086	(CT)7	CCGAAAATTGAAAGGTGGAA	TTGTGCAGTAATTGCGTTGA	59.9	183	CTRMCAMV35S	TCTCTCTCT	CaMV 35S (Inverted GAGA) found in a 60-nucleotide region downstream of the transcription start site of the	Protein kinase, catalytic domain
Ca-CNMS46	28367169	CaChr1	14620725	(AGAT)6	GGACACATGTTTGGAAGCAA	TTCAATGAGCGATCCAAGTG	59.5	258	GATABOX	GATA	GATA motif in CaMV 35S promoter required for high level, light regulated, and tissue specific expression	Dcp1-like decapping
Ca-CNMS47	28367180	CaChr1	15220775	(AG)8	GAAGAAGCATCAAGGCAACA	TGGATTAGGAGATTTTGCTGCT	59.0	153	CTRMCAMV35S	TCTCTCTCT	CaMV 35S (Inverted GAGA) found in a 60-nucleotide region downstream of the transcription start site of the	Protein kinase, catalytic domain
Ca-CNMS48	28367191	CaChr1	15487424	(GAGTT)3	AATCCAATCGAAGAGAGAGAG	ACTCTAGGGTTTTCACTTTCC	55.0	154	CAREOSREP1	CAACTC	Found in REP-1 gene promoter	DNA binding
Ca-CNMS49	28367202	CaChr1	15963878	(AAAAGA)3	ATCTCTTTCTTCCCCACTAAA	CATCAAAGCGTAATCAAAAAG	55.0	195	POLLEN1LELAT52	AGAAA	Elements responsible for pollen specific activation of tomato lat52 gene	IAA-leucine resistant3 (ILR3)
Ca-CNMS50	28367214	CaChr1	16679244	(TC)6	CAGTGTCTGAATTTTCGTCAT	TTCTCCATTGTAGGAGTAGCA	55.0	158	CTRMCAMV35S	TCTCTCTCT	CaMV 35S (Inverted GAGA) found in a 60-nucleotide region downstream of the transcription start site of the	TEOSINTE BRANCHED 1, cycloidea and PCF transcription
Ca-CNMS51	28367225	CaChr1	17043720	(TC)9	GGCCTAAGCACAAACAGGAG	CAGACTTTACCACCACACATCAA	59.9	158	CTRMCAMV35S	TCTCTCTCT	CaMV 35S (Inverted GAGA) found in a 60-nucleotide region downstream of the transcription start site of the	Protein kinase, catalytic domain
Ca-CNMS52	28367236	CaChr1	17332510	(AAC)5	AAGAGTTGGATCCGCAGCTA	TGCAAGTTGGTGTTGAGAGG	60.0	208	RAV1AAT	CAACA	AP2 & B3 like domiane in RAV1	Zinc finger, TFIIS-type
Ca-CNMS53	28367247	CaChr1	18091915	(TC)9	CATCGGTTCGTTCAACTTCA	GCCTTCGTTTGCGAGAGATA	59.7	228	CTRMCAMV35S	TCTCTCTCT	CaMV 35S (Inverted GAGA) found in a 60-nucleotide region downstream of the transcription start site of the	Kinesin, motor domain
Ca-CNMS54	28367258	CaChr1	19790244	(TC)7	GTTGTTGGAAAATCGGATGG	TTAAATCGGAGGATTTGGGC	60.2	126	CTRMCAMV35S	TCTCTCTCT	CaMV 35S (Inverted GAGA) found in a 60-nucleotide region downstream of the transcription start site of the	Oxysterol-binding protein
Ca-CNMS55	28367269	CaChr1	20031195	(AAT)18	TGAATTTCATCTTGTGTTGCG	CCATTTACGTGACCCGACTC	59.7	218	CTRMCAMV35S	TCTCTCTCT	CaMV 35S (Inverted GAGA) found in a 60-nucleotide region downstream of the transcription start site of the	Homeobox
Ca-CNMS56	28367280	CaChr1	20243744	(TC)8	AAAACCCACTTGATTGCTGC	TGTTCATTTAAAGAGAGGAGAGAGC	60.1	122	CTRMCAMV35S	TCTCTCTCT	CaMV 35S (Inverted GAGA) found in a 60-nucleotide region downstream of the transcription start site of the	SANT domain, DNA binding
Ca-CNMS57	28367291	CaChr1	20565621	(CT)6	ATGCAACCTGCATTACCACA	AATGTGAAGGGTGTTGAGGG	60.0	190	CTRMCAMV35S	TCTCTCTCT	CaMV 35S (Inverted GAGA) found in a 60-nucleotide region downstream of the transcription start site of the	ATPase, AAA+ type, core
Ca-CNMS58	28367302	CaChr1	21279469	(TC)6	GGCAATAAGGAACTAGCAATGG	CGGTTGTGATTGATTAGCAGTG	60.0	257	CTRMCAMV35S	TCTCTCTCT	CaMV 35S (Inverted GAGA) found in a 60-nucleotide region downstream of the transcription start site of the	Sec1-like protein
Ca-CNMS59	28367313	CaChr1	21506779	(ATC)6	CATATATCCAAGGTGTGGTTG	GTGGTCCAGTGATAATGTTGT	55.0	150	CAATBOX1	CAAT	CAAT promoter consensus sequence found in legA gene of pea	YABBY family of transcriptional regulators
Ca-CNMS60	28367325	CaChr1	21506779	(TC)12	GTAACCCCAGTTGAGAAGTTT	TGCTTTGTAGTGAGTGTGTGT	55.0	147	CTRMCAMV35S	TCTCTCTCT	CaMV 35S (Inverted GAGA) found in a 60-nucleotide region downstream of the transcription start site of the	Basic helix-loop-helix (bHLH) DNA-binding superfamily protein
Ca-CNMS61	28367336	CaChr1	23591832	(TTAT)5	TCGACAACCTTTCAATTTCTTT	TTTTCTTTATGAGAAAACCAAAAACA	57.9	255	TATABOX5	TTATTT	TATA box found in the 5'upstream region of pea glutamine synthetase gene	Like-Sm ribonucleoprotein (LSM) domain
Ca-CNMS62	28367347	CaChr1	24322894	(TATT)5	TTCCAAATCGCACTACCAAA	AATCATGGTACGGTTCCAGC	59.2	238	TATABOX5	TTATTT	TATA box found in the 5'upstream region of pea glutamine synthetase gene	Transposase, Ptta/En/Spm, plant
Ca-CNMS63	28367358	CaChr1	28338833	(TC)8	CCTCCTCTTGTCCATTGCAT	ATGTGGAAAACAGAAAGGCG	60.1	271	CTRMCAMV35S	TCTCTCTCT	CaMV 35S (Inverted GAGA) found in a 60-nucleotide region downstream of the transcription start site of the	Domain of unknown function DUF632
Ca-CNMS64	28367369	CaChr1	28338833	(CT)21	CGCCTTTCTGTTTTCCACAT	TGGTGTGATTCAAAAACCAA	60.1	279	CTRMCAMV35S	TCTCTCTCT	CaMV 35S (Inverted GAGA) found in a 60-nucleotide region downstream of the transcription start site of the	Domain of unknown function DUF632
Ca-CNMS65	28367380	CaChr1	28338833	(CTC)5	AAAGGGGTTGTTTCCCTTTG	GAAAGGAAGGATGCGTGTTG	60.2	185	CTRMCAMV35S	TCTCTCTCT	CaMV 35S (Inverted GAGA) found in a 60-nucleotide region downstream of the transcription start site of the	Domain of unknown function DUF632
Ca-CNMS66	28367391	CaChr1	33167850	(CT)7	AATCTCCCCCAATTTCCATC	GGACCAAAATAACCGTTTAATCC	60.0	204	CTRMCAMV35S	TCTCTCTCT	CaMV 35S (Inverted GAGA) found in a 60-nucleotide region downstream of the transcription start site of the	Helicase, C-terminal
Ca-CNMS67	28367399	CaChr1	35112175	(CAA)4	TTAGGCTTCAACACCACTTTA	CTCTTCAAGAAAAGCAGATTG	55.0	148	RAV1AAT	CAACA	AP2 & B3 like domiane in RAV1	Homeobox-leucine zipper protein
Ca-CNMS68	28367400	CaChr1	35760544	(TC)6	TTTAACCCACCCTTCTTCCC	GAGTAATGGAGCGCGAGAAC	60.2	248	CTRMCAMV35S	TCTCTCTCT	CaMV 35S (Inverted GAGA) found in a 60-nucleotide region downstream of the transcription start site of the	GDP-fucose protein O-fucosyltransferase
Ca-CNMS69	28367401	CaChr1	39713328	(AGC)5	TTCATTTGTACAATGGGCGA	AATCTGGATCGGGGAAAATC	59.9	143	ANAERO2CONSENSUS	AGCAGC	Motif found in silico in promoters of 13 anaerobic genes involved in the fermentative pathway	Isocitrate/isopropylmalate dehydrogenase
Ca-CNMS70	28367403	CaChr1	40182979	(TC)6	ACCAGCACAAGCTCCCTCTA	TTTGGCACCACAAAGTGAAA	60.0	104	CTRMCAMV35S	TCTCTCTCT	CaMV 35S (Inverted GAGA) found in a 60-nucleotide region downstream of the transcription start site of the	Peptidase A1
Ca-CNMS71	28367404	CaChr1	40312615	(TC)6	GACGTGGAACTTTCTCCACC	AAAGGGATGCTTCTGAATGAA	59.6	152	CTRMCAMV35S	TCTCTCTCT	CaMV 35S (Inverted GAGA) found in a 60-nucleotide region downstream of the transcription start site of the	YTH domain
Ca-CNMS72	28367405	CaChr1	40466357	(TC)6	TTCCTTCATTCACTCTCTCGC	GGAATCGGAAAACGAACCTT	59.6	116	CTRMCAMV35S	TCTCTCTCT	CaMV 35S (Inverted GAGA) found in a 60-nucleotide region downstream of the transcription start site of the	Zinc finger, RING-type
Ca-CNMS73	28367406	CaChr1	41855518	(CT)8	TTCCTTTCCTGTCTTCATCTCTG	CCAACCAAACAAAACCCACT	59.9	272	CTRMCAMV35S	TCTCTCTCT	CaMV 35S (Inverted GAGA) found in a 60-nucleotide region downstream of the transcription start site of the	Leucine-rich repeat
Ca-CNMS74	28367407	CaChr1	45051952	(AG)9	CCGCAACTGAGCCAATTTAT	GCTGTACAAGGCTGCAACAA	60.1	229	CTRMCAMV35S	TCTCTCTCT	CaMV 35S (Inverted GAGA) found in a 60-nucleotide region downstream of the transcription start site of the	NmrA-like
Ca-CNMS75	28367408	CaChr1	46533653	(GAG)5	GCGTCGAAAAATCATAACGAG	CGCGCTGTTTAGGTATGGAT	59.7	160	UPRMOTIFIIAT	CCNNNNNNNNNNNNCCAC G	Conserved UPR (unfolded protein response) cis-acting element in Arabidopsis	Pectinacetylesterase
Ca-CNMS76	28367409	CaChr1	46748180	(AG)6	CTGCGTTGTGAGTCTGAAGC	GGTGGTGGTTGTTGTTCCTT	59.8	206	CTRMCAMV35S	TCTCTCTCT	CaMV 35S (Inverted GAGA) found in a 60-nucleotide region downstream of the transcription start site of the	Protein of unknown function DUF3754
Ca-CNMS77	28367410	CaChr1	46912706	(GA)6	TGGGTGTGGTTGTTGTCCTA	AAGCATTTGAGAATGCCACA	59.8	241	CTRMCAMV35S	TCTCTCTCT	CaMV 35S (Inverted GAGA) found in a 60-nucleotide region downstream of the transcription start site of the	Oxoglutarate/iron-dependent oxygenase
Ca-CNMS78	28367411	CaChr1	47764596	(TC)15	CCACCACCTACGTAAAATAAA	GTTTGAACCTCCTTGAAAGAT	55.0	171	CTRMCAMV35S	TCTCTCTCT	CaMV 35S (Inverted GAGA) found in a 60-nucleotide region downstream of the transcription start site of the	TLP family
Ca-CNMS79	28367412	CaChr1	47995317	(CAA)4	TCAGAGTAACAGCAACAACG	CGATGCAAGTTTTAGAGAGAG	55.0	150	RAV1AAT	CAACA	AP2 & B3 like domians in RAV1	Acyl-CoA N-acyltransferase with RING/FYVE/PHD-type zinc finger
Ca-CNMS80	28367414	CaChr1	48128817	(AGA)5	TTATCTGGCCAACTGTGTATT	TCTGTTTTTGGTTTCGTGTAT	55.0	153	POLLENILELAT52	AGAAA	One of two co-dependent regulatory elements responsible for pollen specific activation of tomato lat52	TCP family transcription factor
Ca-CNMS81	28367415	CaChr2	1339469	(CT)9	TGTTTGTTTTTCTGAATCGGTG	TTTCTTTGAGAACAAAATGCGA	60.0	280	CTRMCAMV35S	TCTCTCTCT	CaMV 35S (Inverted GAGA) found in a 60-nucleotide region downstream of the transcription start site of the	Pectate lyase/Amb allergen
Ca-CNMS82	28367416	CaChr2	1581339	(GAAA)5	CGAGGGGTGGTGATATTTTC	CACGAATCACGCTTTCCATA	59.2	102	POLLEN1LELAT52	AGAAA	One of two co-dependent regulatory elements responsible for pollen specific activation of tomato lat52	Pyruvate kinase
Ca-CNMS83	28367417	CaChr2	1645369	(TATT)5	TTGTGCACTTTATTCTTATCTCTCTC A	TGGCGGTCGATAACTTTAGTG	59.9	127	TATABOX5	TTATTT	TATA box found in the 5'upstream region of pea glutamine synthetase gene	TPP-binding enzyme, conserved site
Ca-CNMS84	28367418	CaChr2	1709945	(TTTA)5	TTTCCATTTATCCTCAGCTTG	TGTCTGTTTTGGTGGCAAAG	57.0	220	TATABOX5	TTATTT	TATA box found in the 5'upstream region of pea glutamine synthetase gene	Protein kinase, catalytic domain

Marker IDs	NCBI Probe IDs (PUIDs)	Chromosomes	Physical positions (bp)	Microsatellite repeat-motifs	Forward Primer (5'-3')	Reverse Primer (5'-3')	Actual annealing tempearture (0C)	Size (bp) of alleles amplified	Regulatory elements	Signal sequences of known regulatory element-binding sites	Function of known regulatory elements/ transcription factor-binding sites	Putative gene function
Ca-CNMS85	28367419	CaChr2	1928905	(CT)8	TTCTTTCTGCCCCAAACATC	TTTCGTGGGAAAATTTTGGA	60.1	279	CTRMCAMV35S	TCTCTCTCT	CaMV 35S (Inverted GAGA) found in a 60-nucleotide region downstream of the transcription start site of the	Germin
Ca-CNMS86	28367420	CaChr2	2144472	(TC)6	GGTGAAGGTGAATTGAAGGC	TGAGCTGGAAAGGGTAATGG	59.5	280	CTRMCAMV35S	TCTCTCTCT	CaMV 35S (Inverted GAGA) found in a 60-nucleotide region downstream of the transcription start site of the	ATPase, P-type, K/Mg/Cd/Cu/Zn/Na/Ca/Na/H-
Ca-CNMS87	28367421	CaChr2	2209993	(GTT)7	AACTCTAGGTAGTTGGATAAGGCG	TCAACATCAACAGTTGTCATTCC	59.3	165	CAREOSREP1	CAACTC	Motif found in REP-1 gene promoter	Fumarate reductase/succinate dehydrogenase flavoprotein, N-
Ca-CNMS88	28367422	CaChr2	2249607	(AG)9	GACTGCACCGTAAAACCACA	TCCGCTTTTTCCGTTTTAGA	59.6	142	CTRMCAMV35S	TCTCTCTCT	CaMV 35S (Inverted GAGA) found in a 60-nucleotide region downstream of the transcription start site of the	Prenyltransferase/squalene oxidase
Ca-CNMS89	28367423	CaChr2	2409924	(ATCA)3	CATATATCCAAGGTGTGGTTG	GTGGTCCAGTGATAATGTTGT	55.0	150	GATABOX	GATA	GATA motif in CaMV 35S promoter required for high level, light regulated and tissue specific expression	YABBY family of transcriptional regulators
Ca-CNMS90	28367425	CaChr2	2409924	(AG)11	TGAGAAAAATTAGCACACAAAG	ACATATTCATGCTGTTGTTCC	55.0	143	GAGA8HVBKN3	(GA)8	Motif found in intron IV of the barley (H.v.) gene Bkn3	Homeodomain leucine zipper class I (HD-Zip I) transcriptional activator
Ca-CNMS91	28367426	CaChr2	2414410	(CCG)5	AATCCCTAACTCGTTCTCAAC	GAGAATAGAGCGAGTGTCTTG	55.0	147	GCC CORE	GCCGCC	Motif found in many pathogen-responsive genes such as PDF1.2, Thi2.1 and PR4	Protein with a methyl-CpG-binding domain
Ca-CNMS92	28367427	CaChr2	2414410	(CCA)4	AATCCCTAACTCGTTCTCAAC	GAGAATAGAGCGAGTGTCTTG	55.0	147	GCC CORE	GCCGCC	Motif found in many pathogen-responsive genes such as PDF1.2, Thi2.1 and PR4	Protein with a methyl-CpG-binding domain
Ca-CNMS93	28367428	CaChr2	2798817	(GA)11	TTTCCCAGATTTTCCCAAGA	GCACGTACCACCGTTTCTCT	59.5	225	GAGA8HVBKN3	GAGAGAGAGAGAGAGA	Motif found in intron IV of the barley (H.v.) gene Bkn3	Paired amphipathic helix
Ca-CNMS94	28367429	CaChr2	3202091	(GTT)4	TCTCTCTTTCTCTCTCTCTCTCA	GGTAAGTCTTCATAACGAACG	55.0	172	RAV1AAT	CAACA	AP2 & B3 like domians in RAV1	Heat Stress Transcription Factor (Hsf) family
Ca-CNMS95	28367430	CaChr2	3202091	(TATAG)4	CCATGGATAAAGAAGAAATGA	TTCTTCTTTATCCATGGCTTT	54.0	145	GATABOX	GATA	GATA motif in CaMV 35S promoter required for high level, light regulated, and tissue specific expression	Homeobox-containing gene with an unusual feature
Ca-CNMS96	28367431	CaChr2	3202091	(AGATAT)3	CCATGGATAAAGAAGAAATGA	CCAAACTCCTCTGATCCATA	54.0	145	GATABOX	GATA	GATA motif in CaMV 35S promoter required for high level, light regulated, and tissue specific expression	Homeobox-containing gene with an unusual feature
Ca-CNMS97	28367432	CaChr2	3290707	(TA)6	AAATGGCAATATGAAGTAAGTAATGA A	AAAAAGAAAGCACTACCGTCTTC	58.6	260	GAGA8HVBKN3	GAGAGAGAGAGAGAGA	Motif found in intron IV of the barley (H.v.) gene Bkn3	Cullin, N-terminal
Ca-CNMS98	28367433	CaChr2	3541793	(AG)12	TACCACTTTTATACGCTGCAC	ATGAATGAACGAATGTGACTC	56.0	145	GAGA8HVBKN3	(GA)8	Motif found in intron IV of the barley (H.v.) gene Bkn3	HAIRY MERISTEM 3 (HAM3)
Ca-CNMS99	28367434	CaChr2	3544818	(GA)10	CCTCTGAAATGGGACTGTT	AACACTTCCCCACACAAAC	55.0	141	GAGA8HVBKN3	(GA)8	Motif found in intron IV of the barley (H.v.) gene Bkn3	ERF (ethylene response factor) subfamily B-1 of ERF/AP2
Ca-CNMS100	28366771	CaChr2	3547678	(GA)13	ATGGAGGCGAATATATAGGAG	TTTTAAGAAACAACTGCGTTC	55.0	151	GAGA8HVBKN3	(GA)8	Motif found in intron IV of the barley (H.v.) gene Bkn3	LOB domain-containing protein 40 (LBD40)
Ca-CNMS101	28366772	CaChr2	5660440	(TC)6	TTGACCCTTTCGAATTTTGC	TGCAGGGAGTTAATTGTGGA	60.1	222	CTRMCAMV35S	TCTCTCTCT	CaMV 35S (Inverted GAGA) found in a 60-nucleotide region downstream of the transcription start site of the	SWIB/MDM2 domain
Ca-CNMS102	28366773	CaChr2	8343253	(CT)20	TATTTCGCGTTGTTGCAAAG	TTTGATCGGAAAACCCTCAG	59.9	236	CTRMCAMV35S	TCTCTCTCT	CaMV 35S (Inverted GAGA) found in a 60-nucleotide region downstream of the transcription start site of the	Ras small GTPase, Rab type
Ca-CNMS103	28366774	CaChr2	9905753	(AG)6	AGGTCCACTTGTTGCTGCTT	CAAATATTTCCATCGTCCCG	59.9	237	CTRMCAMV35S	TCTCTCTCT	CaMV 35S (Inverted GAGA) found in a 60-nucleotide region downstream of the transcription start site of the	Polyadenylate-binding protein/Hyperplastic disc protein
Ca-CNMS104	28366775	CaChr2	10327518	(TTTA)3	GGTAAGGTTGCTTGGTTAAAG	ATCATCACCATCACCACTTAC	56.0	151	TATABOX5	TTATTT	TATA box found in the 5'upstream region of pea glutamine synthetase gene	IAA-leucine resistant3 (ILR3)
Ca-CNMS105	28366776	CaChr2	11261494	(TC)13	CCACCACACAGAAGAAAGAA	GGTGGTGTGTGTAGTTGTGA	56.0	148	CTRMCAMV35S	TCTCTCTCT	CaMV 35S (Inverted GAGA) found in a 60-nucleotide region downstream of the transcription start site of the	homeodomain leucine zipper class I (HD-Zip I) transcriptional activator
Ca-CNMS106	28366777	CaChr2	11473517	(TTTA)6	CAGCATTGGATTCTCAGGGT	CAAGAGTCAACTTCACAGTTCCA	60.1	233	TATABOX5	TTATTT	TATA box found in the 5'upstream region of pea glutamine synthetase gene	IQ motif, EF-hand binding site
Ca-CNMS107	28366778	CaChr2	11702179	(AGT)5	CCCTTTTGCCTTGGTCTACA	TGGCTACAACAGATTCCAACA	60.1	187	CACTFTPPCA1	YACT	Tetranucleotide (CACT) is a key component of Mem1 found in the cis-regulatory element in the distal region of	Helix-loop-helix DNA-binding domain
Ca-CNMS108	28366779	CaChr2	12579708	(ACT)5	CAATGCGACCACAACCACTA	AGCCTCGACATGTTTTGCTT	60.6	176	CACTFTPPCA1	YACT	Tetranucleotide (CACT) is a key component of Mem1 found in the cis-regulatory element in the distal region of	Mitochondrial transcription termination factor-related
Ca-CNMS109	28366780	CaChr2	12580073	(GTT)4	GTTTTGTTTGAAGGCTTTTTC	CCAAATCTCATCAATTCTTCA	56.0	149	CAREOSREP1	CAACTA	Found in REP-1 gene promoter	A locus involved in embryogenesis
Ca-CNMS110	28366782	CaChr2	14597266	(CTTG)4	CCAACAAACAACACTTCAAAC	TGAACTCAGAATTTGGATGAT	56.0	162	CACTFTPPCA1	YACT	Tetranucleotide (CACT) is a key component of Mem1 found in the cis-regulatory element in the distal region of	Subunit of CCAAT-binding complex
Ca-CNMS111	28366783	CaChr2	14623895	(AAG)4	ATCAGAATTTGACAAGGAGGT	CTTCTTCTTTTGATGGTGTTG	55.0	153	DOFCOREZM	AAAG	Site required for binding of Dof proteins in maize	NGATHA3 (NGA3)
Ca-CNMS112	28366784	CaChr2	15343933	(CT)7	ACCGCAACGGTTTAAAATTG	CGCGAGAGAGAGAAGAGGAA	59.9	237	CTRMCAMV35S	TCTCTCTCT	CaMV 35S (Inverted GAGA) found in a 60-nucleotide region downstream of the transcription start site of the	Protein kinase, catalytic domain
Ca-CNMS113	28366785	CaChr2	15468290	(GA)10	TGAGCAAAGGAAAGACACGA	GGCATGTGTCGTATGAATGC	59.6	233	GAGA8HVBKN3	GAGAGAGAGAGAGAGA	Motif found in intron IV of the barley (H.v.) gene Bkn3	Pterin-binding
Ca-CNMS114	28366786	CaChr2	15706984	(AG)8	TGGTCGTAATAGCAAGAGCTGA	TGATGAGACCTACGTCACGG	60.0	243	CTRMCAMV35S	TCTCTCTCT	CaMV 35S (Inverted GAGA) found in a 60-nucleotide region downstream of the transcription start site of the	Peptidase C48, SUMO/Sentrin/Ubl1
Ca-CNMS115	28366787	CaChr2	17714382	(TAG)5	TGAAACCCCTGATTGTTGCT	GATTCCACCGTTAGGACGAA	60.5	253	CACTFTPPCA1	YACT	Tetranucleotide (CACT) is a key component of Mem1 found in the cis-regulatory element in the distal region of	S-adenosylmethionine decarboxylase
Ca-CNMS116	28366788	CaChr2	20435377	(TC)6	CCAACATTTTGTTGATTCTCCA	CACTCAAGTTGCAACGAGAAAA	59.8	275	CTRMCAMV35S	TCTCTCTCT	CaMV 35S (Inverted GAGA) found in a 60-nucleotide region downstream of the transcription start site of the	Cryptochrome/DNA photolyase, class 1
Ca-CNMS117	28366789	CaChr2	24204662	(GA)6	GCAAAATAAGCACAAGTGAAA	TTTTCAATCCATTCAGGTAAG	56.0	149	GATABOX	GATA	GATA motif in CaMV 35S promoter required for high level, light regulated and tissue specific expression	Zinc ion binding
Ca-CNMS118	28366790	CaChr2	25144959	(CT)6	TTGGAACCAGCTTTTCTGCT	GGAAGAAAGGGATAAGGCAAA	60.0	101	CTRMCAMV35S	TCTCTCTCT	CaMV 35S (Inverted GAGA) found in a 60-nucleotide region downstream of the transcription start site of the	SANT domain, DNA binding
Ca-CNMS119	28366791	CaChr2	26083292	(AG)9	CTTCCGCAATTATTCATGCC	CTATCCGGTTTGCGTCAACT	60.4	183	CTRMCAMV35S	TCTCTCTCT	CaMV 35S (Inverted GAGA) found in a 60-nucleotide region downstream of the transcription start site of the	APO protein, plant
Ca-CNMS120	28366793	CaChr2	26296091	(TC)8	TCCTATGTTTCGTTCTCACCG	GCCAAAGAGCTAGCCAAAAA	60.1	204	CTRMCAMV35S	TCTCTCTCT	CaMV 35S (Inverted GAGA) found in a 60-nucleotide region downstream of the transcription start site of the	Cyclin
Ca-CNMS121	28366794	CaChr2	27873320	(CT)7	GAACTCCAACGGACCTCAAA	GAGTGGGAACGGTGTTCAAG	60.1	132	CTRMCAMV35S	TCTCTCTCT	CaMV 35S (Inverted GAGA) found in a 60-nucleotide region downstream of the transcription start site of the	Cystathionine beta-synthase, core
Ca-CNMS122	28366795	CaChr2	28061906	(TTTA)5	TTCTTTTGAGTTGATGGTAGGTTC	TGAGATTATGACACATGCCTGA	58.7	207	TATABOX5	TTATTT	TATA box found in the 5'upstream region of pea glutamine synthetase gene	Uncharacterised protein family UPF0497, trans-membrane plant
Ca-CNMS123	28366796	CaChr2	29986492	(GA)6	TAATTAGGGGAACGGACGTG	CAGTTCCTTTCTGACACGCA	59.8	204	CTRMCAMV35S	TCTCTCTCT	CaMV 35S (Inverted GAGA) found in a 60-nucleotide region downstream of the transcription start site of the	Protein phosphatase 2C, manganese/magnesium aspartate
Ca-CNMS124	28366797	CaChr2	30031255	(CT)7	GGAAAAAGGAAAAACGAATAC	AGCTTCAAATTGTAATTGCTG	55.0	153	CTRMCAMV35S	TCTCTCTCT	CaMV 35S (Inverted GAGA) found in a 60-nucleotide region downstream of the transcription start site of the	Calmodulin-binding transcription activator protein
Ca-CNMS125	28366798	CaChr2	30512114	(TTG)5	CCCCTCAATTCCTTTTCCAT	TCATTTGTTTGGTTGTTTCGAC	60.1	251	CAREOSREP1	CAACTC	Motif found in REP-1 gene promoter	Hs1pro-1, C-terminal
Ca-CNMS126	28366799	CaChr2	30866411	(CAA)4	ATCGAGTTCTTCTCGATCTCT	CTACCATCTGCTCTTGCTCTA	55.0	149	RAV1AAT	CAACA	AP2 & B3 like domiane in RAV1	CZF1
Ca-CNMS127	28366800	CaChr2	31059800	(AG)7	TGAAAGCAAGGACTAGCGAAA	TCCATCGATTCCTATGGCTC	60.1	184	CTRMCAMV35S	TCTCTCTCT	CaMV 35S (Inverted GAGA) found in a 60-nucleotide region downstream of the transcription start site of the	Rad21/Rec8-like protein, C-terminal, eukaryotic

Marker IDs	NCBI Probe IDs (PUIDs)	Chromosomes	Physical positions (bp)	Microsatellite repeat-motifs	Forward Primer (5'-3')	Reverse Primer (5'-3')	Actual annealing tempearture (0C)	Size (bp) of alleles amplified	Regulatory elements	Signal sequences of known regulatory element-binding sites	Function of known regulatory elements/ transcription factor-binding sites	Putative gene function
Ca-CNMS128	28366801	CaChr2	32518138	(CT)6	ACACCAAAATGATGGGTTCC	TCATGCATTGTTTGATTGCC	59.5	211	CTRMCAMV35S	TCTCTCTCT	CaMV 35S (Inverted GAGA) found in a 60-nucleotide region downstream of the transcription start site of the	Peptidase C19, ubiquitin carboxyl-terminal hydrolase 2
Ca-CNMS129	28366802	CaChr2	32535474	(AGC)5	TTCATTTGTACAATGGGCGA	AATCTGGATCGGGGAAAATC	59.9	143	ANAERO2CONSENSUS	AGCAGC	Motif found in silico in promoters of 13 anaerobic genes involved in the fermentative pathway	Peptidase C48, SUMO/Sentrin/Ubl1
Ca-CNMS130	28366804	CaChr2	32955867	(GAGAGT)3	AGGTTGGTTGTCTTAAGTTGA	TTTCTGCTTCACTTGATCTTC	54.0	149	SORLIP5AT	GAGTGAG	Over-Represented in Light-Induced Promoters (SORLIPs) in Arabidopsis	Pseudo-response regulator
Ca-CNMS131	28366805	CaChr2	33808957	(ATATAC)5	CAATAAGTGGGGAGGAAGGTT	TTACAAAAACCACCCACGCT	59.3	251	P1BS	GNATATNC	PHR1-binding sequence found in the upstream regions of phosphate starvation responsive plant genes	WD40 repeat
Ca-CNMS132	28366806	CaChr2	34094437	(CT)6	GCTTACAAACATCATCGGCA	CCAAAGAAGGCAAGACTCAGA	59.7	104	CTRMCAMV35S	TCTCTCTCT	CaMV 35S (Inverted GAGA) found in a 60-nucleotide region downstream of the transcription start site of the	BTB/POZ-like
Ca-CNMS133	28366807	CaChr2	34347441	(CAG)5	TGGATTTTCCACCACAATCA	CATTGCATGACACACAAAAGG	59.8	120	ANAERO2CONSENSUS	AGCAGC	Motif found in silico in promoters of 13 anaerobic genes involved in the fermentative pathway	Protein kinase, catalytic domain
Ca-CNMS134	28366808	CaChr2	34791092	(CAAAAT)6	CAATCATGGGTGGTTGTTTG	TTCATGGTGATTGTGATTTTGA	59.7	197	ARR1AT	NGATT	Motif found in the promoter of rice NSHB gene	Protein kinase, catalytic domain
Ca-CNMS135	28366809	CaChr2	35133394	(AAC)5	CCACACACAAGTACAATACCACC	AGGCAGGGGAAGATCTAGGA	59.2	237	RAV1AAT	CAACA	AP2 & B3 like domiane in RAV1	Cell division protein FtsZ, N-terminal
Ca-CNMS136	28366810	CaChr2	35843818	(TC)7	GCAACCGTGTCTCTCTCTCC	CTGTGATTCCGTGGAGGTTT	60.0	264	CTRMCAMV35S	TCTCTCTCT	CaMV 35S (Inverted GAGA) found in a 60-nucleotide region downstream of the transcription start site of the	Ribosomal protein L47, mitochondrial
Ca-CNMS137	28366811	CaChr2	35958011	(TC)11	CCAGTTCTCTGCTTTGGACC	TGTTGTGGTTGGATTTGTGG	59.8	106	CTRMCAMV35S	TCTCTCTCT	CaMV 35S (Inverted GAGA) found in a 60-nucleotide region downstream of the transcription start site of the	F-box domain, cyclin-like
Ca-CNMS138	28366812	CaChr2	35967304	(ACA)5	GGATAGCACATGAAACGCAG	GGCCGCAGTTAGAGATGTTC	59.3	251	RAV1AAT	CAACA	AP2 & B3 like domiane in RAV1	Ribosomal protein S26e
Ca-CNMS139	28366813	CaChr2	36090310	(CAA)4	TCTTGGATTTGTATTCCCTTT	ATACTGATACCAATGGAGTCG	55.0	146	RAV1AAT	CAACA	AP2 & B3 like domians in RAV1	WRKY1
Ca-CNMS140	28366815	CaChr2	36202138	(TC)16	TCTCATTCTTCGTCCTTCCC	AAATGAGAGTGAATATCAAAGGCA	59.2	210	CTRMCAMV35S	TCTCTCTCT	CaMV 35S (Inverted GAGA) found in a 60-nucleotide region downstream of the transcription start site of the	FAS1 domain
Ca-CNMS141	28366816	CaChr2	36378087	(TCG)5	CGGTTTCGTTGCTCATAGGT	TTCCGAACCAGACCATGAAT	60.1	245	CGACGOSAMY3	CGACG	Amy3D & Amy 3E alpha-amylase gene	Domain of unknown function DUF632
Ca-CNMS142	28366817	CaChr2	36378087	(GTT)6	GCGGTGGGAGTGTAATGAGT	ACCTATGAGCAACGAAACCG	60.0	279	CAREOSREP1	CAACTC	Motif found in REP-1 gene promoter	Domain of unknown function DUF632
Ca-CNMS143	28366818	CaChr2	36561873	(TC)6	CTTTTCACCATTCACGCAGA	AGATATGGTCGCGGAAGAGA	59.8	119	CTRMCAMV35S	TCTCTCTCT	CaMV 35S (Inverted GAGA) found in a 60-nucleotide region downstream of the transcription start site of the	Transketolase-like, pyrimidine-binding domain
Ca-CNMS144	28366819	CaChr3	4823609	(TTTG)3	TTCTTTTCTCTCTTGCATTTG	CCAAAATCATCATCATAAACG	55.0	164	ANAERO1CONSENSUS	AAACAAA	Motif found in silico in promoters of 13 anaerobic genes involved in the fermentative pathway	POZ/BTB containing-protein AtPOB1
Ca-CNMS145	28366820	CaChr3	6937156	(TC)6	TTCCCACACCAACCCTTCTA	CAAGCACAGAATGACCCAAA	60.3	271	CTRMCAMV35S	TCTCTCTCT	CaMV 35S (Inverted GAGA) found in a 60-nucleotide region downstream of the transcription start site of the	Protein of unknown function DUF724
Ca-CNMS146	28366821	CaChr3	8023581	(ACTC)3	CATCCCTCTTTTTATTCAACA	CTAAAAATGGAGGAGGAAGAG	55.0	151	CACTFTPPCA1	YACT	Tetranucleotide (CACT) is a key component of Mem1 found in the cis-regulatory element in the distal region of	NAC domain transcription factor
Ca-CNMS147	28366822	CaChr3	8996627	(GA)8	CCACTCCAAGTGGAGGGTAA	GACCTATGTTGCATGTTTCTCG	60.0	248	GAGA8HVBKN3	GAGAGAGAGAGAGAGA	Motif found in intron IV of the barley (H.v.) gene Bkn3	Reverse transcriptase, RNA-dependent DNA polymerase
Ca-CNMS148	28366823	CaChr3	9563117	(TTAT)5	TGGTCCCATATGAGTTTTTGG	CCAAGGCTAAGACCAGTTCAA	59.7	125	TATABOX5	TTATTT	TATA box found in the 5'upstream region of pea glutamine synthetase gene	NA
Ca-CNMS149	28366824	CaChr3	9911187	(AG)16	CGACAACGAACGAGAGTGAA	AAATCTGAACAAACCACCGC	60.0	168	GAGA8HVBKN3	GAGAGAGAGAGAGAGA	Motif found in intron IV of the barley (H.v.) gene Bkn3	RNA recognition motif domain
Ca-CNMS150	28366826	CaChr3	14022456	(TCTCT)3	GCCCTTGAGTTGAGTTAAGAC	AAACTATGGAAATGGAAGGTT	56.0	145	NODCON2GM	CTCTT	Putative nodulin consensus sequences	Auxin (indole-3-acetic acid) induced gene
Ca-CNMS151	28366827	CaChr3	16813157	(CT)10	CAACCACATCAATCCCCTTC	GTGGGGTGTGAAAAGGAAGA	60.2	106	CTRMCAMV35S	TCTCTCTCT	CaMV 35S (Inverted GAGA) found in a 60-nucleotide region downstream of the transcription start site of the	Helix-loop-helix DNA-binding domain
Ca-CNMS152	28366828	CaChr3	16814157	(TCTA)3	TGCTCAATTTTTAGCTTCAAC	ATCTGAAACAAGAGACCCACT	55.0	166	GATABOX	GATA	GATA motif in CaMV 35S promoter required for high level, light regulated, and tissue specific expression	ABA-responsive element binding protein with a bZIP domain
Ca-CNMS153	28366829	CaChr3	18108283	(CT)16	TTTTTGTGATGAAACGAAAACA	AAGTTCCCAGAGCAGCAGAG	58.2	113	CTRMCAMV35S	TCTCTCTCT	CaMV 35S (Inverted GAGA) found in a 60-nucleotide region downstream of the transcription start site of the	Transcription factor GRAS
Ca-CNMS154	28366830	CaChr3	19075758	(CTT)9	TGTATCAAGTAGAGCAGCACA	GCATGAAAATTCAGAGAGAAA	55.0	150	CACTFTPPCA1	YACT	Tetranucleotide (CACT) is a key component of Mem1 found in the cis-regulatory element in the distal region of	GLK1, Golden2-like 1
Ca-CNMS155	28366831	CaChr3	20506092	(CAA)5	AATACTTCTATGTTTCACCAACA	ATTGGTGATAATGAAGGAGGT	53.0	163	RAV1AAT	CAACA	AP2 & B3 like domiane in RAV1	Plant WRKY transcription factor
Ca-CNMS156	28366832	CaChr3	20828026	(CATCAC)3	ACCATCATAACCATCATCATC	GGAAACCCTAGGTATTGTTGT	55.0	143	ANAERO3CONSENSUS	TCATCAC	Motif found in silico in promoters of 13 anaerobic genes involved in the fermentative pathway	One of two Arabidopsis RAPTOR/KOG1 homologs
Ca-CNMS157	28366833	CaChr3	21438318	(AAAG)5	TAAACTCCTCAGGCCCAGAA	AAATTTGTTGGCAAAGCAGG	59.8	217	POLLEN1LELAT52	AGAAA	Elements responsible for pollen specific activation of tomato lat52 gene	ATPase, P-type, K/Mg/Cd/Cu/Zn/Na/Ca/Na/H-
Ca-CNMS158	28366834	CaChr3	21537552	(ATCCA)3	GTGCACCAGTCATCAAGTAAT	CATGAATTGAGAACGAAGAAG	55.0	138	CCAATBOX1	CCAAT	Motif found in the promoter of heat shock protein genes	Nuclear acetyltransferase (NSI)
Ca-CNMS159	28366835	CaChr3	21537552	(CAAAC)3	GTGCACCAGTCATCAAGTAAT	CATGAATTGAGAACGAAGAAG	55.0	138	MYB1AT	WAACCA	Motif found in the promoters of the dehydration-responsive gene rd22	Nuclear acetyltransferase (NSI)
Ca-CNMS160	28366837	CaChr3	21626330	(TC)10	TCCGAAATGGTTGCTTCTCT	TGTGGTTGACTTGCTTGCTC	59.8	160	CTRMCAMV35S	TCTCTCTCT	CaMV 35S (Inverted GAGA) found in a 60-nucleotide region downstream of the transcription start site of the	Protein of unknown function DUF1191
Ca-CNMS161	28366838	CaChr3	21773307	(CAA)6	CCATGTCTCCCCCTTAATCA	ACTGCTGAGGTTGCTGGAGT	59.7	198	RAV1AAT	CAACA	AP2 & B3 like domiane in RAV1	Transcription factor GRAS
Ca-CNMS162	28366839	CaChr3	22656875	(TC)41	CATCCACATGCAAATTTACCC	TGATGCCGATGATTGAAAAA	60.1	249	CTRMCAMV35S	TCTCTCTCT	CaMV 35S (Inverted GAGA) found in a 60-nucleotide region downstream of the transcription start site of the	SPX, N-terminal
Ca-CNMS163	28366840	CaChr3	22881608	(CCCAA)5	GAGTTTCGAACAAATGGGGA	TTATTGGACGTTAGTCGGGC	59.9	271	SEF3MOTIFGM	AACCCA	Motif found in the 5' upstream region of beta-conglycinin (7S globulin) gene	Ribosomal protein L2
Ca-CNMS164	28366841	CaChr3	23897882	(TC)8	ACGAAATCCAACGTATACAAA	TCCTTGAAGAATCCACAAATA	55.0	153	CTRMCAMV35S	TCTCTCTCT	CaMV 35S (Inverted GAGA) found in a 60-nucleotide region downstream of the transcription start site of the	Auxin response factor 9 (ARF9)
Ca-CNMS165	28366842	CaChr3	24037924	(TC)7	CTCAATTTCTTCGACCTCCG	GGCTCAAAATGGTTCAAGGA	59.8	214	CTRMCAMV35S	TCTCTCTCT	CaMV 35S (Inverted GAGA) found in a 60-nucleotide region downstream of the transcription start site of the	Protein phosphatase 2C-like
Ca-CNMS166	28366843	CaChr3	24037924	(CT)9	CTCAATTTCTTCGACCTCCG	GGCTCAAAATGGTTCAAGGA	59.8	214	CTRMCAMV35S	TCTCTCTCT	CaMV 35S (Inverted GAGA) found in a 60-nucleotide region downstream of the transcription start site of the	Protein phosphatase 2C-like
Ca-CNMS167	28366844	CaChr3	24190588	(TC)6	TCGATTGTATATATGCATGTGTGTG	TAGAGTAAGGGGCGTAGCCA	59.7	198	CTRMCAMV35S	TCTCTCTCT	CaMV 35S (Inverted GAGA) found in a 60-nucleotide region downstream of the transcription start site of the	Aminoacyl-tRNA synthetase, class II (D/K/N)
Ca-CNMS168	28366845	CaChr3	25265322	(TTAT)5	TTATATCGCCGCACCTTCTC	TGAAATCACGATGCCGATAA	60.2	226	TATABOX5	TTATTT	TATA box found in the 5'upstream region of pea glutamine synthetase gene	Translation initiation factor IF2/IF5
Ca-CNMS169	28366846	CaChr3	26542626	(TTG)6	AGAAAACAAAGAGAAGGAAACA	TGAAGAAGAAGCACTTAATGG	55.0	149	RAV1AAT	CAACA	AP2 & B3 like domiane in RAV1	Putative transcription factor; KANADI family
Ca-CNMS170	28366848	CaChr3	26594171	(TTG)7	GCTTTCCCTGGAATTGTGAA	GGAAGTGGATGTTGGAATGG	60.1	204	CAREOSREP1	CAACTC	Motif found in REP-1 gene promoter	Zinc finger, GATA-type

Marker IDs	NCBI Probe IDs (PUIDs)	Chromosomes	Physical positions (bp)	Microsatellite repeat-motifs	Forward Primer (5'-3')	Reverse Primer (5'-3')	Actual annealing tempearture (0C)	Size (bp) of alleles amplified	Regulatory elements	Signal sequences of known regulatory element-binding sites	Function of known regulatory elements/ transcription factor-binding sites	Putative gene function
Ca-CNMS171	28366849	CaChr3	27910720	(TC)9	TCTCTTCCTAGCCAGAAACGTC	TGAAAATGAAAATGGGTGGT	60.0	202	CTRMCAMV35S	TCTCTCTCT	CaMV 35S (Inverted GAGA) found in a 60-nucleotide region downstream of the transcription start site of the	Glycoside hydrolase, carbohydrate-binding
Ca-CNMS172	28366850	CaChr3	28287921	(GCT)5	CCCTCCTGAAGCATTAGCTG	CATTCACCATCAGCAGCAGT	60.0	223	ANAERO2CONSENSUS	AGCAGC	Motif found in silico in promoters of 13 anaerobic genes involved in the fermentative pathway	Myb, DNA-binding
Ca-CNMS173	28366851	CaChr3	28288013	(AG)10	GATTAATGGTTGTGATGAGGA	GTAAATTACCGTGTTTGTTCG	55.0	144	GAGA8HVBKN3	(GA)8	Motif found in intron IV of the barley (H.v.) gene Bkn3	Homeodomain-like superfamily protein
Ca-CNMS174	28366852	CaChr3	28288013	(TTTGAT)3	GATTAATGGTTGTGATGAGGA	CACTATCCACCTTGTTGTCTC	55.0	144	ARR1AT	NGATT	Motif found in the promoter of rice NSHB gene	Homeodomain-like superfamily protein
Ca-CNMS175	28366853	CaChr3	28309747	(AGAA)5	ACAGCTGCTGCTTGAGCATT	GAAGAAGAACGGTGTAGCCG	61.3	248	POLLEN1LELAT52	AGAAA	Elements responsible for pollen specific activation of tomato lat52 gene	Cyclin, C-terminal
Ca-CNMS176	28366854	CaChr3	28311723	(AGAA)5	ACAGCTGCTGCTTGAGCATT	GAAGAAGAACGGTGTAGCCG	61.3	248	POLLEN1LELAT52	AGAAA	Elements responsible for pollen specific activation of tomato lat52 gene	NA
Ca-CNMS177	28366855	CaChr3	28776424	(AG)8	TGCAATGTTCCTGACCACAT	CTTCACCGCTACTCACACCA	60.0	173	CTRMCAMV35S	TCTCTCTCT	CaMV 35S (Inverted GAGA) found in a 60-nucleotide region downstream of the transcription start site of the	RNA polymerase, subunit H/Rpb5 C-terminal
Ca-CNMS178	28366856	CaChr3	29568042	(GGTT)3	AAAAAGACGAACACAGCACTA	CACGTTTCGTTAACTCACTCT	55.0	153	MYBPLANT	MACCWAMC	Sequence related to box P in promoters of phenylpropanoid biosynthetic genes such as PAL, CHS	Zinc finger protein
Ca-CNMS179	28366857	CaChr3	29688873	(TCT)5	ACCGAATCAAACCCCTCTCT	GCTCACCGATCCTGACACTC	59.9	228	GAGA8HVBKN3	GAGAGAGAGAGAGAGA	Motif found in intron IV of the barley (H.v.) gene Bkn3	Zinc finger, AN1-type
Ca-CNMS180	28366859	CaChr3	29844826	(GA)8	TCCAAAAATCCGAATGTGGT	TCCTTGGGTTTCACTTTTGG	60.2	254	GAGA8HVBKN3	GAGAGAGAGAGAGAGA	Motif found in intron IV of the barley (H.v.) gene Bkn3	Basic-leucine zipper (bZIP) transcription factor
Ca-CNMS181	28366860	CaChr3	30319088	(TCATT)3	ATTAGACCTGTGGAAACCATT	CATCATCTTCAATATCCCAAC	55.0	141	INRNTPSADB	YTCANTYY	Motif found in the tobacco psaDb gene promoter without TATA boxes	BSD domain-containing protein
Ca-CNMS182	28366861	CaChr3	30787023	(AG)19	CTTTTTGGTCGGTCGTGTTT	CAAGCCATTGTTCTGTTCCA	60.0	271	GAGA8HVBKN3	GAGAGAGAGAGAGAGA	Motif found in intron IV of the barley (H.v.) gene Bkn3	KIP1-like
Ca-CNMS183	28366862	CaChr3	30787023	(TTG)6	TGGAACAGAACAATGGCTTG	GAAGATCATGAGATGGACAGCA	59.7	200	CAREOSREP1	CAACTC	Motif found in REP-1 gene promoter	KIP1-like
Ca-CNMS184	28366863	CaChr3	30954851	(ATTCAT)6	CCTTTCATTCATCACTTCAAC	AGGATAAAGGTGAAGCAAATC	55.0	165	ROOTMOTIFTAPOX1	ATATT	Motif found both in promoters of rolD	Basic helix-loop-helix (bHLH) DNA-binding superfamily protein
Ca-CNMS185	28366864	CaChr3	31340183	(CT)31	TTCCTGTCTCCATCCAAACC	GGTGAAACAAACTCAAACTTGG	59.9	194	CTRMCAMV35S	TCTCTCTCT	CaMV 35S (Inverted GAGA) found in a 60-nucleotide region downstream of the transcription start site of the	Domain of unknown function DUF640
Ca-CNMS186	28366865	CaChr3	31476599	(TTTA)5	GCTTCCATTTGTGACACCCT	CAGTTGGCAGTTGCATGAAT	60.0	207	TATABOX5	TTATTT	TATA box found in the 5'upstream region of pea glutamine synthetase gene	Glucose-methanol-choline oxidoreductase, N-terminal
Ca-CNMS187	28366866	CaChr3	32505326	(ACA)5	ACCATTGCACAACATTGCAT	ATGTCAACGCTGGTTCTTCC	59.9	221	RAV1AAT	CAACA	AP2 & B3 like domiane in RAV1	Histone H2B
Ca-CNMS188	28366867	CaChr3	32877023	(TC)7	TAAAGCGTATTCAGCCACCC	TTGAGAGTTTTGGGTTTGGG	60.1	180	CTRMCAMV35S	TCTCTCTCT	CaMV 35S (Inverted GAGA) found in a 60-nucleotide region downstream of the transcription start site of the	Helicase, C-terminal
Ca-CNMS189	28366868	CaChr3	34764538	(TTA)8	ATCATCCATCGTTCGCAGTT	TGAGGGTAAAATGTGGAGGG	60.5	234	TATABOX5	TTATTT	TATA box found in the 5'upstream region of pea glutamine synthetase gene	F-box domain, cyclin-like
Ca-CNMS190	28366870	CaChr3	35273746	(CT)10	TGTGAATCACCCACGGTAGA	ACTTCCTTCTCCTTTTCCGC	60.0	222	CTRMCAMV35S	TCTCTCTCT	CaMV 35S (Inverted GAGA) found in a 60-nucleotide region downstream of the transcription start site of the	F-box domain, cyclin-like
Ca-CNMS191	28366871	CaChr3	36218315	(GA)9	TTGAGAGAGCCAAAATTGCTT	CTCCTTTGCCCAAGGTATCA	59.1	264	GAGA8HVBKN3	GAGAGAGAGAGAGAGA	Motif found in intron IV of the barley (H.v.) gene Bkn3	Zinc finger, B-box
Ca-CNMS192	28366872	CaChr3	36673006	(CTAA)5	TGTTGTTAGCATTCACGCTATTC	CGTTAAACAATTTCCGTTTTCA	59.3	254	MYB1LEPR	GTTAGTT	Tomato Pti4(ERF) regulates defence-related gene expression via GCC box and non-GCC box cis elements	Glycosyl transferase, family 8
Ca-CNMS193	28366873	CaChr3	37094171	(AATTGT)4	GGAACAACTCAAGCTCAGTAA	AAAGTGTACACCGAAGCAAT	55.0	164	MYCCONSENSUSAT	CANNTG	Motif found in the promoters of the dehydration-responsive gene rd22	Squamosa promoter-binding protein-like 12 (SPL12)
Ca-CNMS194	28366874	CaChr3	37116732	(TC)8	CATTTCCCCCTTCCATTTTT	CAGTGGGATTCAGAGTAGGGA	60.0	164	CTRMCAMV35S	TCTCTCTCT	CaMV 35S (Inverted GAGA) found in a 60-nucleotide region downstream of the transcription start site of the	Domain of unknown function DUF250
Ca-CNMS195	28366875	CaChr3	37211281	(TGA)4	CCTCAAATGATCCAAACTTTA	CCAATGATAGGAAGAAAAGGT	55.0	153	GTGANTG10	GTGA	Motif found in the promoter of the tobacco late pollen gene g10	Transcription factor interacting with photoreceptors phyA and phyB
Ca-CNMS196	28366876	CaChr3	37252246	(CAA)5	TCAACAACCCAGATACTTGATGA	TCGGGCCTTATTTCTTTTCA	59.5	175	RAV1AAT	CAACA	AP2 & B3 like domiane in RAV1	Peptidyl-prolyl cis-trans isomerase, cyclophilin-type
Ca-CNMS197	28366877	CaChr3	37786659	(GAC)4	TACATGTTCAGCACAGCTAGA	TCATCGTCATATTTGAAATCC	55.0	150	CGACGOSAMY3	CGACG	Amy3D & Amy 3E alpha-amylase gene	RING/U-box protein
Ca-CNMS198	28366878	CaChr3	38142568	(CT)6	CTTCTCCGAGTCCGACCAT	AAAATGGCAGGAAAGGAAGC	60.2	172	CTRMCAMV35S	TCTCTCTCT	CaMV 35S (Inverted GAGA) found in a 60-nucleotide region downstream of the transcription start site of the	Cystathionine beta-synthase, core
Ca-CNMS199	28366879	CaChr3	38224187	(AAAC)5	GCTGAGGAGAAATTTCGTGG	CGACTTTTCTTCTGCCATCTG	59.8	154	ANAERO1CONSENSUS	AAACAAA	Motif found in silico in promoters of 13 anaerobic genes involved in the fermentative pathway	Kinesin, motor domain
Ca-CNMS200	28366882	CaChr3	38614360	(ACCA)3	TCACGAGTTTTTGTTTATGGT	GAGAAATACGAGCCTAGGAAG	55.0	151	MYBPZM	CCWACC	Core of consensus maize P (myb homolog) binding site	DNA-binding storekeeper protein-related transcriptional regulator
Ca-CNMS201	28366883	CaChr3	38664907	(TTTAT)5	CCCCTTCATCAGTGGTGAGT	TCTTCTCCTCCCAAAAGCAA	60.0	257	TATABOX5	TTATTT	TATA box found in the 5'upstream region of pea glutamine synthetase gene	Protein of unknown function DUF868, plant
Ca-CNMS202	28366884	CaChr3	38875738	(CT)13	CGTTGCATTCCAAAGTGAGA	ATGCAATGATGCTTGGTTGA	59.8	180	CTRMCAMV35S	TCTCTCTCT	CaMV 35S (Inverted GAGA) found in a 60-nucleotide region downstream of the transcription start site of the	SSXT
Ca-CNMS203	28366885	CaChr3	39136075	(CAA)6	GCTGACCTTGAATTCTCCAAA	TGTTTTGCTTTCAGTCACCG	59.3	152	RAV1AAT	CAACA	AP2 & B3 like domiane in RAV1	HEAT
Ca-CNMS204	28366886	CaChr3	39288289	(TTAT)3	CCAACTTTTTAGTCTTTGATTTG	TCCTCAGAACCATAACTTTGA	55.0	143	TATABOX5	TTATTT	TATA box found in the 5'upstream region of pea glutamine synthetase gene	Basic domain leucine zipper (bZip) transcription factor bZIP11
Ca-CNMS205	28366887	CaChr3	39344383	(TTC)4	GAGAACTCAAAAGGAACCCTA	CAAAACCTACGTATTGTCGTC	55.0	140	POLLEN1LELAT52	AGAAA	One of two co-dependent regulatory elements responsible for pollen specific activation of tomato lat52	Acyl-CoA N-acyltransferases (NAT) superfamily protein
Ca-CNMS206	28366888	CaChr3	39462370	(TTTTAT)5	GTGTGCGAAAATGGAGGATT	CTCGTTGATACGACACTGTTCC	59.9	263	TATABOX5	TTATTT	TATA box found in the 5'upstream region of pea glutamine synthetase gene	Acid phosphatase/vanadium-dependent haloperoxidase-related
Ca-CNMS207	28366889	CaChr3	39572156	(AG)8	AAAAGAAAAGCGCATTCACG	CGAATTCGAACTTTTGATTATATG	60.4	260	GAGA8HVBKN3	GAGAGAGAGAGAGAGA	Motif found in intron IV of the barley (H.v.) gene Bkn3	Zinc finger, Dof-type
Ca-CNMS208	28366890	CaChr3	39742922	(CCATA)4	AAAACAACACTCTCCTCTTCC	TATCTTGAGAAGCAACGGATA	55.0	143	S1FBOXSORPS1L21	ATGGTA	"S1F box" conserved both in spinach RPS1 and RPL21 genes	Transcription elongation factor (TFIIS) family protein
Ca-CNMS209	28366891	CaChr3	39742922	(GA)16	AGGTTGGTTGTCTTAAGTTGA	TTTCTGCTTCACTTGATCTTC	54.0	149	GAGA8HVBKN3	(GA)8	Motif found in intron IV of the barley (H.v.) gene Bkn3	pseudo-response regulator
Ca-CNMS210	28366893	CaChr3	39780106	(AAG)6	ATGACAAATCTGAGTCTTTGG	GGCTTCATGTAGTTGTAGGTG	54.0	156	DOFCOREZM	AAAG	Site required for binding of Dof proteins in maize	LHY myb-related putative transcription factor
Ca-CNMS211	28366894	CaChr3	39816299	(AG)6	TTTGGATATATGAGGATGGTG	AAGTTTCACTTTTGTGCCTTA	55.0	177	CTRMCAMV35S	TCTCTCTCT	CaMV 35S (Inverted GAGA) found in a 60-nucleotide region downstream of the transcription start site of the	Duplicated homeodomain-like superfamily protein
Ca-CNMS212	28366895	CaChr3	39816299	(GAA)7	AGCAAAGAAGAAGAAGAGGAA	CTTCTTCTTTTGATGGTGTTG	55.0	143	POLLEN1LELAT52	AGAAA	One of two co-dependent regulatory elements responsible for pollen specific activation of tomato lat52	NGATHA3 (NGA3)
Ca-CNMS213	28366896	CaChr3	39816299	(AAGG)3	TCTCTTTTGAAGGAAGGAAAG	GATAGTTTGGAGGGAGAAGTG	55.0	151	DOFCOREZM	AAAG	Site required for binding of Dof proteins in maize	NGATHA3 (NGA3)

Marker IDs	NCBI Probe IDs (PUIDs)	Chromosomes	Physical positions (bp)	Microsatellite repeat-motifs	Forward Primer (5'-3')	Reverse Primer (5'-3')	Actual annealing tempearture (0C)	Size (bp) of alleles amplified	Regulatory elements	Signal sequences of known regulatory element-binding sites	Function of known regulatory elements/ transcription factor-binding sites	Putative gene function
Ca-CNMS214	28366897	CaChr4	2885242	(ATTT)6	TCATTTTGTCGCAGTTTTGTT	CGCCAACTCTGTTACCTAATTACA	58.3	191	TATABOX5	TTATTT	TATA box found in the 5'upstream region of pea glutamine synthetase gene	General substrate transporter
Ca-CNMS215	28366898	CaChr4	2897444	(TATT)6	AAATGCTGGTAAATTTATTGTTTACT T	GGGTTTCTTTGGTTTAGGAAA	57.3	278	TATABOX5	TTATTT	TATA box found in the 5'upstream region of pea glutamine synthetase gene	General substrate transporter
Ca-CNMS216	28366899	CaChr4	3057657	(GCC)5	ACCTTTTCCAAAGCCTCCAC	CATGGGATTGGAGGTGGTAT	60.5	264	GCCCORE	GCCGCC	Motif found in many pathogen-responsive genes such as PDF1.2, Thi2.1 and PR4	Tetratricopeptide TPR-1
Ca-CNMS217	28366900	CaChr4	3547650	(AG)9	CACCTGCAGTGAAAAGACCA	GATGGAACGACCGGAATATG	59.9	255	GAGA8HVBKN3	GAGAGAGAGAGAGAGA	Motif found in intron IV of the barley (H.v.) gene Bkn3	Methyltransferase type 11
Ca-CNMS218	28366901	CaChr4	3595591	(TC)7	TTCGTGAATTTGTGGTGAGC	TCTGATGCTTGTCGGTTCTG	59.7	123	CTRMCAMV35S	TCTCTCTCT	CaMV 35S (Inverted GAGA) found in a 60-nucleotide region downstream of the transcription start site of the	Domain of unknown function DUF231, plant
Ca-CNMS219	28366902	CaChr4	3603452	(TC)10	CAACGGTCACCTTTTTGGTT	GTTAGGGATTTGTCGGAGCA	59.9	217	CTRMCAMV35S	TCTCTCTCT	CaMV 35S (Inverted GAGA) found in a 60-nucleotide region downstream of the transcription start site of the	Microtubule-associated protein, MAP65/ASE1-type
Ca-CNMS220	28366904	CaChr4	3930103	(CT)12	TCCTCCCATTCTTTGCATTC	GAAGTGGTTTCGGAGCAAAA	60.0	149	CTRMCAMV35S	TCTCTCTCT	CaMV 35S (Inverted GAGA) found in a 60-nucleotide region downstream of the transcription start site of the	Inorganic pyrophosphatase
Ca-CNMS221	28366905	CaChr4	3969881	(CT)12	CGAAGCGAACTCTCATTTCC	GGGGGTGTAAGTAGCACTCG	60.0	124	CTRMCAMV35S	TCTCTCTCT	CaMV 35S (Inverted GAGA) found in a 60-nucleotide region downstream of the transcription start site of the	Protein of unknown function DUF789
Ca-CNMS222	28366906	CaChr4	4027434	(CT)9	GCAGCATGTTGGCTTCTGTA	CTGTGGGCACTGAGTTGTGT	60.0	259	CTRMCAMV35S	TCTCTCTCT	CaMV 35S (Inverted GAGA) found in a 60-nucleotide region downstream of the transcription start site of the	Zinc finger, Dof-type
Ca-CNMS223	28366907	CaChr4	4276255	(ATCT)3	TGTTGTAGATTTGTCTATGTTGC	AAGGATAAATTCGTGGTCCTA	55.0	148	GATABOX	GATA	GATA motif in CaMV 35S promoter required for high level, light regulated, and tissue specific expression	PKDM7D
Ca-CNMS224	28366908	CaChr4	4841461	(AG)12	TCAGAGTGGAAGAGTGCGTG	CTGAACTGAACGTAGAATGGAA	60.2	118	GAGA8HVBKN3	GAGAGAGAGAGAGAGA	Motif found in intron IV of the barley (H.v.) gene Bkn3	Glycosyl transferase, family 8
Ca-CNMS225	28366909	CaChr4	5843225	(TTG)6	TTTCGTGTAGACCATCTGTTTGA	GTCGAGGACCCATTGAACAT	59.7	273	CAREOSREP1	CAACTC	Motif found in REP-1 gene promoter	ATPase, AAA+ type, core
Ca-CNMS226	28366910	CaChr4	6324932	(ACA)6	AATGACGACAATAGCCTCCG	CAAGCAAAGGAATCTTGGTGA	60.1	104	RAV1AAT	CAACA	AP2 & B3 like domiane in RAV1	Glycosyl transferase, family 8
Ca-CNMS227	28366911	CaChr4	6543495	(TC)7	AAGCCACCCCTTCAAATTCT	ATGTGCCATGCTGCTTAGAG	59.9	149	CTRMCAMV35S	TCTCTCTCT	CaMV 35S (Inverted GAGA) found in a 60-nucleotide region downstream of the transcription start site of the	SANT domain, DNA binding
Ca-CNMS228	28366912	CaChr4	6752896	(TC)9	CCGGCCAAAGTAAATTCTTG	AGGGTGGTTCAAAGGGAGAG	59.6	189	CTRMCAMV35S	TCTCTCTCT	CaMV 35S (Inverted GAGA) found in a 60-nucleotide region downstream of the transcription start site of the	Pectinacetylesterase
Ca-CNMS229	28366913	CaChr4	6941575	(TTTTTA)3	TCTAACGAAAACTGGTTGAAA	TTTCAACCAGTTTTCGTTAGA	55.0	158	SEF4MOTIFGM7S	RTTTTTR	Motif found in 5'upstream region (-199) of beta-conglycinin (7S globulin) gene	Protein phosphatase 2C family protein
Ca-CNMS230	28366915	CaChr4	6941575	(TCT)5	TCGTGAATATTGCTTCCTTTA	CAAAATAGAAACTGCGAGAAA	55.0	149	NODCON2GM	CTCTT	Putative nodulin consensus sequences	Protein phosphatase 2C family protein
Ca-CNMS231	28366916	CaChr4	7120661	(GT)6	GAAGAATCAACAACATCCTCA	CAAAAAGCTGTGAAAAATAGC	55.0	161	CACTFTPPCA1	YACT	Tetranucleotide (CACT) is a key component of Mem1 found in the cis-regulatory element in the distal region of	WRKY Transcription Factor
Ca-CNMS232	28366917	CaChr4	7120661	(AGT)5	GTGGTGCAACGATAGTAACTC	CAAAAAGCTGTGAAAAATAGC	55.0	156	CACTFTPPCA1	YACT	Tetranucleotide (CACT) is a key component of Mem1 found in the cis-regulatory element in the distal region of	WRKY Transcription Factor
Ca-CNMS233	28366918	CaChr4	7548012	(GAGAAA)4	TGAAACTTCAGAGGTTGGTAA	TTCCTCTGTAGAGGTTTTTCC	55.0	144	POLLEN1LELAT52	AGAAA	One of two co-dependent regulatory elements responsible for pollen specific activation of tomato lat52	NAD(P)-binding Rossmann-fold superfamily protein
Ca-CNMS234	28366919	CaChr4	7834673	(AATAT)5	TGATCCAAGTCCCACACAAA	TTGCCCATTGTTGATTCTGA	59.9	247	ROOTMOTIFTAPOX1	ATATT	Motif found both in promoters of rolD	Universal stress protein A
Ca-CNMS235	28366920	CaChr4	7970746	(AG)10	CAGAAAGAAAAATACGAGCTG	CAGAACCCAGTTCTCATGTAA	55.0	136	GAGA8HVBKN3	(GA)8	Motif found in intron IV of the barley (H.v.) gene Bkn3	Zinc finger C-x8-C-x5-C-x3-H type family protein
Ca-CNMS236	28366921	CaChr4	8049371	(TATTT)3	GTGAAAGAGGGTCTTTATCTTG	ACATCGTCAAGAAGAAGACAA	55.0	150	TATABOX5	TTATTT	TATA box found in the 5'upstream region of pea glutamine synthetase gene	NAC transcription factor
Ca-CNMS237	28366922	CaChr4	8163137	(CT)6	AAGTGGGTCCCATATGAACAG	TGGTGAAGAGAGAGAAAGACAAA	58.8	127	CTRMCAMV35S	TCTCTCTCT	CaMV 35S (Inverted GAGA) found in a 60-nucleotide region downstream of the transcription start site of the	Lipase, GDXG, active site
Ca-CNMS238	28366923	CaChr4	8751606	(CAA)4	CTTTCAAACTTTGTTCCAATG	GTAAAGGGTGGTTTGAATTTT	55.0	145	RAV1AAT	CAACA	AP2 & B3 like domians in RAV1	SHI gene family protein
Ca-CNMS239	28366924	CaChr4	8997043	(AGAGAA)6	CCCAAACAGACCCTTACCAA	TTCACCTTTCTCTCTTAAACCCA	59.8	229	POLLEN1LELAT52	AGAAA	One of two co-dependent regulatory elements responsible for pollen specific activation of tomato lat52	EXTL2, alpha-1,4-N-acetylhexosaminyltransferase
Ca-CNMS240	28366926	CaChr4	9179530	(TC)7	CGAAACCATGCTATATGAACCA	TTCATATGATGATTGCTCAATGG	59.9	101	CTRMCAMV35S	TCTCTCTCT	CaMV 35S (Inverted GAGA) found in a 60-nucleotide region downstream of the transcription start site of the	Glycosyl transferase, family 2
Ca-CNMS241	28366927	CaChr4	9449939	(CT)7	ATTTTGATTCTCCTCAGATCC	GAAATCTTTTGCTGCTTATGA	55.0	145	CTRMCAMV35S	TCTCTCTCT	CaMV 35S (Inverted GAGA) found in a 60-nucleotide region downstream of the transcription start site of the	Myb-like transcription factor family protein
Ca-CNMS242	28366928	CaChr4	9449939	(TTCT)3	ATTTTGATTCTCCTCAGATCC	AGGGTAGAAAACATGGAAAAG	55.0	145	-10PEHVPSBD	TATTCT	Motif found in the barley (H.v.) chloroplast psbD gene promoter	Myb-like transcription factor family protein
Ca-CNMS243	28366929	CaChr4	9470904	(AGTT)3	CGTGTGTCTCTAACCATCTTC	CTACCTCCTTCCATTTTCTGT	55.0	150	MYB1LEPR	GTTAGTT	Tomato Pti4(ERF) regulates defence-related gene expression via GCC box and non-GCC box cis elements	RAV transcription factor family protein containing AP2 and B3
Ca-CNMS244	28366930	CaChr4	9470904	(GTTA)3	CGTGTGTCTCTAACCATCTTC	CTACCTCCTTCCATTTTCTGT	55.0	150	MYB1LEPR	GTTAGTT	Tomato Pti4(ERF) regulates defence-related gene expression via GCC box and non-GCC box cis elements	RAV transcription factor family protein containing AP2 and B3
Ca-CNMS245	28366931	CaChr4	9736203	(TC)11	CACTTCACCCACCTTCCCTA	ATCCAACAATCTGGGGACAA	60.0	256	CTRMCAMV35S	TCTCTCTCT	CaMV 35S (Inverted GAGA) found in a 60-nucleotide region downstream of the transcription start site of the	Peptidyl-prolyl cis-trans isomerase, cyclophilin-type
Ca-CNMS246	28366932	CaChr4	9973444	(AG)6	CGTGATAATTATTCGCTGCTTT	TGGTTCGAAAAGAAACAAAAGTG	58.5	240	CTRMCAMV35S	TCTCTCTCT	CaMV 35S (Inverted GAGA) found in a 60-nucleotide region downstream of the transcription start site of the	Amino acid transporter, transmembrane
Ca-CNMS247	28366933	CaChr4	10910652	(CCT)6	GCTGCGCCGGCTACTTTA	TCTGCTGCATGCGTTTAACT	62.5	179	RAV1AAT	CAACA	AP2 & B3 like domiane in RAV1	Protein-tyrosine phosphatase, receptor/non-receptor type
Ca-CNMS248	28366934	CaChr4	10934694	(AAC)5	CCCTATTAGCAATGCTCCCA	GGTGATAGTGGTGGTGGAGG	60.1	256	RAV1AAT	CAACA	AP2 & B3 like domiane in RAV1	Exostosin-like
Ca-CNMS249	28366935	CaChr4	11000591	(TATT)5	TGTGCGTTAGAATTTCCGAT	TGGCCATGATATGACAACTTC	58.2	280	TATABOX5	TTATTT	TATA box found in the 5'upstream region of pea glutamine synthetase gene	Aminotransferase-like, plant mobile domain
Ca-CNMS250	28366937	CaChr4	11081915	(TAG)5	GGAAGGGAAATTCACACCAG	GTTGGGGTGGCATTGATAAG	59.4	117	CACTFTPPCA1	YACT	Tetranucleotide (CACT) is a key component of Mem1 found in the cis-regulatory element in the distal region of	Pseudouridine synthase I, TruA
Ca-CNMS251	28366938	CaChr4	12175382	(TGT)7	GATGGGTAATGGGTTTGTGG	CCTTCACAATTTCAATTTCAACA	59.9	235	CAREOSREP1	CAACTC	Motif found in REP-1 gene promoter	Transcription factor GRAS
Ca-CNMS252	28366939	CaChr4	12521142	(CT)8	TGGAATTCTTCCAACAAGGC	TGCAGAACAAGGAGAAACACA	60.1	127	CTRMCAMV35S	TCTCTCTCT	CaMV 35S (Inverted GAGA) found in a 60-nucleotide region downstream of the transcription start site of the	Cytochrome b561, eukaryote
Ca-CNMS253	28366940	CaChr4	12930785	(AG)9	CTTGGGAGTGAGTGCAACAA	GTGAAGAAGCGAGAAAAGCG	59.9	259	GAGA8HVBKN3	GAGAGAGAGAGAGAGA	Motif found in intron IV of the barley (H.v.) gene Bkn3	Oxoglutarate/iron-dependent oxygenase
Ca-CNMS254	28366941	CaChr4	13328534	(TCAC)4	CAAACCCAAACGCAAACA	CACGAAAACCAGTATCTTCAG	59.0	151	CACTFTPPCA1	YACT	Tetranucleotide (CACT) is a key component of Mem1 found in the cis-regulatory element in the distal region of	PPD2 (and its paralog, PPD1) encode plant-specific putative
Ca-CNMS255	28366942	CaChr4	14270540	(AGC)5	GCTGCCCATGTTCCTTTTTA	AATCTGGATCGGGGAAAATC	60.1	114	ANAERO2CONSENSUS	AGCAGC	Motif found in silico in promoters of 13 anaerobic genes involved in the fermentative pathway	Transcription factor, MADS-box
Ca-CNMS256	28366943	CaChr4	14679577	(GA)6	TTGCAGTGTATATTTGTTCCTTTTT	TCTCATCCTCAACCCTGGTC	58.3	135	GAGA8HVBKN3	GAGAGAGAGAGAGAGA	Motif found in intron IV of the barley (H.v.) gene Bkn3	Mpv17/PMP22

Marker IDs	NCBI Probe IDs (PUIDs)	Chromosomes	Physical positions (bp)	Microsatellite repeat-motifs	Forward Primer (5'-3')	Reverse Primer (5'-3')	Actual annealing tempearture (0C)	Size (bp) of alleles amplified	Regulatory elements	Signal sequences of known regulatory element-binding sites	Function of known regulatory elements/ transcription factor-binding sites	Putative gene function
Ca-CNMS257	28366944	CaChr4	14733634	(TATT)6	CCAAAACTGAGACACGCTGA	TTGTTGGATTGGTGTTTTCG	60.0	270	TATABOX5	TTATTT	TATA box found in the 5'upstream region of pea glutamine synthetase gene	Paired amphipathic helix
Ca-CNMS258	28366945	CaChr4	14810569	(TC)16	TCTCACCAACACCAACACAAA	ATTATGCGAAGCAACGGAAA	60.0	270	CTRMCAMV35S	TCTCTCTCT	CaMV 35S (Inverted GAGA) found in a 60-nucleotide region downstream of the transcription start site of the	Homeobox
Ca-CNMS259	28366946	CaChr4	15267674	(TC)7	CGAGCAAAAATCGCATCTCT	TGAAGTGAATGAATGAAAATGAAA	60.5	280	CTRMCAMV35S	TCTCTCTCT	CaMV 35S (Inverted GAGA) found in a 60-nucleotide region downstream of the transcription start site of the	Zinc finger, RING-CH-type
Ca-CNMS260	28366948	CaChr4	15375211	(AAC)5	CACAACAGCAATATGACCATGA	TTTCCACGTTGTGTTTTCTTTT	59.5	227	RAV1AAT	CAACA	AP2 & B3 like domiane in RAV1	ATPase, alpha/beta subunit, nucleotide-binding domain
Ca-CNMS261	28366949	CaChr4	15918341	(TC)8	AGCCCCTTCATTTCCTCATT	GGATGTGTCTGAAGCGGTTT	59.9	264	CTRMCAMV35S	TCTCTCTCT	CaMV 35S (Inverted GAGA) found in a 60-nucleotide region downstream of the transcription start site of the	Protein kinase, catalytic domain
Ca-CNMS262	28366950	CaChr4	15955236	(TC)6	TCCCTTTTGTGTGGACCTTC	CGATTATTGACAAATGAATGGG	59.9	134	CTRMCAMV35S	TCTCTCTCT	CaMV 35S (Inverted GAGA) found in a 60-nucleotide region downstream of the transcription start site of the	Zinc finger, C2H2-type
Ca-CNMS263	28366951	CaChr4	16532088	(AG)11	ATCTGCCATGCCTGAAGAAG	ATGTCGCTGTTGTTGCAAAT	60.4	225	GAGA8HVBKN3	GAGAGAGAGAGAGAGA	Motif found in intron IV of the barley (H.v.) gene Bkn3	Basic-leucine zipper (bZIP) transcription factor
Ca-CNMS264	28366952	CaChr4	16649921	(ACTTGC)4	TGAACTCGTATCTTCTGCTTC	ATCATGCTTCACAACTTCATC	55.0	172	EBOXBNNAPA	CANNTG	E-box of napA storage-protein gene of Brassica napus	Ovate family protein 13 (OFP13)
Ca-CNMS265	28366953	CaChr4	17074155	(TCT)4	TCCTATATTTTCCATCCTCCT	GCAGTATGTGTTTGTGAATCC	54.0	146	NODCON2GM	CTCTT	One of two putative nodulin consensus sequences	Basic helix-loop helix transcription factor
Ca-CNMS266	28366954	CaChr4	17195718	(TC)13	CCTCAACCATGGCCTGTACT	AGTTGCATTGGAGACAAGGG	60.0	118	CTRMCAMV35S	TCTCTCTCT	CaMV 35S (Inverted GAGA) found in a 60-nucleotide region downstream of the transcription start site of the	Domain of unknown function DUF632
Ca-CNMS267	28366955	CaChr4	18222741	(CATG)3	AAAGTTTAGGAGATTTTACTTGC	TGTCATCGTCACTCATTACAA	53.0	159	RYREPEATLEGUMEINB OX	CATGCAY	Legume box found in seed stroage gene in legume	SET domain-containing protein
Ca-CNMS268	28366956	CaChr4	18901090	(CT)7	GGAAGGAAAGGGTGTTAGCC	TTGTATGCAAGAGAGAGATAGAGAGA	59.9	150	CTRMCAMV35S	TCTCTCTCT	CaMV 35S (Inverted GAGA) found in a 60-nucleotide region downstream of the transcription start site of the	Protein kinase, catalytic domain
Ca-CNMS269	28366957	CaChr4	19060784	(AG)9	GGGCTACAGCAACGAAAGAG	TTAGGTTGGTGAGTTCCTCCA	60.0	197	GAGA8HVBKN3	GAGAGAGAGAGAGAGA	Motif found in intron IV of the barley (H.v.) gene Bkn3	Lipase, class 3
Ca-CNMS270	28366959	CaChr4	19886962	(AG)6	GTGAAATGTGCTTGTGGTGG	TGCAAAGAGAGCTTCCAAGT	60.0	244	CTRMCAMV35S	TCTCTCTCT	CaMV 35S (Inverted GAGA) found in a 60-nucleotide region downstream of the transcription start site of the	Zinc finger, C2H2-type
Ca-CNMS271	28366960	CaChr4	20408852	(CT)7	GTGTCGTGCGTTCTTCGTAA	TGATCGGAAAAAGGTTCAGC	59.9	176	CTRMCAMV35S	TCTCTCTCT	CaMV 35S (Inverted GAGA) found in a 60-nucleotide region downstream of the transcription start site of the	Protein kinase, catalytic domain
Ca-CNMS272	28366961	CaChr4	20435812	(ATTCT)4	GGGTCATCATCAAAACCA	AGTTCTGAAGTCATGGTAGGG	55.0	147	CAATBOX1	CAAT	CAAT promoter consensus sequence found in legA gene of pea	TCP family transcription factor
Ca-CNMS273	28366962	CaChr4	20762489	(AG)8	TGCAGTCATACGTCCAGTCA	CCTGTTGCTGTCACATTTGG	58.8	194	GAGA8HVBKN3	GAGAGAGAGAGAGAGA	Motif found in intron IV of the barley (H.v.) gene Bkn3	Exoribonuclease, phosphorolytic domain 1
Ca-CNMS274	28366963	CaChr4	21094058	(TC)6	CCTCGCAAACTCTGTTCCTC	AGAGGTTGAGCGAACCGTAA	60.0	246	CTRMCAMV35S	TCTCTCTCT	CaMV 35S (Inverted GAGA) found in a 60-nucleotide region downstream of the transcription start site of the	Peptidase A1
Ca-CNMS275	28366964	CaChr4	21094058	(CT)9	CGCTCAAACACTTTCAGCAA	GAACAGAGTTTGCGAGGGAG	60.2	118	CTRMCAMV35S	TCTCTCTCT	CaMV 35S (Inverted GAGA) found in a 60-nucleotide region downstream of the transcription start site of the	Peptidase A1
Ca-CNMS276	28366965	CaChr4	21711254	(TC)6	CTCTCTCTCCCTCACACACAA	TGCATGCGTTTATATCTTTGG	58.0	166	CTRMCAMV35S	TCTCTCTCT	CaMV 35S (Inverted GAGA) found in a 60-nucleotide region downstream of the transcription start site of the	Serine/threonine dehydratase, pyridoxal-phosphate-binding site
Ca-CNMS277	28366966	CaChr4	22009970	(TC)6	GGGGTGTTTTTAGGCACTGA	GCAATGAGAGTGCAAAGCAG	60.0	214	CTRMCAMV35S	TCTCTCTCT	CaMV 35S (Inverted GAGA) found in a 60-nucleotide region downstream of the transcription start site of the	Glycosyl transferase, family 2
Ca-CNMS278	28366967	CaChr4	22194464	(TTG)4	TGTTCCGTTCACTAGTTGTCT	CGCTTCTTCTCTCTCTCTCTC	55.0	169	RAV1AAT	CAACA	AP2 & B3 like domians in RAV1	Embryo sac development arrest 31 (EDA31)
Ca-CNMS279	28366968	CaChr4	22194464	(AG)8	CATGATTGTTATGGTGGTCTT	GTCAACAACTTCAATTTCCAA	55.0	150	GAGA8HVBKN3	(GA)8	Motif found in intron IV of the barley (H.v.) gene Bkn3	Embryo sac development arrest 31 (EDA31)
Ca-CNMS280	28366970	CaChr4	24207463	(TGT)5	AGCAGAGAATGCAGAACTTGC	TGTCCTAAATGCAAAAGCACTG	59.8	128	CAREOSREP1	CAACTC	Motif found in REP-1 gene promoter	Pentatricopeptide repeat
Ca-CNMS281	28366971	CaChr4	24589917	(TC)7	TCACCCTCATTTATCATCATC	ATCTTTCTCCTTTGCTCTCTC	55.0	142	CTRMCAMV35S	TCTCTCTCT	CaMV 35S (Inverted GAGA) found in a 60-nucleotide region downstream of the transcription start site of the	TEOSINTE BRANCHED 1, cycloidea and PCF transcription
Ca-CNMS282	28366972	CaChr4	24589917	(AAG)5	CATCCAAGACAAAGTTCCTTA	CTCTGGAAATTTAAACAAGCA	55.0	156	UPRMOTIFIIAT	CC(N)10CCACG	Motif II in conseved UPR element in SAR1B, HSP90, Ca-Atase etc genes	Paralog of NPR1
Ca-CNMS283	28366973	CaChr4	24751774	(GA)6	TGCCACAGAAATCAAATTGC	CCGAGGAAGTTGGTCACATT	59.7	119	CTRMCAMV35S	TCTCTCTCT	CaMV 35S (Inverted GAGA) found in a 60-nucleotide region downstream of the transcription start site of the	Protein of unknown function DUF3223
Ca-CNMS284	28366974	CaChr4	25713561	(AGAA)3	GATGAGAAAATCCAAGAGGTC	GAGTCTCTCAATTCTCTTTTGG	55.0	150	POLLEN1LELAT52	AGAAA	Motif found in the promoter of tomato endo-beta-mannanase gene	GATA factor family of zinc finger transcription factors
Ca-CNMS285	28366975	CaChr4	25837207	(TC)6	TTTCCTTTCCTTTCGTGGTC	AACATGCATATGGTTGCGTG	59.1	228	CTRMCAMV35S	TCTCTCTCT	CaMV 35S (Inverted GAGA) found in a 60-nucleotide region downstream of the transcription start site of the	Ankyrin repeat
Ca-CNMS286	28366976	CaChr4	27787647	(GA)6	TCCACTTTCGCCTCTTAACAA	GTTACAAGGATCCTCCCACG	59.9	222	CTRMCAMV35S	TCTCTCTCT	CaMV 35S (Inverted GAGA) found in a 60-nucleotide region downstream of the transcription start site of the	MATH
Ca-CNMS287	28366977	CaChr4	28855433	(ATTT)6	TCGGATTCTTTCAATCCCTC	TTTCACATCATTTTGAAAGTTTATCAT	59.1	279	TATABOX5	TTATTT	TATA box found in the 5'upstream region of pea glutamine synthetase gene	Protein kinase, catalytic domain
Ca-CNMS288	28366978	CaChr4	29471530	(CT)9	AAAGACTGGTCCATTCGGTG	CGAGAGAGGACGAACAAGAGA	60.0	229	CTRMCAMV35S	TCTCTCTCT	CaMV 35S (Inverted GAGA) found in a 60-nucleotide region downstream of the transcription start site of the	RNA recognition motif domain
Ca-CNMS289	28366979	CaChr4	30259876	(TTTG)3	TTCAGAAACGAGAGCAGACTA	AGGGTGAAAGTGAGATCTTTT	56.0	161	ANAERO1CONSENSUS	AAACAAA	Motif found in silico in promoters of 13 anaerobic genes involved in the fermentative pathway	SBP-box gene, a the SPL gene family
Ca-CNMS290	28366981	CaChr4	30848240	(TC)11	CAAACAAAAGAATCGAGGGC	CAAATAAAGCGGTTCTTTCAGG	59.7	202	CTRMCAMV35S	TCTCTCTCT	CaMV 35S (Inverted GAGA) found in a 60-nucleotide region downstream of the transcription start site of the	Basic-leucine zipper (bZIP) transcription factor
Ca-CNMS291	28366982	CaChr4	31867313	(GTT)5	CAAAGGCAAAAACAAGCCAT	TTACCAATGGAAGCAGCCAG	60.1	208	CAREOSREP1	CAACTC	Motif found in REP-1 gene promoter	Protein phosphatase 2C-like
Ca-CNMS292	28366983	CaChr4	32520743	(CAG)5	TCACACATGACACTTCACAACC	TGCATCAATGTGAAAAACAGC	59.5	271	ANAERO2CONSENSUS	AGCAGC	Motif found in silico in promoters of 13 anaerobic genes involved in the fermentative pathway	Integrase, catalytic core
Ca-CNMS293	28366984	CaChr4	32857973	(CGT)5	TGGAAGATGGTTTATCCGCT	ATCTGAGCCGAAGATTGAGG	59.5	101	CGACGOSAMY3	CGACG	Amy3D & Amy 3E alpha-amylase gene	DNA topoisomerase I, C-terminal
Ca-CNMS294	28366985	CaChr4	33429866	(CT)7	GATTTTCTCACCACATGCCC	TGCGTTGAATGAGTGGAAAG	60.3	120	CTRMCAMV35S	TCTCTCTCT	CaMV 35S (Inverted GAGA) found in a 60-nucleotide region downstream of the transcription start site of the	Protein of unknown function DUF1950
Ca-CNMS295	28366986	CaChr4	33609468	(AGAT)9	CCCTTTGGAAAGAGAGGAGG	AAGCCGATTCTTGGGACTTT	60.2	190	GATABOX	GATA	GATA motif in CaMV 35S promoter required for high level, light regulated, and tissue specific expression	Putative esterase
Ca-CNMS296	28366987	CaChr4	35565029	(CT)6	TCTTCACCTTCTTTTCCCCA	GTTCGAGCCATGGTTGTTTT	59.6	115	CTRMCAMV35S	TCTCTCTCT	CaMV 35S (Inverted GAGA) found in a 60-nucleotide region downstream of the transcription start site of the	Drug/metabolite transporter
Ca-CNMS297	28366988	CaChr4	36799272	(ATCGG)3	GCCGATCTTAAAGTAGAATGG	ACAAACAAATAGACCTCCACA	56.0	152	ARR1AT	NGATT	Motif found in the promoter of rice NSHB gene	U2 auxiliary factor small subunit
Ca-CNMS298	28366989	CaChr4	37022034	(TC)6	TTCCCCAAAAGCTGGACTAA	TGCAGACTGTTTGGTTCGAG	59.7	114	CTRMCAMV35S	TCTCTCTCT	CaMV 35S (Inverted GAGA) found in a 60-nucleotide region downstream of the transcription start site of the	SET domain
Ca-CNMS299	28366990	CaChr4	37150143	(GA)17	GCATTTCCCACAATCAACCT	CTTGATCACAATGGCCACC	59.8	279	GAGA8HVBKN3	GAGAGAGAGAGAGAGA	Motif found in intron IV of the barley (H.v.) gene Bkn3	Glycosyl transferase, family 20

Marker IDs	NCBI Probe IDs (PUIDs)	Chromosomes	Physical positions (bp)	Microsatellite repeat-motifs	Forward Primer (5'-3')	Reverse Primer (5'-3')	Actual annealing tempearture (0C)	Size (bp) of alleles amplified	Regulatory elements	Signal sequences of known regulatory element-binding sites	Function of known regulatory elements/ transcription factor-binding sites	Putative gene function
Ca-CNMS300	28366993	CaChr4	37738902	(AG)7	ACGTTTTAGTCGACACACATTTT	AGCGATCCTAATTCATCCAAA	57.8	270	GAGA8HVBKN3	GAGAGAGAGAGAGAGA	Motif found in intron IV of the barley (H.v.) gene Bkn3	C2 calcium-dependent membrane targeting
Ca-CNMS301	28366994	CaChr4	37751515	(TC)8	GCGTGGAGTGAAAGAGGAAG	AGCACCTTGCATCACGAATA	60.0	250	CTRMCAMV35S	TCTCTCTCT	CaMV 35S (Inverted GAGA) found in a 60-nucleotide region downstream of the transcription start site of the	Aminotransferase-like, plant mobile domain
Ca-CNMS302	28366995	CaChr4	37857050	(AT)13	TCCCTGTATATTACAATCCTCGG	CATGAAAGTGGTGGAAATTTGA	59.3	134	CAATBOX1	CAAT	CAAT promoter consensus sequence found in legA gene of pea	Auxin efflux carrier
Ca-CNMS303	28366996	CaChr4	38308266	(GAAACA)3	TAGTAGAATGCGTTCACGTTT	GAAAGACCAACAAAGCTTAGAC	55.0	151	POLLEN1LELAT52	AGAAA	One of two co-dependent regulatory elements responsible for pollen specific activation of tomato lat52	BTB/POZ domain-containing protein
Ca-CNMS304	28366997	CaChr4	38419862	(AGA)4	TTGAGAAGCACTCAAAGAAAG	CAACACGGAATATACGTTCTC	55.0	132	SORLIP3AT	CTCAAGTGA	Sequences over-represented in light-Induced Promoters	zinc finger (CCCH-type) family protein
Ca-CNMS305	28366998	CaChr4	38679878	(TGC)5	ACCTGTGGCTCCATTTTCAC	TGGGCAATCGAATGTGTCTA	60.0	261	ANAERO2CONSENSUS	AGCAGC	Motif found in silico in promoters of 13 anaerobic genes involved in the fermentative pathway	LIM binding protein
Ca-CNMS306	28366999	CaChr4	39659420	(AGC)5	GCTGCCCATGTTCCTTTTTA	AATCTGGATCGGGGAAAATC	60.1	114	ANAERO2CONSENSUS	AGCAGC	Motif found in silico in promoters of 13 anaerobic genes involved in the fermentative pathway	Protein kinase, catalytic domain
Ca-CNMS307	28367000	CaChr4	39796646	(TTC)4	TTTCAAACAACACACCACAC	GATAAATCCACAATTCGCATA	55.0	150	CYTOSITECSHPRA	AAGATTGATTGAG	Motif found in cucumber hydroxypyruvate reductase (hprA) gene promoter	Putative c-myb-like transcription factor
Ca-CNMS308	28367001	CaChr4	40587934	(TC)6	GCTTTTGTCAGTTTCGGACC	CGAGGTTGAGGTTGTCTCGT	59.7	180	CTRMCAMV35S	TCTCTCTCT	CaMV 35S (Inverted GAGA) found in a 60-nucleotide region downstream of the transcription start site of the	Zinc finger, RING-type
Ca-CNMS309	28367002	CaChr4	41181947	(CT)14	GCATTTCTCTGCCTCCTGTC	TGAAGAATGGGGTAGATGGG	60.0	145	CTRMCAMV35S	TCTCTCTCT	CaMV 35S (Inverted GAGA) found in a 60-nucleotide region downstream of the transcription start site of the	Exo70 exocyst complex subunit
Ca-CNMS310	28367004	CaChr4	41986507	(AG)6	TTTTTCTTCTCACTTCGATCA	TAGAAACGAAAGCTGAAAAGC	55.0	127	CTRMCAMV35S	TCTCTCTCT	CaMV 35S (Inverted GAGA) found in a 60-nucleotide region downstream of the transcription start site of the	HTA9, a histone H2A protein
Ca-CNMS311	28367005	CaChr4	42349161	(TGA)4	AAATTGAGCATGAATGGTATG	CCTCTCACTATAACCCAACAA	55.0	173	GTGANTG10	GTGA	Motif found in the promoter of the tobacco late pollen gene g10	Transcription factor of the B-ZIP family
Ca-CNMS312	28367006	CaChr4	42773320	(GATT)3	GGAGTTAAGTCGAGTTGATAGAA	ACAACCACTTCCATTTACACA	55.0	156	ARR1AT	NGATT	found in the promoter of rice NSHB gene	Acyl-CoA N-acyltransferases (NAT) superfamily protein
Ca-CNMS313	28367007	CaChr4	43641336	(TTTA)5	TGGGTCAACATGGAATTGTG	TGGTTTTATACGATGGACATTTCTT	60.2	270	TATABOX5	TTATTT	TATA box found in the 5'upstream region of pea glutamine synthetase gene	Pathogenesis-related transcriptional factor/ERF, DNA-
Ca-CNMS314	28367008	CaChr4	43926856	(CT)11	TTCAGCAAACACTCAGGCAC	GCACTGATCAGGTGGCAGTA	60.0	106	CTRMCAMV35S	TCTCTCTCT	CaMV 35S (Inverted GAGA) found in a 60-nucleotide region downstream of the transcription start site of the	Brix domain
Ca-CNMS315	28367009	CaChr4	44393274	(TTGA)3	ATCTCTTCATCAGTTGGATGG	TGTTATTTCTTCTTTCATCACTG	57.0	150	ARR1AT	NGATT	Motif found in the promoter of rice NSHB gene	Arabidopsis NAC domain containing protein 87 (ANAC087)
Ca-CNMS316	28367010	CaChr4	44655063	(AG)10	GGACACACAACAGAGAGAAAA	ACGAGGATTGTAAGGAGTACC	55.0	167	GAGA8HVBKN3	(GA)8	Motif found in intron IV of the barley (H.v.) gene Bkn3	AL4 the Alfin-Like family of nuclear-localized PhD domain
Ca-CNMS317	28367011	CaChr4	45108427	(TC)7	ATTTCCCTGTTTCCAGGCTC	TTGTGTGTGTTGGTGATGAAGA	60.4	149	CTRMCAMV35S	TCTCTCTCT	CaMV 35S (Inverted GAGA) found in a 60-nucleotide region downstream of the transcription start site of the	Glycine cleavage system P-protein, N-terminal
Ca-CNMS318	28367012	CaChr4	45183588	(TC)8	GGCCCTCTAAAGCCTGAAAC	AACAAAAAGCATTGAAGAGCG	60.2	245	CTRMCAMV35S	TCTCTCTCT	CaMV 35S (Inverted GAGA) found in a 60-nucleotide region downstream of the transcription start site of the	Homeobox
Ca-CNMS319	28367013	CaChr4	45415634	(TTTC)4	CTCGTCTATGCTGTATTAGGC	CGGTGGACACTCACTACTACT	54.0	150	POLLENILELAT52	AGAAA	Motif found in the promoter of tomato endo-beta-mannanase gene	Floral homeotic gene, an AP2/EREBP protein
Ca-CNMS320	28367015	CaChr4	45807790	(TTG)6	GACGAATTGGTTGAGGTGCT	TCATCGAATTGGGGTCCTTA	60.1	275	CAREOSREP1	CAACTC	Motif found in REP-1 gene promoter	F-box domain, cyclin-like
Ca-CNMS321	28367016	CaChr4	47236389	(GTT)5	CGGGCAAAGAGAGTGAAAAG	TCCCAAACCTACCACTTTCG	60.0	229	CAREOSREP1	CAACTC	Motif found in REP-1 gene promoter	Transcriptional factor B3
Ca-CNMS322	28367017	CaChr4	48200196	(CT)6	AGGGAAGAGGAATAAAGTTTG	GATCTGCAAGAATGTGAAAAG	54.0	135	CTRMCAMV35S	TCTCTCTCT	CaMV 35S (Inverted GAGA) found in a 60-nucleotide region downstream of the transcription start site of the	Auxin response factor
Ca-CNMS323	28367018	CaChr4	48200196	(TTC)4	GATGGTGAAAAGAGGAGTGTT	CTCTCTAAGTAGCGGAAGAGG	56.0	152	POLLEN1LELAT52	AGAAA	One of two co-dependent regulatory elements responsible for pollen specific activation of tomato lat52	SHI gene family protein
Ca-CNMS324	28367019	CaChr4	48200620	(AG)7	TTTCACGAAAGTTCCACTTACC	GGTCAGTGACGAATTTTCAAAC	58.3	224	CTRMCAMV35S	TCTCTCTCT	CaMV 35S (Inverted GAGA) found in a 60-nucleotide region downstream of the transcription start site of the	Protein of unknown function DUF702
Ca-CNMS325	28367020	CaChr4	48267284	(CT)10	AAGACCGTTGCTCATTACC	TTTCAACTTTTTCACCATCAG	55.0	151	CTRMCAMV35S35S	TCTCTCTCT	CaMV 35S (Inverted GAGA) found in a 60-nucleotide region downstream of the transcription start site of the	LSD1-like 3 (LDL3)
Ca-CNMS326	28367021	CaChr4	48319743	(AAC)4	TTCCCATTCAGTCATTTAATC	CTTTCGTCAATTATTTGCAGT	54.0	146	RAV1AAT	CAACA	AP2 & B3 like domiane in RAV1	Basic-leucine zipper (bZIP) transcription factor family protein
Ca-CNMS327	28367022	CaChr4	48319743	(AATC)3	TTCCCATTCAGTCATTTAATC	CAAGTCTTTGTCATCTTTGCT	54.0	146	CAATBOX1	CAAT	CAAT promoter consensus sequence found in legA gene of pea	Basic-leucine zipper (bZIP) transcription factor family protein
Ca-CNMS328	28367023	CaChr4	48319743	(ACT)4	TTCCCATTCAGTCATTTAATC	CTTTCGTCAATTATTTGCAGT	54.0	146	CACTFTPPCA1	YACT	Tetranucleotide (CACT) is a key component of Mem1 found in the cis-regulatory element in the distal region of	Basic-leucine zipper (bZIP) transcription factor family protein
Ca-CNMS329	28367024	CaChr4	49040318	(AAAG)9	GTGGTAGATCTGGGGAATGC	AGTTTGGACGTCCCCTTTCT	59.4	110	POLLEN1LELAT52	AGAAA	One of two co-dependent regulatory elements responsible for pollen specific activation of tomato lat52	Multicopper oxidase, type 1
Ca-CNMS330	28367026	CaChr5	1444315	(AG)12	GTGATGGTTGGTGAGCAATG	CGGAGTTCTGGGACATGTTT	60.0	225	GAGA8HVBKN3	GAGAGAGAGAGAGAGA	Motif found in intron IV of the barley (H.v.) gene Bkn3	Recoverin
Ca-CNMS331	28367027	CaChr5	2062842	(ATTG)5	ATTGCTTTGGCATGTTAGCC	AAAGGCAAAAGGATGGGAGT	60.1	149	ARR1AT	NGATT	Motif found in the promoter of rice NSHB gene	4-Diphosphocytidyl-2C-methyl-D-erythritol synthase
Ca-CNMS332	28367028	CaChr5	2221812	(AAGCA)5	AGCCATGTTTTGTCCTCACC	CGGCACAAGAAAGGTGTTTT	60.0	190	DOFCOREZM	AAAG	Site required for binding of Dof proteins in maize	ZF-HD homeobox protein, Cys/His-rich dimerisation domain
Ca-CNMS333	28367029	CaChr5	2231812	(CTTTG)5	AATGGAGAAAAAGCAGAAGAC	GGTTTGGATGCATAACAATAA	55.0	144	NODCON2GM	CTCTT	putative nodulin consensus sequences	Homeobox protein 31 (HB31)
Ca-CNMS334	28367030	CaChr5	7654298	(GTG)5	TTTGATGGGGAGGAGTTGAG	CAAATAAAAACGACGCCCAC	60.0	127	CAREOSREP1	CAACTC	Motif found in REP-1 gene promoter	Regulator of nonsense-mediated decay, UPF3
Ca-CNMS335	28367031	CaChr5	9956299	(TC)14	GTGGAGCTCCCTTGTTGTGT	TCAAGCAAACAAGGTGAAACA	60.2	156	CTRMCAMV35S	TCTCTCTCT	CaMV 35S (Inverted GAGA) found in a 60-nucleotide region downstream of the transcription start site of the	DNA-binding WRKY
Ca-CNMS336	28367032	CaChr5	15397394	(TC)9	AAGATTAAATTTGTGCGGGC	TTGTTATTGGTGCGTTCGAT	59.1	158	CTRMCAMV35S	TCTCTCTCT	CaMV 35S (Inverted GAGA) found in a 60-nucleotide region downstream of the transcription start site of the	Haloacid dehalogenase-like hydrolase
Ca-CNMS337	28367033	CaChr5	16235206	(TC)6	GAAGCAAAATAGTTCCTGTTCCA	AACGGATTGGAGGGGGAC	59.7	176	CTRMCAMV35S	TCTCTCTCT	CaMV 35S (Inverted GAGA) found in a 60-nucleotide region downstream of the transcription start site of the	SANT domain, DNA binding
Ca-CNMS338	28367034	CaChr5	16675359	(ATTTCA)3	CCTAAATTCCTCTTCATTTCA	CGAGATCTGGTTGAGAAGAG	54.0	147	CAATBOX1	CAAT	CAAT promoter consensus sequence found in legA gene of pea	CCCH-type zinc finger protein with ARM repeat domain
Ca-CNMS339	28367035	CaChr5	16675359	(TC)9	TTTCAATTTCGAAGACGTTAC	CTGATCAATCTGAATGGAAAA	55.0	146	CTRMCAMV35S	TCTCTCTCT	CaMV 35S (Inverted GAGA) found in a 60-nucleotide region downstream of the transcription start site of the	CCCH-type zinc finger protein with ARM repeat domain
Ca-CNMS340	28367037	CaChr5	16762175	(CT)8	TTCCGAAAAACTCATTTTCTGTT	AAAATGCGGTAACAGGATGG	59.2	202	CTRMCAMV35S	TCTCTCTCT	CaMV 35S (Inverted GAGA) found in a 60-nucleotide region downstream of the transcription start site of the	Pentatricopeptide repeat
Ca-CNMS341	28367038	CaChr5	19078305	(AG)7	TGATAGCGGGAAACACATCT	GCGCTTCAATGTGGTAGGTT	58.2	279	CTRMCAMV35S	TCTCTCTCT	CaMV 35S (Inverted GAGA) found in a 60-nucleotide region downstream of the transcription start site of the	WD40 repeat
Ca-CNMS342	28367039	CaChr5	19349089	(AGC)5	TTCATTTGTACAATGGGCGA	AAAAAGGCAGCAGACCTCAA	59.9	238	ANAERO2CONSENSUS	AGCAGC	Motif found in silico in promoters of 13 anaerobic genes involved in the fermentative pathway	Peptidase C48, SUMO/Sentrin/Ubl1

Marker IDs	NCBI Probe IDs (PUIDs)	Chromosomes	Physical positions (bp)	Microsatellite repeat-motifs	Forward Primer (5'-3')	Reverse Primer (5'-3')	Actual annealing tempearture (0C)	Size (bp) of alleles amplified	Regulatory elements	Signal sequences of known regulatory element-binding sites	Function of known regulatory elements/ transcription factor-binding sites	Putative gene function
Ca-CNMS343	28367040	CaChr5	21840176	(TC)11	TGCCCCTTTGGAGATATCATT	TCATATTGGTTTGATATTGGATTG	60.6	139	CTRMCAMV35S	TCTCTCTCT	CaMV 35S (Inverted GAGA) found in a 60-nucleotide region downstream of the transcription start site of the	FBD-like
Ca-CNMS344	28367041	CaChr5	22631372	(CAA)5	TGGAGAATTGGCTGGAGTTC	TGGATTTTAAAGGGCTCCAC	60.2	242	RAV1AAT	CAACA	AP2 & B3 like domiane in RAV1	Bromodomain
Ca-CNMS345	28367042	CaChr5	23873650	(CT)6	ACTTCCCTGTCGACACAAGC	TCTTTTGTTAATCAAGTTGGTTGC	60.3	212	CTRMCAMV35S	TCTCTCTCT	CaMV 35S (Inverted GAGA) found in a 60-nucleotide region downstream of the transcription start site of the	BTB/POZ-like
Ca-CNMS346	28367043	CaChr5	23930003	(TC)11	CCCTCCTTCCTTCACAATCA	GCATATCGAAAGGTGGTGGT	60.0	217	CTRMCAMV35S	TCTCTCTCT	CaMV 35S (Inverted GAGA) found in a 60-nucleotide region downstream of the transcription start site of the	WD40 repeat
Ca-CNMS347	28367044	CaChr5	24850024	(AGTT)5	ATGGACATTAGTTCCACCGC	GTCATTCTTTGCTTCTCCGC	59.8	199	MYB1LEPR	GTTAGTT	Tomato Pti4(ERF) regulates defence-related gene expression via GCC box and non-GCC box cis elements	Major facilitator superfamily MFS-1
Ca-CNMS348	28367045	CaChr5	26021517	(TC)6	AACTTGTGCATTTGGGAACC	GCAATGCTCAGCAAGTTTCA	59.8	120	CTRMCAMV35S	TCTCTCTCT	CaMV 35S (Inverted GAGA) found in a 60-nucleotide region downstream of the transcription start site of the	Tetratricopeptide TPR-1
Ca-CNMS349	28367046	CaChr5	26207339	(TC)12	TTCGCTACCTCATCCAAACC	GATCGACGATTTCACGTCCT	60.1	168	CTRMCAMV35S	TCTCTCTCT	CaMV 35S (Inverted GAGA) found in a 60-nucleotide region downstream of the transcription start site of the	Lateral organ boundaries, LOB
Ca-CNMS350	28367048	CaChr5	26426762	(TGA)4	AAGAGAGAAGTGGGAGAGAGA	TCCTTCTCTGATATCCTTAATTC	55.0	164	GTGANTG10	GTGA	Motif found in the promoter of the tobacco late pollen gene g10 which shows homology to pectate lyase	Plant WRKY transcription factor
Ca-CNMS351	28367049	CaChr5	26690734	(AG)6	AAACTGAGTGTTTGGTGTCGT	TCACCCTCATTACTTTTCCCA	57.2	200	CTRMCAMV35S	TCTCTCTCT	CaMV 35S (Inverted GAGA) found in a 60-nucleotide region downstream of the transcription start site of the	Disease resistance protein
Ca-CNMS352	28367050	CaChr5	27283973	(ATGGT)3	TCCCATCAAGATGCTAAAATA	AATCAATCTTTGAGTTGTTGC	55.0	149	S1FBOXSORPS1L21	ATGGTA	conserved both in spinach (S.o.) RPS1 and RPL21 genes encoding the plastid ribosomal protein S1 and L21	SHY2/IAA3 regulates multiple auxin responses in roots
Ca-CNMS353	28367051	CaChr5	27537498	(AG)7	TAGGGGGAAAAAGGAACTGG	GCTCGAAAACTCCATCAAGG	60.3	254	CTRMCAMV35S	TCTCTCTCT	CaMV 35S (Inverted GAGA) found in a 60-nucleotide region downstream of the transcription start site of the	DNA primase, small subunit
Ca-CNMS354	28367052	CaChr5	27692497	(TC)23	ACATGGTGGGGTCAGGTAAA	TGTACAACAAAGCTGCCCAA	60.1	206	CTRMCAMV35S	TCTCTCTCT	CaMV 35S (Inverted GAGA) found in a 60-nucleotide region downstream of the transcription start site of the	NA
Ca-CNMS355	28367053	CaChr5	28111742	(TC)6	CCCACCGAAAAATAAGTGGA	GGGATTTGTTTGTCGCTTGT	59.8	181	CTRMCAMV35S	TCTCTCTCT	CaMV 35S (Inverted GAGA) found in a 60-nucleotide region downstream of the transcription start site of the	Isopenicillin N synthase
Ca-CNMS356	28367054	CaChr5	29398032	(TTTTA)3	GGATCGAAAGGATTGTCTATT	TTGTGTGTTGTTGTAAAAATCC	55.0	164	TATABOX5	TTATTT	TATA box found in the 5'upstream region of pea glutamine synthetase gene	DREB subfamily A-6 of ERF/AP2 transcription factor family (RAP2.4)
Ca-CNMS357	28367055	CaChr5	29731848	(TCT)7	TAATGCTGGTGGTTGCATTG	CACATGCAGTGGGAAAAAGA	60.5	124	CTRMCAMV35S	TCTCTCTCT	CaMV 35S (Inverted GAGA) found in a 60-nucleotide region downstream of the transcription start site of the	Phosphate-induced protein 1
Ca-CNMS358	28367056	CaChr5	29786441	(TTC)5	TCATCGGAAGAAGAAGAAGAT	TTCTTTCCTTTCTTTCCTTTC	56.0	169	CTRMCAMV35S	TCTCTCTCT	CaMV 35S (Inverted GAGA) found in a 60-nucleotide region downstream of the transcription start site of the	AAA-type ATPase family protein
Ca-CNMS359	28367057	CaChr5	29879662	(TC)8	GTCCGAAAAGAAGGATGCAG	GAGAATGAGGAAGCAAACGC	59.8	185	CTRMCAMV35S	TCTCTCTCT	CaMV 35S (Inverted GAGA) found in a 60-nucleotide region downstream of the transcription start site of the	Bromodomain
Ca-CNMS360	28367059	CaChr5	30145632	(GGA)8	CGGCAAAGCAAACCTAAAAG	AATGGGCCTGGGAATTATCT	59.9	136	UPRMOTIFIIAT	CCNNNNNNNNNNNNCCACG	conserved UPR (unfolded protein response) cis-acting element in Arabidopsis	Alanyl-tRNA synthetase, class IIc
Ca-CNMS361	28367060	CaChr5	30198703	(GA)7	TGCATCCATTGTTGAAGCAT	ATCTTGTTGATGTCTCCGGC	60.1	265	GAGA8HVBKN3	GAGAGAGAGAGAGAGA	Motif found in intron IV of the barley (H.v.) gene Bkn3	Ribosomal protein L3
Ca-CNMS362	28367061	CaChr5	30595088	(AAGA)3	GCTGATCAGTTGTGGTAGAAG	CGACTTGAATCTCTCCTGTAA	55.0	161	POLLEN1LELAT52	AGAAA	One of two co-dependent regulatory elements responsible for pollen specific activation of tomato lat52	ZInc knuckle (CCHC-type) family protein
Ca-CNMS363	28367062	CaChr5	30595088	(TTTC)3	CGACTTGAATCTCTCCTGTAA	GCTGATCAGTTGTGGTAGAAG	55.0	161	NODCON2GM	CTCTT	One of two putative nodulin consensus sequences	ZInc knuckle (CCHC-type) family protein
Ca-CNMS364	28367063	CaChr5	30595088	(TTTC)3	CGACTTGAATCTCTCCTGTAA	GCTGATCAGTTGTGGTAGAAG	55.0	161	DOFCOREZM	AAAG	Site required for binding of Dof proteins in maize	ZInc knuckle (CCHC-type) family protein
Ca-CNMS365	28367064	CaChr5	30615609	(GTT)4	GTTCCCAGTCTTGAGAAAGAG	CCACACTTCATCTTCATCTTC	56.0	154	RAV1AAT	CAACA	AP2 & B3 like domiane in RAV1	Multiprotein bridging factor 1, a transcriptional coactivator
Ca-CNMS366	28367065	CaChr5	30631008	(CT)8	GTGGGGAGACTCAAAGACGA	AACTGACACCACCAGCAACA	60.2	212	CTRMCAMV35S	TCTCTCTCT	CaMV 35S (Inverted GAGA) found in a 60-nucleotide region downstream of the transcription start site of the	Iron hydrogenase, small subunit-like
Ca-CNMS367	28367066	CaChr5	30706855	(TTTA)5	AAAAATTGGCGATCAAAATGA	TGCAGAAGATTTTGGAATGTTT	59.4	262	TATABOX5	TTATTT	TATA box found in the 5'upstream region of pea glutamine synthetase gene	Gamma thionin
Ca-CNMS368	28367067	CaChr5	30725332	(CT)8	GCACCTAACAAGCTTCACCC	TCAACCAATTATGGCTTCTGC	59.7	179	CTRMCAMV35S	TCTCTCTCT	CaMV 35S (Inverted GAGA) found in a 60-nucleotide region downstream of the transcription start site of the	Basic-leucine zipper (bZIP) transcription factor
Ca-CNMS369	28367068	CaChr5	30725865	(AG)11	GGAGAGTTTGTTTGTTGAAAA	CACTCGAAATAAATCACCAAC	55.0	144	GAGAGMGSA1	(GA)9	Motif found in the promoter of the heme and chlorophyll synthesis gene Gsa1 in soybean	MYC-related transcriptional activator
Ca-CNMS370	28367070	CaChr5	30725865	(AAAAG)3	AAATACATAACCAGCTCAACG	GGGGTGTAGTTTAAGGTTGTT	55.0	154	DOFCOREZM	AAAG	Site required for binding of Dof proteins in maize	VND-interacting 1 (VNI1)
Ca-CNMS371	28367071	CaChr5	30990358	(TTC)4	GATACGAGCAACTAACCACAC	CTCTTTGAAGAGGAAAAGTGG	55.0	166	CCAATBOX1	CAAT	Consequence seq. found in LegA gene	ARID/BRIGHT DNA-binding domain-containing protein
Ca-CNMS372	28367072	CaChr5	31022138	(CT)7	CCATCACCACCTTTTCACCT	AAACCCTAGAATTGGGGTGG	59.8	127	CTRMCAMV35S	TCTCTCTCT	CaMV 35S (Inverted GAGA) found in a 60-nucleotide region downstream of the transcription start site of the	RNA recognition motif domain
Ca-CNMS373	28367073	CaChr5	31022138	(TC)7	CGTCCGATCTTTCTCTCCAC	AGGTGAAAAGGTGGTGATGG	59.8	190	CTRMCAMV35S	TCTCTCTCT	CaMV 35S (Inverted GAGA) found in a 60-nucleotide region downstream of the transcription start site of the	RNA recognition motif domain
Ca-CNMS374	28367074	CaChr5	31512049	(TTTA)6	ACGGGATTTTCCGTTCAAA	TTTCCAACCCACAAAATCAA	60.3	259	TATABOX5	TTATTT	TATA box found in the 5'upstream region of pea glutamine synthetase gene	MaoC-like dehydratase
Ca-CNMS375	28367075	CaChr5	32661491	(AATT)5	CGAACACGGACAGAAAGTCA	TGGTGGATTCATGGAGATCA	59.9	273	GCCCORE	GCCGCC	Motif found in many pathogen-responsive genes such as PDF1.2, Thi2.1 and PR4	Protein kinase, catalytic domain
Ca-CNMS376	28367076	CaChr5	33142260	(AG)6	ACGCTCCTTGTTGTTGTGTT	TGCTTTCATTCGTTGGTTCA	58.3	173	CTRMCAMV35S	TCTCTCTCT	CaMV 35S (Inverted GAGA) found in a 60-nucleotide region downstream of the transcription start site of the	Protein kinase, catalytic domain
Ca-CNMS377	28367077	CaChr5	33528738	(GTT)7	TCATGTTTCTGACCAGGCAA	TCTCGTCGTCATCATCCAAA	60.2	121	CAREOSREP1	CAACTC	Motif found in REP-1 gene promoter	Exostosin-like
Ca-CNMS378	28367078	CaChr5	34003075	(TTTA)5	CTCCATCATGCTCCGATTTT	TTGGAAGAAAGAGTGAGGAAGC	60.0	142	TATABOX5	TTATTT	TATA box found in the 5'upstream region of pea glutamine synthetase gene	UAA transporter
Ca-CNMS379	28367079	CaChr5	34474832	(TGT)4	TCTTAGTAACGCCATGGTAAA	GAGACATACATACGCCCATTA	55.0	153	CAREOSREP1	CAACTA	Motif found in REP-1 gene promoter	TIFY domain protein 8 (TIFY8)
Ca-CNMS380	28367081	CaChr5	34505910	(TC)8	CTTCACCCCTTGGATTTT	GTTGTTGTTGATGCTGTGAC	55.0	130	CTRMCAMV35S	TCTCTCTCT	CaMV 35S (Inverted GAGA) found in a 60-nucleotide region downstream of the transcription start site of the	LEUNIG
Ca-CNMS381	28367082	CaChr5	34608921	(TC)10	TCTCTTCGATTTGCGGTTCT	CCAATGGACGAACAAATCAA	60.0	105	CTRMCAMV35S	TCTCTCTCT	CaMV 35S (Inverted GAGA) found in a 60-nucleotide region downstream of the transcription start site of the	Agenet
Ca-CNMS382	28367083	CaChr5	34781930	(CT)7	TTGGATTTCTGTCGCAAGGT	GGTCACTGCATGCAAATTAAA	60.6	270	CTRMCAMV35S	TCTCTCTCT	CaMV 35S (Inverted GAGA) found in a 60-nucleotide region downstream of the transcription start site of the	C2 calcium-dependent membrane targeting
Ca-CNMS383	28367084	CaChr5	34805574	(TC)13	CCACCCCTTTCTAAATTCTCAA	AGCTTCAAAAACCAGCGAAA	59.5	245	CTRMCAMV35S	TCTCTCTCT	CaMV 35S (Inverted GAGA) found in a 60-nucleotide region downstream of the transcription start site of the	F-box domain, cyclin-like
Ca-CNMS384	28367085	CaChr5	35494628	(GAAA)5	TGAGTCGAATATGGAGGGATG	AGAGGACGCGGTGGTAGG	59.9	221	POLLEN1LELAT52	AGAAA	One of two co-dependent regulatory elements responsible for pollen specific activation of tomato lat52	Pathogenesis-related transcriptional factor/ERF, DNA-
Ca-CNMS385	28367086	CaChr5	35505614	(AGAA)5	TGACTCAGCAGCCAAATGAC	CGGAGGAACATCGATACCTT	60.0	275	POLLEN1LELAT52	AGAAA	One of two co-dependent regulatory elements responsible for pollen specific activation of tomato lat52	Sterile alpha motif domain

Marker IDs	NCBI Probe IDs (PUIDs)	Chromosomes	Physical positions (bp)	Microsatellite repeat-motifs	Forward Primer (5'-3')	Reverse Primer (5'-3')	Actual annealing tempearture (0C)	Size (bp) of alleles amplified	Regulatory elements	Signal sequences of known regulatory element-binding sites	Function of known regulatory elements/ transcription factor-binding sites	Putative gene function
Ca-CNMS386	28367087	CaChr5	35528071	(TC)14	TCGTTTTTCACTCACTCCCC	CTGCGTGTTGTTCCATTTTG	60.1	250	CTRMCAMV35S	TCTCTCTCT	CaMV 35S (Inverted GAGA) found in a 60-nucleotide region downstream of the transcription start site of the	BRCT
Ca-CNMS387	28367088	CaChr5	36356814	(CAA)6	AACACCAGCAACAATCCCTC	TCGGTGCTTGATGGATGATA	60.0	237	RAV1AAT	CAACA	AP2 & B3 like domiane in RAV1	Ubiquitin system component Cue
Ca-CNMS388	28367089	CaChr5	36652765	(AGATGA)3	CGATGAATTCAAGAAGCAATA	TGAACCTAACCAAATTCTTGA	56.0	159	MYCCONSENSUSAT	CANNTG	Motif found in the promoters of the dehydration-responsive gene rd22	DREB subfamily A-5 of ERF/AP2 transcription factor family (RAP2.1)
Ca-CNMS389	28367090	CaChr5	36753273	(GTT)4	GGGATTCAGATTTGTGATTTA	CAGAGAATATACCGACGTTTG	54.0	195	RAV1AAT	CAACA	AP2 & B3 like domians in RAV1	Leucine zipper transcription factor
Ca-CNMS390	28367092	CaChr5	37349151	(TTTA)7	ATTAGCTGTTTCAAGGTTTGCT	AAATGCAACATCGTAGAAGATCAA	57.3	120	TATABOX5	TTATTT	TATA box found in the 5'upstream region of pea glutamine synthetase gene	KIP1-like
Ca-CNMS391	28367093	CaChr5	37943851	(CT)8	GTACACAAACAAATCCACGAT	AAGCAACGACAAGATACAAAA	55.0	144	CTRMCAMV35S	TCTCTCTCT	CaMV 35S (Inverted GAGA) found in a 60-nucleotide region downstream of the transcription start site of the	Squamosa promoter-binding protein-like (SBP domain)
Ca-CNMS392	28367094	CaChr5	38288356	(AAC)7	TGAACAACGTTTCTTCTCTTC	GTTTTGGAACTGAAATCAACA	55.0	148	RAV1AAT	CAACA	AP2 & B3 like domiane in RAV1	MYC-like bHLH transcriptional activator
Ca-CNMS393	28367095	CaChr5	38500047	(CT)7	AAAGCAAGTGCCGTAGTGCT	TGAAATTTGAAAGTGGTTGGTG	60.1	144	CTRMCAMV35S	TCTCTCTCT	CaMV 35S (Inverted GAGA) found in a 60-nucleotide region downstream of the transcription start site of the	Xanthine/uracil/vitamin C permease
Ca-CNMS394	28367096	CaChr5	39343430	(TC)6	TTTTTGAAAAGTGCTCACGA	GACAAAATGTGAGTGGAGACCA	57.1	212	CTRMCAMV35S	TCTCTCTCT	CaMV 35S (Inverted GAGA) found in a 60-nucleotide region downstream of the transcription start site of the	ATPase-like, ATP-binding domain
Ca-CNMS395	28367097	CaChr5	39841264	(CCG)5	CCAAGACCATTCAGCCAACT	TGGTGAAAAGAAGGACATGG	60.1	120	GCCCORE	GCCGCC	Core of GCC-box found in many pathogen-responsive genes such as PDF1.2, Thi2.1 and PR4	Protein kinase, catalytic domain
Ca-CNMS396	28367098	CaChr5	39880863	(TTTA)5	CATTAATCAACGATTTGGTCG	CATGCATGTGATACATTGACTTC	58.0	269	TATABOX5	TTATTT	TATA box found in the 5'upstream region of pea glutamine synthetase gene	Xanthine/uracil/vitamin C permease
Ca-CNMS397	28367099	CaChr5	40012531	(TATT)7	TGACGAACTTCAAGATGTACATAAAA	TTAGTGGTGACATTGGCGTT	59.2	198	TATABOX5	TTATTT	TATA box found in the 5'upstream region of pea glutamine synthetase gene	Lipase, class 3
Ca-CNMS398	28367100	CaChr5	40468062	(CT)6	AGACAGCAGTGCCTGCTACA	CACCCTCTCACTCACGCATA	59.8	232	CTRMCAMV35S	TCTCTCTCT	CaMV 35S (Inverted GAGA) found in a 60-nucleotide region downstream of the transcription start site of the	Double-stranded RNA-binding
Ca-CNMS399	28367101	CaChr5	40619206	(CT)6	AGAGAACCCAATACCAAAAAC	CTGACGCAGTAGCCTTCTT	55.0	174	CTRMCAMV35S	TCTCTCTCT	CaMV 35S (Inverted GAGA) found in a 60-nucleotide region downstream of the transcription start site of the	SWIB/MDM2 domain superfamily protein
Ca-CNMS400	28367104	CaChr5	41534854	(AGTGC)5	ATTTGTTTTTGGGCATTTGG	GCGAGCTGGTTTTGTTTAGTG	59.7	145	CACTFTPPCA1	YACT	Tetranucleotide (CACT) is a key component of Mem1 found in the cis-regulatory element in the distal region of	BTB/POZ-like
Ca-CNMS401	28367105	CaChr5	41898933	(TC)12	TAAATCGAGCACCCCAAATC	TGAAAGATGCGGAGTGAGTG	59.9	272	CTRMCAMV35S	TCTCTCTCT	CaMV 35S (Inverted GAGA) found in a 60-nucleotide region downstream of the transcription start site of the	Peptidase C19, ubiquitin carboxyl-terminal hydrolase 2
Ca-CNMS402	28367106	CaChr5	42499426	(AG)6	ATGGTTTTGCTCACATTCTTA	AACTCACACAGAACACTGCTT	55.0	151	CTRMCAMV35S	TCTCTCTCT	CaMV 35S (Inverted GAGA) found in a 60-nucleotide region downstream of the transcription start site of the	MADs box transcription factor family
Ca-CNMS403	28367107	CaChr5	42949608	(TC)8	TGATGTTGAGTATAAGCCCCC	GCTAATCTTCTTCGTCGTTTGG	58.9	183	CTRMCAMV35S	TCTCTCTCT	CaMV 35S (Inverted GAGA) found in a 60-nucleotide region downstream of the transcription start site of the	Protein phosphatase 2A, regulatory B subunit, B56
Ca-CNMS404	28367108	CaChr5	42980949	(AACAGA)3	TTTTTGAACTCAAACAGAAGC	ATCATCAACAACAAGAACTCG	55.0	142	RAV1AAT	CAACA	AP2 & B3 like domians in RAV1	Arabidopsis response regulator (ARR) protein
Ca-CNMS405	28367109	CaChr5	43832181	(AAAGAT)3	CCATGGATAAAGAAGAAATGA	CCAAACTCCTCTGATCCATA	54.0	145	DOFCOREZM	AAAG	Site required for binding of Dof proteins in maize	Homeobox-containing gene with an unusual feature
Ca-CNMS406	28367110	CaChr5	44153023	(ATCC)3	ACATCCATCACACATCACAC	AGAGAGAGAAATTGAGGGAAA	55.0	151	AMYBOX2	TATCCAT	Motif found in 5'upstream region of alpha-amylase gene of rice	SWI3 gene family protein
Ca-CNMS407	28367111	CaChr5	44394350	(TCT)4	AAACTTGAGCATCACAAAGAA	CACAAGAACACACAAAGAAAA	55.0	177	ARR1AT	NGATT	Motif found in the promoter of rice NSHB gene	Floral homeotic gene encoding a MADS domain transcription factor
Ca-CNMS408	28367112	CaChr5	44483437	(CT)7	CAACAGAGCAAAGCAGAGCA	TGTGTTTGTTCTGATGCAAGC	60.5	181	CTRMCAMV35S	TCTCTCTCT	CaMV 35S (Inverted GAGA) found in a 60-nucleotide region downstream of the transcription start site of the	BTB/POZ-like
Ca-CNMS409	28367113	CaChr5	44560031	(AG)8	ACTTACTGCAAAAGAGCAACA	AGAAAGACCTTGTCCTTCAAC	55.0	147	GAGA8HVBKN3	(GA)8	Motif found in intron IV of the barley (H.v.) gene Bkn3	S-adenosyl-L-methionine-dependent methyltransferases
Ca-CNMS410	28367115	CaChr5	45536435	(CGG)5	AATACCATTTTGCACGAGCC	GAATTGGACGAGGCTTTCAG	60.0	187	GCCCORE	GCCGCC	Motif found in many pathogen-responsive genes such as PDF1.2, Thi2.1 and PR4	Acyl-CoA-binding protein, ACBP
Ca-CNMS411	28367116	CaChr5	47007675	(AGC)4	CTGGTGTATTTTTCGTCAGTC	CAAGATTCAAAATAGGGGTCT	55.0	156	ANAERO1CONSENSUS	AAACAAA	Motif found in silico in promoters of 13 anaerobic genes involved in the fermentative pathway	Putative transposase
Ca-CNMS412	28367117	CaChr5	47098913	(TTATT)5	CCTCATGAGAAACTCGCCTC	TGAAAACGGATAATCGACAAAA	60.0	127	TATABOX5	TTATTT	TATA box found in the 5'upstream region of pea glutamine synthetase gene	Protein kinase, catalytic domain
Ca-CNMS413	28367118	CaChr5	47316749	(ATGCA)4	GAAAAAGACAGAGGAAAAAGAA	GCCTTAGCCAAAGACATTTAC	55.0	163	CAATBOX1	CAAT	CAAT promoter consensus sequence found in legA gene of pea	CONSTANS-like 4 (COL4)
Ca-CNMS414	28367119	CaChr5	48054974	(GA)6	AGTCCCTCAACGTTCAAAATG	TTTTTGGTGTGGTTCCATTG	59.1	246	GAGA8HVBKN3	GAGAGAGAGAGAGAGA	Motif found in intron IV of the barley (H.v.) gene Bkn3	Pentatricopeptide repeat
Ca-CNMS415	28367120	CaChr6	1129778	(CTG)5	TGTATCAAATTGGGTGGCAA	TCTTTGATTGCCTCTGGACC	59.8	263	ANAERO2CONSENSUS	AGCAGC	Motif found in silico in promoters of 13 anaerobic genes involved in the fermentative pathway	Domain of unknown function DUF296
Ca-CNMS416	28367121	CaChr6	1129778	(AGG)5	CAGACTTCGTCCTCCTCCTTT	TTGCCACCCAATTTGATACA	59.9	116	UPRMOTIFIIAT	CCNNNNNNNNNNNNCCAC G	conserved UPR (unfolded protein response) cis-acting element in Arabidopsis	Domain of unknown function DUF296
Ca-CNMS417	28367122	CaChr6	1609295	(TCATTT)3	CCTTCCTTTTCAACCTCTATT	TCTGGTTCATGAAAAATTCAC	54.0	153	INRNTPSADB	YTCANTYY	Found in tobacco Psb gene promoter without TATA box	DA1-related protein 2 (DAR2)
Ca-CNMS418	28367123	CaChr6	1609295	(TCATTT)3	CCTTCCTTTTCAACCTCTATT	CCATAAGGATGATGGAAACTT	54.0	153	INRNTPSADB	YTCANTYY	Motif found in the tobacco psaDb gene promoter without TATA boxes	DA1-related protein 2 (DAR2)
Ca-CNMS419	28367124	CaChr6	1609295	(TTCTCT)3	TACCTCCACATCATTTTCATT	TATATGCAAGGGTGAGAAAGA	55.0	134	NODCON2GM	CTCTT	One of two putative nodulin consensus sequences	DA1-related protein 2 (DAR2)
Ca-CNMS420	28367126	CaChr6	1609295	(TTCTCT)3	TACCTCCACATCATTTTCATT	CCATAAGGATGATGGAAACTT	55.0	134	NODCON2GM	CTCTT	Putative nodulin consensus sequences	DA1-related protein 2 (DAR2)
Ca-CNMS421	28367127	CaChr6	2062113	(TAG)7	TCAATGGAAATGCCATCAAA	TGTTGCTCCTTTCTTTGCCT	59.9	232	CACTFTPPCA1	YACT	Tetranucleotide (CACT) is a key component of Mem1 found in the cis-regulatory element in the distal region of	Poly(A) polymerase, RNA-binding domain
Ca-CNMS422	28367128	CaChr6	2204973	(AG)26	CATGAAATTCGGTGCATTTG	AACGCCCTAAACCTCTTGGT	59.9	179	GAGA8HVBKN3	GAGAGAGAGAGAGAGA	Motif found in intron IV of the barley (H.v.) gene Bkn3	RNA recognition motif domain
Ca-CNMS423	28367129	CaChr6	2232010	(TC)10	TCATGATCGTCACGTCGTTT	TGAAGTGAAGAGAGATGAGGTTG	60.1	145	CTRMCAMV35S	TCTCTCTCT	CaMV 35S (Inverted GAGA) found in a 60-nucleotide region downstream of the transcription start site of the	Homeobox
Ca-CNMS424	28367130	CaChr6	2232010	(GA)8	GCAAGATTCCAAATGGGGTA	TTCCCTCTTTACCCTTTTCTTTG	59.8	277	GAGA8HVBKN3	GAGAGAGAGAGAGAGA	Motif found in intron IV of the barley (H.v.) gene Bkn3	Homeobox
Ca-CNMS425	28367131	CaChr6	2932727	(TGC)5	CTGCCGCGTTCATGATAGTA	TCTTCTCATGCTTGCCTCAA	59.9	102	ANAERO2CONSENSUS	AGCAGC	Motif found in silico in promoters of 13 anaerobic genes involved in the fermentative pathway	F-box domain, cyclin-like
Ca-CNMS426	28367132	CaChr6	3288313	(CT)6	CCTCCCTCTTGCGTACAAGT	CATTTTGAGAGTTGCAGCGA	59.4	232	CTRMCAMV35S	TCTCTCTCT	CaMV 35S (Inverted GAGA) found in a 60-nucleotide region downstream of the transcription start site of the	Aldehyde dehydrogenase domain
Ca-CNMS427	28367133	CaChr6	3653440	(TAC)5	TTTTCTATTTTGGGGGTTGAAA	AGAGGGAATGTGGGGCTTAT	59.7	147	CACTFTPPCA1	YACT	Tetranucleotide (CACT) is a key component of Mem1 found in the cis-regulatory element in the distal region of	Pathogenesis-related transcriptional factor/ERF, DNA-
Ca-CNMS428	28367134	CaChr6	4214744	(GTG)4	TGCAGTAGAAGAAGCAGAGAG	AGACCTATCTTTTCCGTTTTG	55.0	153	CACTFTPPCA1	YACT	Tetranucleotide (CACT) is a key component of Mem1 found in the cis-regulatory element in the distal region of	NAC domain containing protein 50 (NAC050)

Marker IDs	NCBI Probe IDs (PUIDs)	Chromosomes	Physical positions (bp)	Microsatellite repeat-motifs	Forward Primer (5'-3')	Reverse Primer (5'-3')	Actual annealing tempearture (0C)	Size (bp) of alleles amplified	Regulatory elements	Signal sequences of known regulatory element-binding sites	Function of known regulatory elements/ transcription factor-binding sites	Putative gene function
Ca-CNMS429	28367135	CaChr6	4660464	(CAA)7	AGAACAACCACAAAGACCGC	TGGAATCCCTTTGATTGGTC	60.2	223	RAV1AAT	CAACA	AP2 & B3 like domiane in RAV1	Myb, DNA-binding
Ca-CNMS430	28367137	CaChr6	4670464	(CTCAAG)3	GAACCTCATAATTCATCCACA	GGTTATGTGGTTGTTGTTGTT	55.0	160	GATABOX	GATA	GATA motif in CaMV 35S promoter required for high level, light regulated, and tissue specific expression	KANADI protein (KAN)
Ca-CNMS431	28367138	CaChr6	5258897	(TGT)5	GGGTTTCTCGATCTTGCACT	TGATGGTTAACGAACCCGAT	59.3	120	CAREOSREP1	CAACTC	Motif found in REP-1 gene promoter	BTB/POZ-like
Ca-CNMS432	28367139	CaChr6	5504731	(TTG)4	AAAAACAAGGAACCAAAGAAG	ATCTCCTCTATCATTGGTGGT	55.0	126	RAV1AAT	CAACA	AP2 & B3 like domians in RAV1	Nuclear coiled-coil protein related to the carrot peripheral nuclear
Ca-CNMS433	28367140	CaChr6	5504731	(TGT)5	AATCTTTCTTTCTTTCCCAAG	GTTGTTCCTTCATGTTTTCAG	54.0	151	RAV1AAT	CAACA	AP2 & B3 like domians in RAV1	basic helix loop helix domain protein
Ca-CNMS434	28367141	CaChr6	6028228	(AG)8	ATCTCTGGCTGAAAGAAAAAT	GACAAAGAAAGCTGAGTGAAA	55.0	136	CTRMCAMV35S	TCTCTCTCT	CaMV 35S (Inverted GAGA) found in a 60-nucleotide region downstream of the transcription start site of the	Basic pentacysteine 4 (BPC4)
Ca-CNMS435	28367142	CaChr6	6028228	(GA)12	GAAAACCCAAGAGAGAGAGAG	GCTGTTGTTGTGAGTTTCTTC	55.0	138	GAGA8HVBKN3	(GA)8	Motif found in intron IV of the barley (H.v.) gene Bkn3	Basic pentacysteine 4 (BPC4)
Ca-CNMS436	28367143	CaChr6	6145054	(AAG)5	AGTATCTGGCTCCGCCTACA	ATGCCTGTTTACGATCTCCG	59.9	256	CTRMCAMV35S	TCTCTCTCT	CaMV 35S (Inverted GAGA) found in a 60-nucleotide region downstream of the transcription start site of the	AUX/IAA protein
Ca-CNMS437	28367144	CaChr6	6991977	(CT)21	CACAATCTTCCTCGCTCTCC	CAACCACAATTTTAGGTCATCAGA	60.0	187	CTRMCAMV35S	TCTCTCTCT	CaMV 35S (Inverted GAGA) found in a 60-nucleotide region downstream of the transcription start site of the	Histone deacetylase superfamily
Ca-CNMS438	28367145	CaChr6	7917140	(TAC)5	TCCACATTCTACCAACTCATTCA	AAGTTTCTGTCTTGGCTGCAA	59.5	269	CACTFTPPCA1	YACT	Tetranucleotide (CACT) is a key component of Mem1 found in the cis-regulatory element in the distal region of	Ethylene insensitive 3
Ca-CNMS439	28367146	CaChr6	7956943	(AAGA)5	TTCTTCATTGAATGGGTCTAA	GAATTTTGGATTGAAACAGGT	55.0	140	POLLEN1LELAT52	AGAAA	One of two co-dependent regulatory elements responsible for pollen specific activation of tomato lat52	DREB subfamily A-6 of ERF/AP2 transcription factor family
Ca-CNMS440	28367148	CaChr6	8141254	(CAA)5	GCATAAAGTTCAATGAGAACACTTACA	GCAATATTGTTACTGGGCGAA	60.0	142	RAV1AAT	CAACA	AP2 & B3 like domiane in RAV1	ATPase, P-type, H+ transporting proton pump
Ca-CNMS441	28367149	CaChr6	8366515	(AAAAC)5	CGTTGCTTAAGATACCAAAAC	AGAGCTTCCAAGTCTCTTCAT	54.0	179	ANAERO3CONSENSUS	TCATCAC	Motif found in silico in promoters of 13 anaerobic genes involved in the fermentative pathway	homeodomain leucine zipper class I (HD-Zip I) protein
Ca-CNMS442	28367150	CaChr6	8892200	(TC)6	GTTGTGGGGATCAGAACTCG	AGTTTTAGTGATGCCTCGCC	60.5	255	CTRMCAMV35S	TCTCTCTCT	CaMV 35S (Inverted GAGA) found in a 60-nucleotide region downstream of the transcription start site of the	Peptidase S8/S53, subtilisin/kexin/sedolisin
Ca-CNMS443	28367151	CaChr6	9118132	(AG)10	CCACAGCTTCAACGACAAGA	CAATGGTAAAAGGGGCTTGA	60.0	128	GAGA8HVBKN3	GAGAGAGAGAGAGAGA	Motif found in intron IV of the barley (H.v.) gene Bkn3	Ubiquitin
Ca-CNMS444	28367152	CaChr6	9119132	(AG)10	AGGTAGTTGAAGAAACGAACC	AGAAGCAGGATCAATACCTTT	55.0	153	GAGA8HVBKN3	(GA)8	Motif found in intron IV of the barley (H.v.) gene Bkn3	Basic-leucine zipper (bZIP) transcription factor family protein
Ca-CNMS445	28367153	CaChr6	9176524	(TTTA)5	CTTGGAACACGCATTCCTCT	TACTCGGTCACATGAGTCGC	60.3	279	TATABOX5	TTATTT	TATA box found in the 5'upstream region of pea glutamine synthetase gene	Protein kinase, catalytic domain
Ca-CNMS446	28367154	CaChr6	9935518	(AG)6	CGGGTGTTGATGAGAGAGAA	AATGAAGAATTTGAGCGCGT	58.8	205	CTRMCAMV35S	TCTCTCTCT	CaMV 35S (Inverted GAGA) found in a 60-nucleotide region downstream of the transcription start site of the	Protein kinase, catalytic domain
Ca-CNMS447	28367155	CaChr6	10028808	(TAC)4	AAATATCCGGAAGTATCTCCA	GGCTTTGTTATGTTTGAGGTT	55.0	134	CACTFTPPCA1	YACT	Tetranucleotide (CACT) is a key component of Mem1 found in the cis-regulatory element in the distal region of	Mitochondrial substrate carrier family protein
Ca-CNMS448	28367156	CaChr6	10769455	(AG)7	GCCCATATAAGCGCAACACT	GCTTGGATGAAATTGCAGGT	60.1	270	CTRMCAMV35S	TCTCTCTCT	CaMV 35S (Inverted GAGA) found in a 60-nucleotide region downstream of the transcription start site of the	Pentatricopeptide repeat
Ca-CNMS449	28367157	CaChr6	10864379	(CT)8	CTTTCTCTGATCCTTTTCGAT	GATGCCATGATATTGAATCTG	55.0	154	CTRMCAMV35S	TCTCTCTCT	CaMV 35S (Inverted GAGA) found in a 60-nucleotide region downstream of the transcription start site of the	Calmodulin-binding transcription activator protein
Ca-CNMS450	28367159	CaChr6	11012090	(GA)15	AACAATTAAACAAAAGGGGGTG	AGGATCTCCCTCAACATCTCC	59.2	116	GAGA8HVBKN3	GAGAGAGAGAGAGAGA	Motif found in intron IV of the barley (H.v.) gene Bkn3	Rubber elongation factor
Ca-CNMS451	28367160	CaChr6	11120286	(CAA)5	AGATGGGGACGATAGGTTCC	TTGGTTTATGTGAATGAATCGG	60.2	100	RAV1AAT	CAACA	AP2 & B3 like domiane in RAV1	Glycoside hydrolase, family 63
Ca-CNMS452	28367161	CaChr6	11146543	(TTG)5	TTTTCGCGCTTCTTTGAGTT	CCATTGGAGGCAGAAAATTG	60.1	266	CAREOSREP1	CAACTC	Motif found in REP-1 gene promoter	Armadillo
Ca-CNMS453	28367162	CaChr6	12511998	(AAC)5	TGATGCATGCCAAAACTTGT	GGTGGCTGTTTCCATGAGTC	60.1	273	RAV1AAT	CAACA	AP2 & B3 like domiane in RAV1	Helicase, C-terminal
Ca-CNMS454	28367163	CaChr6	12571472	(TTC)4	AGCTAACAAAGAAAAACACGA	GAAAAACAAACTCCCTCCTTA	55.0	148	RAV1AAT	CAACA	AP2 & B3 like domiane in RAV1	GATA transcription factor gene GNC
Ca-CNMS455	28367164	CaChr6	13242952	(TTGGG)3	AGATTTGGATCCATCTTTCAT	TGCTGAGTCTGAATCTGAAGT	55.0	149	SEF3MOTIFGM	AACCCA	Motif found in the 5' upstream region of beta-conglycinin (7S globulin) gene	CCCH-type zinc finger family protein
Ca-CNMS456	28367165	CaChr6	13727390	(TC)15	GGCCCCATATCCACACCT	TGTCGTTTTGCTCAGCTTGT	60.6	141	CTRMCAMV35S	TCTCTCTCT	CaMV 35S (Inverted GAGA) found in a 60-nucleotide region downstream of the transcription start site of the	K+ potassium transporter
Ca-CNMS457	28367166	CaChr6	14332654	(CAG)5	GAAAAAGTGGGCTTCAACCA	AAACGGTGAAAGGAGTGTGC	60.1	153	ANAERO2CONSENSUS	AGCAGC	Motif found in silico in promoters of 13 anaerobic genes involved in the fermentative pathway	Protein kinase, catalytic domain
Ca-CNMS458	28367167	CaChr6	14676684	(TC)9	CAAAGTGCTTCGTGGTCTGA	CCCAGGCTATGGAATGTCAC	60.0	242	CTRMCAMV35S	TCTCTCTCT	CaMV 35S (Inverted GAGA) found in a 60-nucleotide region downstream of the transcription start site of the	ATPase, AAA+ type, core
Ca-CNMS459	28367168	CaChr6	14811443	(GGC)5	ATACGTGGGAGAGAAAAAGAT	AAGCCCATTTCATTTACACTT	55.0	162	GCCCORE	GCCGCC	Core of GCC-box found in many pathogen-responsive genes such as PDF1.2, Thi2.1 and PR4	Zinc finger (CCCH-type/C3HC4-type RING finger) family protein
Ca-CNMS460	28367170	CaChr6	14990535	(ATTCA)5	TTCCCCCATTCATTCTTCTG	TTGAACGCTGGTTTCTGATG	59.9	139	CAATBOX1	CAAT	CAAT promoter consensus sequence found in legA gene of pea	BTB/POZ-like
Ca-CNMS461	28367171	CaChr6	15394537	(CAAA)5	TGCATCGGTGACCTGTGTAT	ACGGAAGTTGTGTTTAGCGG	60.0	202	ANAERO2CONSENSUS	AGCAGC	Motif found in silico in promoters of 13 anaerobic genes involved in the fermentative pathway	Aminotransferase, class IV
Ca-CNMS462	28367172	CaChr6	16539013	(ACAA)6	CAGGTTCCTTCCTCTGTTGC	CTTTTTGGCTGAAATGGTCC	59.8	235	ANAERO2CONSENSUS	AGCAGC	Motif found in silico in promoters of 13 anaerobic genes involved in the fermentative pathway	Pectinesterase, catalytic
Ca-CNMS463	28367173	CaChr6	17170634	(TC)8	GGAGGAGTTGGTTTGGGAGT	TTGCACCAATCTAAGTAGGAAAA	60.3	280	CTRMCAMV35S	TCTCTCTCT	CaMV 35S (Inverted GAGA) found in a 60-nucleotide region downstream of the transcription start site of the	Heat shock protein Hsp20
Ca-CNMS464	28367174	CaChr6	17253912	(TTG)5	CCTTGCCTCTCACCATTGTT	CCCAAAGGAATCTCATAACACC	60.1	273	CAREOSREP1	CAACTC	Motif found in REP-1 gene promoter	Cytochrome P450
Ca-CNMS465	28367175	CaChr6	17366001	(AGAA)4	AAAGGAAAAATTAGGCAGAGA	AAGTGTCCCTGAGGAAAATAC	55.0	145	POLLENILELAT52	AGAAA	One of two co-dependent regulatory elements responsible for pollen specific activation of tomato lat52	ETTIN
Ca-CNMS466	28367176	CaChr6	17856344	(CAA)5	TGAAGTAGGTATGCCCTCGC	CACCTCCCGCTAATGTCAAT	60.2	188	RAV1AAT	CAACA	AP2 & B3 like domiane in RAV1	Helix-loop-helix DNA-binding domain
Ca-CNMS467	28367177	CaChr6	18714579	(TC)7	CCTTCACAATCTTCTCCCCA	TTTGTTAGAGACCGTGTTATCTTCC	60.0	112	CTRMCAMV35S	TCTCTCTCT	CaMV 35S (Inverted GAGA) found in a 60-nucleotide region downstream of the transcription start site of the	Protein of unknown function DUF579
Ca-CNMS468	28367178	CaChr6	19212632	(CT)8	ACCTTGTTGCTTTCGTGCTT	TTCAGGTCCACTGACAGGTT	59.9	160	CTRMCAMV35S	TCTCTCTCT	CaMV 35S (Inverted GAGA) found in a 60-nucleotide region downstream of the transcription start site of the	Glutathione S-transferase, C-terminal
Ca-CNMS469	28367179	CaChr6	19356155	(CT)7	GAGTACTCGCCACCCCAATA	GAGAAGAGAAGAAAAGGAGGGA	60.0	103	CTRMCAMV35S	TCTCTCTCT	CaMV 35S (Inverted GAGA) found in a 60-nucleotide region downstream of the transcription start site of the	Glycoside hydrolase, family 17
Ca-CNMS470	28367181	CaChr6	19467359	(CTC)5	CCCACTACTCATCCCCACAC	GAAGAGGAAGGCTAGGGAGG	60.2	196	CTRMCAMV35S	TCTCTCTCT	CaMV 35S (Inverted GAGA) found in a 60-nucleotide region downstream of the transcription start site of the	D111/G-patch
Ca-CNMS471	28367182	CaChr6	19849334	(AG)8	CCTAATAATCTTGCCGCTGG	ATCCGATGCTTGTTTGCTTC	59.7	183	CTRMCAMV35S	TCTCTCTCT	CaMV 35S (Inverted GAGA) found in a 60-nucleotide region downstream of the transcription start site of the	Zinc finger, DHHC-type, palmitoyltransferase

Marker IDs	NCBI Probe IDs (PUIDs)	Chromosomes	Physical positions (bp)	Microsatellite repeat-motifs	Forward Primer (5'-3')	Reverse Primer (5'-3')	Actual annealing tempearture (0C)	Size (bp) of alleles amplified	Regulatory elements	Signal sequences of known regulatory element-binding sites	Function of known regulatory elements/ transcription factor-binding sites	Putative gene function
Ca-CNMS472	28367183	CaChr6	20082878	(GTTA)5	AAAAGGGGGAAAAGCTCAAA	AATTCTCACTAGATATGGCCCC	60.0	192	MYB1LEPR	GTTAGTT	Tomato Pti4(ERF) regulates defence-related gene expression via GCC box and non-GCC box cis elements	Plastid lipid-associated protein/fibrillin conserved domain
Ca-CNMS473	28367184	CaChr6	20513313	(TTGATA)3	GTGGAAGTGAATTCAAGTGAG	GTCCAACACCATAACAATCAT	55.0	148	GATABOX	GATA	GATA motif in CaMV 35S promoter required for high level, light regulate and tissue specific expression	SET domain protein 35 (SDG35)
Ca-CNMS474	28367185	CaChr6	20694118	(AG)7	GGGCGCTTGTAGAGAGAAAC	TCGCAGAATGGGGTTTAGTT	59.1	137	CTRMCAMV35S	TCTCTCTCT	CaMV 35S (Inverted GAGA) found in a 60-nucleotide region downstream of the transcription start site of the	General substrate transporter
Ca-CNMS475	28367186	CaChr6	20762964	(AGAGA)3	AGGAGGGGTTTTCTCTTTATT	GAAACTGTTTTGTCGTTGAAG	55.0	166	CTRMCAMV35S	TCTCTCTCT	CaMV 35S (Inverted GAGA) found in a 60-nucleotide region downstream of the transcription start site of the	IAA-leucine resistant3 (ILR3)
Ca-CNMS476	28367187	CaChr6	20762964	(GA)16	AGTCAATCCGTAAGAAGAAGC	CAAAGAAAGATTTCAAAGTCG	55.0	145	GAGA8HVBKN3	(GA)8	Motif found in intron IV of the barley (H.v.) gene Bkn3	Calmodulin-binding transcription activator protein
Ca-CNMS477	28367188	CaChr6	20762964	(TGA)6	TTTTGTTTTCTGGGAAATTG	AATCCTATTCTCCCCTTCTCT	55.0	149	TATABOX5	TTATTT	TATA box found in the 5'upstream region of pea glutamine synthetase gene	IAA-leucine resistant3 (ILR3)
Ca-CNMS478	28367189	CaChr6	21403710	(ACT)6	AAGGATGATTGAAAATTGGGG	CGGAAGCACCAAAGAAAAAG	60.0	152	CACTFTPPCA1	YACT	Tetranucleotide (CACT) is a key component of Mem1 found in the cis-regulatory element in the distal region of	Methyltransferase type 11
Ca-CNMS479	28367190	CaChr6	21808510	(TTG)5	TGAAGGTGACAAAGGTGGTG	ATACACCCCGTCAACTCAGG	59.6	200	CAREOSREP1	CAACTC	Motif found in REP-1 gene promoter	Zinc finger, CCCH-type
Ca-CNMS480	28367192	CaChr6	22249674	(TTCACA)5	CCCACTTTCTCACTCACTCTT	ATGGATGATGGAGAGAGAGAG	56.0	163	CACTFTPPCA1	YACT	Tetranucleotide (CACT) is a key component of Mem1 found in the cis-regulatory element in the distal region of	GRAS family transcription factor
Ca-CNMS481	28367193	CaChr6	22249674	(TCACAC)3	ACATTCACATTCACATTCACA	TTCTGGAGGAGATAGTAGTGGT	55.0	165	CACTFTPPCA1	YACT	Tetranucleotide (CACT) is a key component of Mem1 found in the cis-regulatory element in the distal region of	GRAS family transcription factor
Ca-CNMS482	28367194	CaChr6	22445899	(AGC)5	TTCATTTGTACAATGGGCGA	AATCTGGATCGGGGAAAATC	59.9	143	ANAERO2CONSENSUS	AGCAGC	Motif found in silico in promoters of 13 anaerobic genes involved in the fermentative pathway	Peptidase C48, SUMO/Sentrin/Ubl1
Ca-CNMS483	28367195	CaChr6	22708526	(TC)20	CATTGTTAGGGCAAAACCGT	CGGAATCGAAGGAGTTTGTT	59.9	256	CTRMCAMV35S	TCTCTCTCT	CaMV 35S (Inverted GAGA) found in a 60-nucleotide region downstream of the transcription start site of the	Transcriptional factor B3
Ca-CNMS484	28367196	CaChr6	22913052	(CAA)5	AACAAAACGAGATTTTACCCGA	TCTTGTTGTTTTCCCCATTTTC	59.9	230	RAV1AAT	CAACA	AP2 & B3 like domiane in RAV1	ABC transporter-like
Ca-CNMS485	28367197	CaChr6	22944608	(TAC)5	GACCCTGCAAAGGTCCAGTA	GTTGTTGTCTTTGGTGGCAA	60.1	193	CACTFTPPCA1	YACT	Tetranucleotide (CACT) is a key component of Mem1 found in the cis-regulatory element in the distal region of	Aminotransferase-like, plant mobile domain
Ca-CNMS486	28367198	CaChr6	23893314	(GA)14	GGACATGGTGCTTCGAAAAT	GGTGACGTTGAAGTGCAAAA	59.9	238	GAGA8HVBKN3	GAGAGAGAGAGAGAGA	Motif found in intron IV of the barley (H.v.) gene Bkn3	WW/Rsp5/WWP
Ca-CNMS487	28367199	CaChr6	24447651	(TC)6	ACCAACTCACATCTAGAAGCA	TGTTGTTGATGAAGGAATTGT	55.0	157	CTRMCAMV35S	TCTCTCTCT	CaMV 35S (Inverted GAGA) found in a 60-nucleotide region downstream of the transcription start site of the	R2R3 factor gene family
Ca-CNMS488	28367200	CaChr6	26352012	(GAA)6	TGAAGGGTAGAAAGAATCACA	TTCCACATTAAGCAACACTTT	55.0	150	GTGANTG10	GTGA	GTGA Motif in tobacco late pollen gene	GRAS family transcription factor
Ca-CNMS489	28367201	CaChr6	26356162	(AGAA)5	GTGATCTGTCATCGTTTCAAT	AGGAAGCTTTGAGAAAGAGAA	55.0	141	POLLEN1LELAT52	AGAAA	One of two co-dependent regulatory elements responsible for pollen specific activation of tomato lat52	GRAS family Protein, transcription factor
Ca-CNMS490	28367203	CaChr6	26356162	(ATTTT)3	CCTCTTTTCTTTCTGCTTCTT	TAAGGTAAAACCGTTGGTGTA	55.0	151	TATABOX5	TTATTT	TATA box found in the 5'upstream region of pea glutamine synthetase gene	GRAS family Protein, transcription factor
Ca-CNMS491	28367204	CaChr6	26357390	(CTA)7	CGAATCACAAAATCCATCCC	TGCAAACCACTTTTCCTTCTG	60.1	140	CACTFTPPCA1	YACT	Tetranucleotide (CACT) is a key component of Mem1 found in the cis-regulatory element in the distal region of	Transcription factor GRAS
Ca-CNMS492	28367205	CaChr6	26764031	(AG)11	CCATAGGATGAAATGCTGGC	TGCATTAAACTTAAAATCCACACTC	60.4	124	GAGA8HVBKN3	GAGAGAGAGAGAGAGA	Motif found in intron IV of the barley (H.v.) gene Bkn3	Ras GTPase
Ca-CNMS493	28367206	CaChr6	27308231	(TC)14	CCATTGCACCATGAGAGAGA	CCGGAGAGAGTGAAAGCATC	59.8	150	CTRMCAMV35S	TCTCTCTCT	CaMV 35S (Inverted GAGA) found in a 60-nucleotide region downstream of the transcription start site of the	Pathogenesis-related transcriptional factor/ERF, DNA-
Ca-CNMS494	28367207	CaChr6	27826122	(CAA)5	CGCCAATTGTTCAAGGAACT	TGAAATTAGCGATTTGCGTG	60.1	181	RAV1AAT	CAACA	AP2 & B3 like domiane in RAV1	Transcription factor CBF/NF-Y/archaeal histone
Ca-CNMS495	28367208	CaChr6	29746005	(AG)9	GGAAGAATGGCATAAAAATG	CTCTCGAGGTAGGTGTGTTTA	55.0	154	GAGA8HVBKN3	(GA)8	Motif found in intron IV of the barley (H.v.) gene Bkn3	A basic helix-loop-helix encoding gene (BIGPETAL, BPE)
Ca-CNMS496	28367209	CaChr6	32087074	(GA)7	GCTTGATGATGCTTCACTGC	CCATGTGATGGAAATCAGTAGC	59.6	169	CTRMCAMV35S	TCTCTCTCT	CaMV 35S (Inverted GAGA) found in a 60-nucleotide region downstream of the transcription start site of the	YABBY protein
Ca-CNMS497	28367210	CaChr6	32225272	(ACA)5	GCCATTCTCATCAGCTCTGG	TCACTGTGGCACCATGATTT	60.9	255	RAV1AAT	CAACA	AP2 & B3 like domiane in RAV1	Tetratricopeptide TPR-1
Ca-CNMS498	28367211	CaChr6	34363617	(AAC)7	ACATACGTTTCTGGGCTTGC	TCAGCATCAATCATCTTTGTCA	60.1	201	RAV1AAT	CAACA	AP2 & B3 like domiane in RAV1	Alpha/beta hydrolase fold-1
Ca-CNMS499	28367212	CaChr6	37114063	(AG)6	CAACCATTGATGCTTTGACA	TCATGAGTGTGCAATATCATTTTC	58.1	280	CTRMCAMV35S	TCTCTCTCT	CaMV 35S (Inverted GAGA) found in a 60-nucleotide region downstream of the transcription start site of the	Peptidase C48, SUMO/Sentrin/Ubl1
Ca-CNMS500	28367215	CaChr6	38814251	(AG)13	AAACTGCAACTCTGCCAACC	GATGCGTGCAGCGTATAAAA	60.3	172	GAGA8HVBKN3	GAGAGAGAGAGAGAGA	Motif found in intron IV of the barley (H.v.) gene Bkn3	Transcription factor GRAS
Ca-CNMS501	28367216	CaChr6	38814251	(AG)12	GAGAGAGAGGAGAGCGAGCA	GCCATGAATGAACGAATGTG	60.1	171	GAGA8HVBKN3	GAGAGAGAGAGAGAGA	Motif found in intron IV of the barley (H.v.) gene Bkn3	Transcription factor GRAS
Ca-CNMS502	28367217	CaChr6	38897924	(AG)7	GGAATCCATTTTGACAAGCAA	GACATTGATGGTGTTGCAGG	59.9	179	GAGA8HVBKN3	GAGAGAGAGAGAGAGA	Motif found in intron IV of the barley (H.v.) gene Bkn3	Major sperm protein
Ca-CNMS503	28367218	CaChr6	41279319	(TTG)5	CACCACTGCTATTCATTGTTT	ATCCACTGTGACATCACTGAC	55.0	149	RAV1AAT	CAACA	AP2 & B3 like domians in RAV1	Growth regulating factor, transcription activator
Ca-CNMS504	28367219	CaChr6	41647513	(CAC)4	ACATTGAAAAGTCAGCGATAA	CTCTTTTCTTGCGTTATACCA	55.0	177	ARR1AT	NGATT	Found in the promoter of rice NSHB gene	Arabidopsis response regulator (ARR) protein
Ca-CNMS505	28367220	CaChr6	41935868	(TTG)5	TTCACAAAGACAAAAAGGTGT	GCAATCTTTTCCCATTGTACT	55.0	149	RAV1AAT	CAACA	AP2 & B3 like domians in RAV1	Auxin response factor 9 (ARF9)
Ca-CNMS506	28367221	CaChr6	45703332	(GA)18	GACATGGTTCGATTTGGAT	CACACTCACTCTCTTCTTCTTCT	56.0	153	GAGA8HVBKN3	(GA)8	Motif found in intron IV of the barley (H.v.) gene Bkn3	WRKY Transcription Factor
Ca-CNMS507	28367222	CaChr6	48690114	(AG)6	AACAATGGAGCAAAGCAACA	GGGAAATGGTGAAAGGGAAT	59.3	258	CTRMCAMV35S	TCTCTCTCT	CaMV 35S (Inverted GAGA) found in a 60-nucleotide region downstream of the transcription start site of the	Tetratricopeptide repeat
Ca-CNMS508	28367223	CaChr6	50854499	(TC)11	TGCTCCATTGTCACTTGCTC	CAGATCAAAGGTTGGATCAAAAA	60.0	172	CTRMCAMV35S	TCTCTCTCT	CaMV 35S (Inverted GAGA) found in a 60-nucleotide region downstream of the transcription start site of the	C2 calcium-dependent membrane targeting
Ca-CNMS509	28367224	CaChr6	52171271	(GATA)22	TGCAGTTAAATAAAAAGCATGAAA	TGTGTGTTGTATCCATTGCG	58.2	264	GATABOX	GATA	GATA motif in CaMV 35S promoter required for high level, light regulated, and tissue specific expression	ATPase, P-type, K/Mg/Cd/Cu/Zn/Na/Ca/Na/H-
Ca-CNMS510	28367226	CaChr6	52204594	(AAG)6	CGTTCTAGAATTACACCCTCA	ATCTGAGGCTTTTCTCACTTC	55.0	147	DOFCOREZM	AAAG	Site required for binding of Dof proteins in maize	Zinc knuckle (CCHC-type) family protein
Ca-CNMS511	28367227	CaChr6	52595261	(CT)14	TCGCGCTCTCTCTTTCTCTC	TAAGCGCGATGAAATGAATG	60.1	157	CTRMCAMV35S	TCTCTCTCT	CaMV 35S (Inverted GAGA) found in a 60-nucleotide region downstream of the transcription start site of the	Protein phosphatase 2C, manganese/magnesium aspartate
Ca-CNMS512	28367228	CaChr6	53165876	(CT)8	GAGCCACGACAAAAGTGTCA	GGTGGAGAAAGCAACGAATC	59.9	147	CTRMCAMV35S	TCTCTCTCT	CaMV 35S (Inverted GAGA) found in a 60-nucleotide region downstream of the transcription start site of the	Methyladenine glycosylase
Ca-CNMS513	28367229	CaChr6	53189233	(TC)7	GCCAATAATTGAAAAGCCCA	CTGATCGGAGATCGAGAACG	59.9	146	CTRMCAMV35S	TCTCTCTCT	CaMV 35S (Inverted GAGA) found in a 60-nucleotide region downstream of the transcription start site of the	ATPase, V1/A1 complex, subunit D
Ca-CNMS514	28367230	CaChr6	53437805	(GGA)5	GTGGGGGTGGTGACTTGTAG	TGCCTCCTCCTCCATATGTT	60.3	216	UPRMOTIFIIAT	CCNNNNNNNNNNNNCCACG	Conserved UPR (unfolded protein response) cis-acting element in Arabidopsis	Extensin repeat

Marker IDs	NCBI Probe IDs (PUIDs)	Chromosomes	Physical positions (bp)	Microsatellite repeat-motifs	Forward Primer (5'-3')	Reverse Primer (5'-3')	Actual annealing tempearture (0C)	Size (bp) of alleles amplified	Regulatory elements	Signal sequences of known regulatory element-binding sites	Function of known regulatory elements/ transcription factor-binding sites	Putative gene function
Ca-CNMS515	28367231	CaChr6	54267812	(TC)8	TCCCTTAAATTGACGCAACC	TGAAAGGTTAATGAAGCGGG	59.9	171	CTRMCAMV35S	TCTCTCTCT	CaMV 35S (Inverted GAGA) found in a 60-nucleotide region downstream of the transcription start site of the	Zinc finger, RING-type
Ca-CNMS516	28367232	CaChr6	55328730	(CT)9	GGCTATAACCGGTCACCATC	TGCAAAAACACACACACGAG	59.3	180	CTRMCAMV35S	TCTCTCTCT	CaMV 35S (Inverted GAGA) found in a 60-nucleotide region downstream of the transcription start site of the	Homeobox
Ca-CNMS517	28367233	CaChr6	55329730	(TAG)4	TCTCATCATCCTTATTTGGAA	CCAACACCTACTTGTTGAAGA	55.0	139	CACTFTPPCA1	YACT	Tetranucleotide (CACT) is a key component of Mem1 found in the cis-regulatory element in the distal region of	Homeodomain transcription factor
Ca-CNMS518	28367234	CaChr6	57452871	(AG)8	CATTAGGTTCAGCCTCCTGC	CTCGTGGTTCACTGTTTCCA	59.8	162	GAGA8HVBKN3	GAGAGAGAGAGAGAGA	Motif found in intron IV of the barley (H.v.) gene Bkn3	Protein of unknown function DUF2054
Ca-CNMS519	28367235	CaChr6	57660492	(ATTT)5	CTTGCTATATGGAGCTCGGG	TGAAAATCTCAACCCCTCAA	59.8	217	TATABOX5	TTATTT	TATA box found in the 5'upstream region of pea glutamine synthetase gene	Phox/Bem1p
Ca-CNMS520	28367237	CaChr6	57975183	(TTG)5	TAGGTTGGCAGTGAAATCCC	AACAGCACCATTTGTTGGAG	59.9	168	CAREOSREP1	CAACTC	Motif found in REP-1 gene promoter	Phospholipid/glycerol acyltransferase
Ca-CNMS521	28367238	CaChr6	58554985	(GTTT)3	TGCTTTTCTCTACTGGCTTTA	AGACACAAGATCATCCAAGTG	55.0	163	AACACOREOSGLUB1	AACAAAC	Motif found in rice (O.s.) glutelin genes, involved in controlling the endosperm-specific expression	Basic domain leucine zipper (bZip) transcription factor bZIP11
Ca-CNMS522	28367239	CaChr6	59406343	(TC)18	CTTCTTCTTCATTCGCCTCG	CGCCGCTCATAAACTCTCTAA	60.1	248	CTRMCAMV35S	TCTCTCTCT	CaMV 35S (Inverted GAGA) found in a 60-nucleotide region downstream of the transcription start site of the	Mitochondrial carrier protein
Ca-CNMS523	28367240	CaChr7	1101237	(AAACA)6	ATGGCCTTATCCTTCAACCC	TTTCCACATGCTTTGCTTTG	60.2	225	ANAERO1CONSENSUS	AAACAAA	Motif found in silico in promoters of 13 anaerobic genes involved in the fermentative pathway	IQ motif, EF-hand binding site
Ca-CNMS524	28367241	CaChr7	1185267	(ACA)5	GCAGCTACCTCAGCTTTTGG	TCTCAATCGCCAATTTTTGTT	60.2	156	RAV1AAT	CAACA	AP2 & B3 like domiane in RAV1	Ubiquitin/SUMO-activating enzyme E1
Ca-CNMS525	28367242	CaChr7	1270686	(TGT)4	ATCACCGTTAATCCAAATCTC	ACATCACCAAAACCTTAACAA	56.0	150	CAREOSREP1	CAACTC	Found in REP-1 gene promoter	SHI gene family protein
Ca-CNMS526	28367243	CaChr7	1447750	(TC)9	CCACCCCAAAGCCTAAAAAT	GGAAATCAAAGGATGCGTGT	60.2	278	CTRMCAMV35S	TCTCTCTCT	CaMV 35S (Inverted GAGA) found in a 60-nucleotide region downstream of the transcription start site of the	Zinc finger, U1-type
Ca-CNMS527	28367244	CaChr7	1890118	(GTT)5	GATTGGAATTAACAGCCGGA	TTTGATGGCGGCACTAAAAC	59.9	239	CAREOSREP1	CAACTC	Motif found in REP-1 gene promoter	BNR repeat
Ca-CNMS528	28367245	CaChr7	2041125	(TC)6	AAATAAGGCCACATGCCAAA	GCTGATGGACAAATGTGTGG	60.3	270	CTRMCAMV35S	TCTCTCTCT	CaMV 35S (Inverted GAGA) found in a 60-nucleotide region downstream of the transcription start site of the	Allergen V5/Tpx-1-related
Ca-CNMS529	28367246	CaChr7	2358889	(TC)7	ACAATTCCAATTTCCAACCG	GGAGTTGAGGTGCTTTGCTT	59.7	208	CTRMCAMV35S	TCTCTCTCT	CaMV 35S (Inverted GAGA) found in a 60-nucleotide region downstream of the transcription start site of the	Fibronectin, type III
Ca-CNMS530	28367248	CaChr7	2937044	(TTTA)5	GCGATTCCCACAATACTGAAA	CGTGCCTTCAAATACGTGTC	60.0	239	TATABOX5	TTATTT	TATA box found in the 5'upstream region of pea glutamine synthetase gene	Protein kinase, catalytic domain
Ca-CNMS531	28367249	CaChr7	3179743	(AG)6	ATTCTGTGCCACTCACATCG	AAAAACAGCCTTTTTCCCGT	59.7	159	CTRMCAMV35S	TCTCTCTCT	CaMV 35S (Inverted GAGA) found in a 60-nucleotide region downstream of the transcription start site of the	Acyl-ACP thioesterase
Ca-CNMS532	28367250	CaChr7	3210133	(TAC)5	ACTTCCCATTCTTCCACACG	ATGCAACAAGCCATGATGAG	60.0	236	CACTFTPPCA1	YACT	Tetranucleotide (CACT) is a key component of Mem1 found in the cis-regulatory element in the distal region of	Peptidase, cysteine peptidase active site
Ca-CNMS533	28367251	CaChr7	3539944	(CT)7	TCGGATAATCCAACTCCGTC	GAAAACGAAAAGGGGGAGAA	59.9	159	CTRMCAMV35S	TCTCTCTCT	CaMV 35S (Inverted GAGA) found in a 60-nucleotide region downstream of the transcription start site of the	Zinc finger, FYVE-type
Ca-CNMS534	28367252	CaChr7	3780661	(TTTA)6	CATCCATGCAACAAGTCACTG	TTAAGAATAACGGCACATATCTTG	60.2	280	TATABOX5	TTATTT	TATA box found in the 5'upstream region of pea glutamine synthetase gene	Glutathione S-transferase, N-terminal
Ca-CNMS535	28367253	CaChr7	3789472	(CAA)5	TATGATGGAACCAGAAGAGTG	CAGTGTTGTTTGTTCCTGATT	55.0	150	RAV1AAT	CAACA	AP2 & B3 like domians in RAV1	Chromatin remodeling factor18 (CHR18)
Ca-CNMS536	28367254	CaChr7	3789472	(CATTCT)3	TCCTTCTTCTTCTCAACAACA	TTGCAGAATGAGAATGAGAAT	55.0	139	INRNTPSADB	YTCANTYY	Found in tobacco Psb gene promoter without TATA box	Chromatin remodeling factor18 (CHR18)
Ca-CNMS537	28367255	CaChr7	4295527	(CT)6	ACCCAAATCCATTCAAACCC	ACACCTACACAGTTGCGACG	60.8	204	CTRMCAMV35S	TCTCTCTCT	CaMV 35S (Inverted GAGA) found in a 60-nucleotide region downstream of the transcription start site of the	Integrase, catalytic core
Ca-CNMS538	28367256	CaChr7	4598241	(CT)7	TTCTCACTCATGCTCTTTCTC	TCTAACACAAACACGATTTCA	55.0	150	CTRMCAMV35S	TCTCTCTCT	CaMV 35S (Inverted GAGA) found in a 60-nucleotide region downstream of the transcription start site of the	GATA factor family of zinc finger transcription factors
Ca-CNMS539	28367257	CaChr7	4624457	(AG)8	GAAGTTCTGCCACTGAGTTC	TTAGGAACAGCCTTGTCAAT	55.0	153	GAGA8HVBKN3	(GA)8	Motif found in intron IV of the barley (H.v.) gene Bkn3	Pollen-specific transcription factor
Ca-CNMS540	28367259	CaChr7	4870237	(CGTAA)4	GAATGTTGTTGATAGGTTGGA	TGATTGATGGAATAAGGAGAG	55.0	142	GARE2OSREP1	TAACGTA	GARE found in REP-1 gene promoter in RICE	BTB/POZ domain-containing protein
Ca-CNMS541	28367260	CaChr7	5352320	(CT)8	AACGTGTGAATGAAGGAACTA	GGGAGAGAAGAGAAGAGAGAA	55.0	154	CTRMCAMV35S35S	TCTCTCTCT	CaMV 35S (Inverted GAGA) found in a 60-nucleotide region downstream of the transcription start site of the	Zinc finger C-x8-C-x5-C-x3-H type family protein
Ca-CNMS542	28367261	CaChr7	5352320	(TCTCT)3	AACACACAAACACAGTTCTCC	AGTGTGATTGAATTGAGGTTG	55.0	154	DOFCOREZM	AAAG	Site required for binding of Dof proteins in maize	Zinc finger C-x8-C-x5-C-x3-H type family protein
Ca-CNMS543	28367262	CaChr7	5565344	(TAGT)5	TTGGAAATGAACATGCCAGA	TGCAGAATTTATAACTCTGTTGCTC	60.0	170	MYB1LEPR	GTTAGTT	Tomato Pti4(ERF) regulates defence-related gene expression via GCC box and non-GCC box cis elements	Metridin-like ShK toxin
Ca-CNMS544	28367263	CaChr7	6052379	(CT)11	TCTCTCATCCTCTTTCTTTCC	GATCCAAAGAATGGAATAACC	55.0	147	CTRMCAMV35S35S	TCTCTCTCT	CaMV 35S (Inverted GAGA) found in a 60-nucleotide region downstream of the transcription start site of the	KH domain-containing protein / zinc finger (CCCH type) family
Ca-CNMS545	28367264	CaChr7	6054880	(CAA)7	CAAGCTCACACTGAACTCTCT	GTTGTTGAGTTGTGGTGATTT	55.0	146	RAV1AAT	CAACA	AP2 & B3 like domians in RAV1	KANADI protein (KAN)
Ca-CNMS546	28367265	CaChr7	6057925	(TC)7	TGAGAACAAGACCAGAGTCAC	GTGTTATTGTTGGACCTCAAG	55.0	144	CTRMCAMV35S35S	TCTCTCTCT	CaMV 35S (Inverted GAGA) found in a 60-nucleotide region downstream of the transcription start site of the	Possible function in phloem development in the root
Ca-CNMS547	28367266	CaChr7	6060830	(CT)10	ATTCTCCTCATAAGCCATTCT	TTCTCGATCTATACGTCAACC	55.0	150	CTRMCAMV35S35S	TCTCTCTCT	CaMV 35S (Inverted GAGA) found in a 60-nucleotide region downstream of the transcription start site of the	A Class II KN1-like homeodomain transcription factors
Ca-CNMS548	28367267	CaChr7	6061925	(TCT)5	ATTCCCTTAATTCTTCCGTTT	AGAATGGCTTATGAGGAGAAT	56.0	151	NODCON2GM	CTCTT	Putative nodulin consensus sequences	A Class II KN1-like homeodomain transcription factors
Ca-CNMS549	28367268	CaChr7	6367673	(GA)6	ACTGGAAGGAAACGGGAGTT	GGAACAAAGAACTGTAAGGGCA	60.0	272	CTRMCAMV35S	TCTCTCTCT	CaMV 35S (Inverted GAGA) found in a 60-nucleotide region downstream of the transcription start site of the	AIG1
Ca-CNMS550	28367270	CaChr7	6388656	(CT)6	GCATGCTTCATTTCACGCTA	TCGGAGAATGATAAGCAAAGG	60.0	109	CTRMCAMV35S	TCTCTCTCT	CaMV 35S (Inverted GAGA) found in a 60-nucleotide region downstream of the transcription start site of the	Metallo-dependent phosphatase
Ca-CNMS551	28367271	CaChr7	6487370	(CT)10	AACCAAACCAAGCAGCTCAT	GGAGTGGGTTCTTCAGAGACA	59.7	110	CTRMCAMV35S	TCTCTCTCT	CaMV 35S (Inverted GAGA) found in a 60-nucleotide region downstream of the transcription start site of the	ANTH
Ca-CNMS552	28367272	CaChr7	6931106	(CT)7	TTCAATGTGTGTTTCATTGGC	GGTGATCCGCAAAACAGAAT	59.4	265	CTRMCAMV35S	TCTCTCTCT	CaMV 35S (Inverted GAGA) found in a 60-nucleotide region downstream of the transcription start site of the	RHO protein GDP dissociation inhibitor
Ca-CNMS553	28367273	CaChr7	7923780	(TTTATA)10	CCACTTTTTGCCATTTAGCTT	GAAATTCATTTAAATTCTGTCCAAA	58.4	231	ROOTMOTIFTAPOX1	ATATT	Motif found both in promoters of rolD	Peptidase T2, asparaginase 2
Ca-CNMS554	28367274	CaChr7	8290354	(TGA)7	ACGCTGATGATGATCGTGAA	TGTGATGCAACTAAGCCACC	60.2	235	CTRMCAMV35S	TCTCTCTCT	CaMV 35S (Inverted GAGA) found in a 60-nucleotide region downstream of the transcription start site of the	Calmodulin-binding protein, plant
Ca-CNMS555	28367275	CaChr7	8462951	(GGTGTT)3	GAGAAGAAATGGAGGGAGATA	CCACTTTGATTGACACTACCA	55.0	169	POLLENILELAT52	AGAAA	One of two co-dependent regulatory elements responsible for pollen specific activation of tomato lat52	NAC domain containing protein 100
Ca-CNMS556	28367276	CaChr7	8462951	(TAG)6	ATTTGGATGATAAGTGGGATT	ATATCATCACTTTCCCAATCA	55.0	148	CACTFTPPCA1	YACT	Tetranucleotide (CACT) is a key component of Mem1 found in the cis-regulatory element in the distal region of	NAC domain containing protein 100 (NAC100)
Ca-CNMS557	28367277	CaChr7	8513751	(TC)18	TGAGCGTCCAGCTTAGGAGT	ATTCGGAGGAAGGGTGAGAT	60.2	275	CTRMCAMV35S	TCTCTCTCT	CaMV 35S (Inverted GAGA) found in a 60-nucleotide region downstream of the transcription start site of the	Lactate/malate dehydrogenase, N-terminal

Marker IDs	NCBI Probe IDs (PUIDs)	Chromosomes	Physical positions (bp)	Microsatellite repeat-motifs	Forward Primer (5'-3')	Reverse Primer (5'-3')	Actual annealing tempearture (0C)	Size (bp) of alleles amplified	Regulatory elements	Signal sequences of known regulatory element-binding sites	Function of known regulatory elements/ transcription factor-binding sites	Putative gene function
Ca-CNMS558	28367278	CaChr7	8721514	(AG)7	TCGAATCGAAGGATTGAAGG	CTTTTTCTCAGCATAGCCGC	60.1	117	CTRMCAMV35S	TCTCTCTCT	CaMV 35S (Inverted GAGA) found in a 60-nucleotide region downstream of the transcription start site of the	Ubiquitin-conjugating enzyme, E2
Ca-CNMS559	28367279	CaChr7	8848669	(AAC)5	TTGCCAATCAGGAGCCTTAC	TCTTGGGTTATGGAAGCAGC	60.2	197	RAV1AAT	CAACA	AP2 & B3 like domiane in RAV1	ATPase, P-type, H+ transporting proton pump
Ca-CNMS560	28367281	CaChr7	10684193	(ACT)6	CTCTCTTGTCCTGTGGTCCG	TTCCTTGAACAAAAACATGGG	60.9	169	CACTFTPPCA1	YACT	Tetranucleotide (CACT) is a key component of Mem1 found in the cis-regulatory element in the distal region of	Signal transduction response regulator, receiver domain
Ca-CNMS561	28367282	CaChr7	10685193	(TC)8	GCCATTCTTCACTCTTTCTTT	GTTCAAATCCGAAACGTAAAT	55.0	148	CTRMCAMV35S	TCTCTCTCT	CaMV 35S (Inverted GAGA) found in a 60-nucleotide region downstream of the transcription start site of the	HSI2-like 1 (HSL1)
Ca-CNMS562	28367283	CaChr7	12021223	(ACACAA)4	CCTCTCTTATTTTGTTTGTCAAC	TTCCTTCGAAATTGAGTAAAG	55.0	165	CANBNNAPA	CNAACAC	Core of "(CA)n element" in storage protein genes in Brasica napus embryo- and endosperm-specific	WRKY DNA-binding protein 15 (WRKY15)
Ca-CNMS563	28367284	CaChr7	12120636	(AG)8	TCGTTGCACTGTCTATTTCCA	TTCCCACGTTAACCAAATCG	59.3	152	CTRMCAMV35S	TCTCTCTCT	CaMV 35S (Inverted GAGA) found in a 60-nucleotide region downstream of the transcription start site of the	Pathogenesis-related transcriptional factor/ERF, DNA-
Ca-CNMS564	28367285	CaChr7	12120636	(GA)12	TGACGAGTCATTGAAATTGAAAA	ACAGCATAGACGAGGCCAGT	59.6	143	GAGA8HVBKN3	GAGAGAGAGAGAGAGA	Motif found in intron IV of the barley (H.v.) gene Bkn3	Pathogenesis-related transcriptional factor/ERF, DNA-
Ca-CNMS565	28367286	CaChr7	12489499	(AAC)5	CAAGGTGGGCTTAGCTTTTG	TGGTGGTAGCTAAGAAATGTAGC	59.9	271	RAV1AAT	CAACA	AP2 & B3 like domiane in RAV1	Transposase, MuDR, plant
Ca-CNMS566	28367287	CaChr7	12892443	(AG)22	GTGTGTCTTGATGTGGGTCG	CCCAAAAAATTTCAACTCCCA	60.0	279	GAGA8HVBKN3	GAGAGAGAGAGAGAGA	Motif found in intron IV of the barley (H.v.) gene Bkn3	Peptidase S9, prolyl oligopeptidase, catalytic domain
Ca-CNMS567	28367288	CaChr7	12910310	(GA)6	ATGACTTCACCCTGTGGGTC	TCGAGAAAGAAAAAGGAGAAAA	59.8	212	GAGA8HVBKN3	GAGAGAGAGAGAGAGA	Motif found in intron IV of the barley (H.v.) gene Bkn3	YbaK/aminoacyl-tRNA synthetase-associated domain
Ca-CNMS568	28367289	CaChr7	13371188	(GA)10	GACACCCACGTGGCATAAG	AGCAAATGATGGAGTGTCCC	60.0	101	GAGA8HVBKN3	GAGAGAGAGAGAGAGA	Motif found in intron IV of the barley (H.v.) gene Bkn3	Armadillo
Ca-CNMS569	28367290	CaChr7	13903965	(GAA)4	TGAATTCACGGACTATGTTTT	ATCCGTTTGCTATTGATCTCT	55.0	150	POLLENILELAT52	AGAAA	One of two co-dependent regulatory elements responsible for pollen specific activation of tomato lat52	TLP family
Ca-CNMS570	28367292	CaChr7	15804254	(AG)10	CCATTGCAACATTGCATACAT	TGCTTTAAATGCTGTTGAGGTT	59.3	270	GAGA8HVBKN3	GAGAGAGAGAGAGAGA	Motif found in intron IV of the barley (H.v.) gene Bkn3	Protein kinase, catalytic domain
Ca-CNMS571	28367293	CaChr7	16214715	(AG)6	TGGTGCATGAACACTAGGGA	TCCCAACTCAATTACATTAGAGCA	60.1	242	CTRMCAMV35S	TCTCTCTCT	CaMV 35S (Inverted GAGA) found in a 60-nucleotide region downstream of the transcription start site of the	Auxin efflux carrier
Ca-CNMS572	28367294	CaChr7	16667381	(TC)6	ATTTTTCCTTCCCTTCCCCT	GCGGTTAGGGTTTGGTGTTA	60.1	130	CTRMCAMV35S	TCTCTCTCT	CaMV 35S (Inverted GAGA) found in a 60-nucleotide region downstream of the transcription start site of the	Proteasome, alpha-subunit, conserved site
Ca-CNMS573	28367295	CaChr7	19143770	(TTG)6	AGGCTTTGGTTCGGTTCTTT	AAACTCCCCAAAAATGCACA	60.1	118	CAREOSREP1	CAACTC	Motif found in REP-1 gene promoter	Protein-tyrosine phosphatase-like, PTPLA
Ca-CNMS574	28367296	CaChr7	19467984	(CT)6	TCTCAAATTCAACCTAAATGC	CTGCTATCCACCTCTTTCAC	55.0	146	CTRMCAMV35S	TCTCTCTCT	CaMV 35S (Inverted GAGA) found in a 60-nucleotide region downstream of the transcription start site of the	MYC-related transcriptional activator
Ca-CNMS575	28367297	CaChr7	19467984	(CT)6	ACTCCTAAACCTTTTTGTTGC	GGCAGAGAGAAAGAGAGAGAG	55.0	167	CTRMCAMV35S	TCTCTCTCT	CaMV 35S (Inverted GAGA) found in a 60-nucleotide region downstream of the transcription start site of the	Auxin (indole-3-acetic acid) induced gene
Ca-CNMS576	28367298	CaChr7	19467984	(TCTCTT)3	TTGTGTTTGTGTTTAAGAGCA	GGTGTGTAGCAGAAGAGAAGA	55.0	142	NODCON2GM	CTCTT	Putative nodulin consensus sequences	Auxin (indole-3-acetic acid) induced gene
Ca-CNMS577	28367299	CaChr7	19619642	(GAA)18	AACTTGTCGAAGAAAGACTCC	ATTCAATCACTCATCACCATC	55.0	155	TL1ATSAR	CTGAAGAAGAA	Sequence overrepresented in the promoter regions of all 13 NPR1-responsive ER-resident genes	AtbZIP60 consists of a bZIP DNA binding domain
Ca-CNMS578	28367300	CaChr7	19831513	(CT)18	ACCGCCTCAATTTCTTCTTA	TAATTTCACGGACAGTGTCTT	57.0	148	CTRMCAMV35S	TCTCTCTCT	CaMV 35S (Inverted GAGA) found in a 60-nucleotide region downstream of the transcription start site of the	PLATZ transcription factor family protein
Ca-CNMS579	28367301	CaChr7	20119878	(TTG)6	TGAAACAAAACAAAATTGAGAGC	CAAATCTGGTTGTGCTGGAA	58.5	230	CAREOSREP1	CAACTC	Motif found in REP-1 gene promoter	Myb, DNA-binding
Ca-CNMS580	28367303	CaChr7	20120878	(CGGTT)3	CTGTTCTCTCGGTGACATCT	ATTGTTTTCACAGAGCTTCAA	55.0	149	MYBCOREATCYCB1	AACGG	Motif found in the promoter of Arabidopsis thaliana cyclin B1:1 gene	Nuclear factor Y, subunit B2 (NF-YB2)
Ca-CNMS581	28367304	CaChr7	20231269	(TAT)6	GGTTTGTTCCCTTTTACTTGT	ACGTCTTCAAAATAGGGAAAT	55.0	141	ROOTMOTIFTAPOX1	ATATT	Motif found both in promoters of rolD	Floral homeotic gene encoding a MADS domain protein
Ca-CNMS582	28367305	CaChr7	20365885	(CT)7	AGTGGACGCATGAAGCATTT	GGTCAGAATATGCAGAAGGCA	60.7	271	CTRMCAMV35S	TCTCTCTCT	CaMV 35S (Inverted GAGA) found in a 60-nucleotide region downstream of the transcription start site of the	Helix-loop-helix DNA-binding domain
Ca-CNMS583	28367306	CaChr7	21803338	(GA)6	TGTTTAGCTGCATGAAGTTGT	CTTCACTCTTCACTCTCCTCA	56.0	148	CTRMCAMV35S	TCTCTCTCT	CaMV 35S (Inverted GAGA) found in a 60-nucleotide region downstream of the transcription start site of the	ERF (ethylene response factor) subfamily B-5 of ERF/AP2
Ca-CNMS584	28367307	CaChr7	23289436	(TATT)5	CGAAATTAAACTTCTTTAGGCCG	GGTCCTCAAGACACCAAAGG	60.4	116	TATABOX5	TTATTT	TATA box found in the 5'upstream region of pea glutamine synthetase gene	Lipocalin, bacterial
Ca-CNMS585	28367308	CaChr7	23969048	(AGC)5	TTCATTTGTACAATGGGCGA	AATCTGGATCGGGGAAAATC	59.9	143	ANAERO2CONSENSUS	AGCAGC	Motif found in silico in promoters of 13 anaerobic genes involved in the fermentative pathway	ZF-HD homeobox protein, Cys/His-rich dimerisation domain
Ca-CNMS586	28367309	CaChr7	24020862	(CT)6	TCAACCCTACAACACTTCATC	ATTTCCAAGTGCTAAAAGCAT	55.0	132	CTRMCAMV35S	TCTCTCTCT	CaMV 35S (Inverted GAGA) found in a 60-nucleotide region downstream of the transcription start site of the	Homeobox protein 34 (HB34)
Ca-CNMS587	28367310	CaChr7	27128667	(GA)7	TTTGTCTCAACAACCTCCCC	GGGGGAGCATAAACATTTCA	59.9	125	CTRMCAMV35S	TCTCTCTCT	CaMV 35S (Inverted GAGA) found in a 60-nucleotide region downstream of the transcription start site of the	Bromo adjacent homology (BAH) domain
Ca-CNMS588	28367311	CaChr7	27235472	(TA)6	GGAAGAAATCAATGAAAAACC	AGGCCAAATTTCTTGTACTCT	55.0	159	TATAPVTRNALEU	TTTATATA	Motif found in Phaseolus vulgaris tRNALeu gene promoter	A class I knotted1-like homeobox gene family (together with KNAT2)
Ca-CNMS589	28367312	CaChr7	27853602	(ATTT)5	TGCCATGTGACCTCCACTAA	TGGCTATTGAATCCATGTTGTG	60.1	254	TATABOX5	TTATTT	TATA box found in the 5'upstream region of pea glutamine synthetase gene	Pseudouridine synthase/archaeosine
Ca-CNMS590	28367314	CaChr7	28548137	(AG)6	TGAGAATGATTTTGAAGAGGA	TTCCATTCTACATTCCACAGT	55.0	153	CTRMCAMV35S	TCTCTCTCT	CaMV 35S (Inverted GAGA) found in a 60-nucleotide region downstream of the transcription start site of the	Basic helix-loop-helix (bHLH) protein
Ca-CNMS591	28367315	CaChr7	28548137	(TC)9	TTCAGAAACGAGAGCAGACTA	ACTCACACTTGCACTCTCACT	56.0	161	CTRMCAMV35S	TCTCTCTCT	CaMV 35S (Inverted GAGA) found in a 60-nucleotide region downstream of the transcription start site of the	SBP-box gene, a the SPL gene family
Ca-CNMS592	28367316	CaChr7	28548137	(TC)7	GCGCTTAAAAGATCTCACTTT	TCGTTTTGTCTGGTACTTGAT	56.0	148	CTRMCAMV35S	TCTCTCTCT	CaMV 35S (Inverted GAGA) found in a 60-nucleotide region downstream of the transcription start site of the	SBP-box gene, a the SPL gene family
Ca-CNMS593	28367317	CaChr7	29656694	(AGC)5	ACACGCCCGTAATTAGCAAG	CCGCAAAGGTTTGTCATTTT	60.2	263	ANAERO2CONSENSUS	AGCAGC	Motif found in silico in promoters of 13 anaerobic genes involved in the fermentative pathway	Peptidase C48, SUMO/Sentrin/Ubl1
Ca-CNMS594	28367318	CaChr7	30194294	(CG)6	ACTCAACCCACCGCAAATAG	GGATGGAGTGGGAGGGTAAT	60.0	270	CGCGBOXAT	VCGCGB	Recognized by AtSR1-6 signal-responsive genes	Protein of unknown function DUF1675
Ca-CNMS595	28367319	CaChr7	30760343	(TCTT)4	ACACAAAATTCACAACACCTC	AATGCTTATTACCATCCTTGC	55.0	150	NODCON2GM	CTCTT	Putative nodulin consensus sequences	Class III HD-ZIP family protein
Ca-CNMS596	28367320	CaChr7	30760343	(TGG)5	CAAAATGCGTAGTGAGTCTTC	AAGGAAAGTGATGGAAATAAGA	55.0	149	CACTFTPPCA1	YACT	Tetranucleotide (CACT) is a key component of Mem1 found in the cis-regulatory element in the distal region of	Class III HD-ZIP family protein
Ca-CNMS597	28367321	CaChr7	31252896	(TTTA)5	AGAATGAATTCGGCGCTCT	TGTCACAGAAAGTTAGCAGAGAGAA	59.9	279	TATABOX5	TTATTT	TATA box found in the 5'upstream region of pea glutamine synthetase gene	IQ motif, EF-hand binding site
Ca-CNMS598	28367322	CaChr7	31413220	(AG)11	TCCCTGTCATGAACTTTCCC	AGTGGTGTGTGTGGATTGTGA	59.9	268	GAGA8HVBKN3	GAGAGAGAGAGAGAGA	Motif found in intron IV of the barley (H.v.) gene Bkn3	Glycosyl transferase, family 14
Ca-CNMS599	28367323	CaChr7	31541054	(CAA)4	GAAATGGGTTTTATGGCTTAT	CACCTTCAAAATCACTCAGTC	55.0	142	RAV1AAT	CAACA	AP2 & B3 like domiane in RAV1	Rop subfamily of Rho GTPases
Ca-CNMS600	28367326	CaChr7	31541054	(CAA)4	GAAATGGGTTTTATGGCTTAT	CACCTTCAAAATCACTCAGTC	55.0	142	RAV1AAT	CAACA	AP2 & B3 like domians in RAV1	A ROP GTPase gene family.

Marker IDs	NCBI Probe IDs (PUIDs)	Chromosomes	Physical positions (bp)	Microsatellite repeat-motifs	Forward Primer (5'-3')	Reverse Primer (5'-3')	Actual annealing tempearture (0C)	Size (bp) of alleles amplified	Regulatory elements	Signal sequences of known regulatory element-binding sites	Function of known regulatory elements/ transcription factor-binding sites	Putative gene function
Ca-CNMS601	28367327	CaChr7	31541054	(TC)14	GGTAGAGGATGAGGATGAGTT	TTTGTTGGATGTTAAAGGTTG	55.0	151	CTRMCAMV35S	TCTCTCTCT	CaMV 35S (Inverted GAGA) found in a 60-nucleotide region downstream of the transcription start site of the	A ROP GTPase gene family.
Ca-CNMS602	28367328	CaChr7	31541054	(TC)14	GGTAGAGGATGAGGATGAGTT	TTTGTTGGATGTTAAAGGTTG	55.0	151	CTRMCAMV35S	TCTCTCTCT	CaMV 35S (Inverted GAGA) found in a 60-nucleotide region downstream of the transcription start site of the	Rop subfamily of Rho GTPases
Ca-CNMS603	28367329	CaChr7	31541054	(AGA)4	AGTTACAATTGATGGTGATGG	AAAAGGTATTGGACAAAAGGT	55.0	138	TCA1MOTIF	TCATCTTCTT	Related to salicylic acid-inducible expression of many genes	A ROP GTPase gene family
Ca-CNMS604	28367330	CaChr7	31872063	(TTG)5	GTTCCTGTTGAGGCACCATT	TGTTCATCAACAACTTGCTCTTT	60.0	182	CAREOSREP1	CAACTC	Motif found in REP-1 gene promoter	Lateral organ boundaries, LOB
Ca-CNMS605	28367331	CaChr7	32383066	(TC)7	ATAAAATGAAAACGGAGTCGT	AAACACAAGTCTTGATTTCTCA	56.0	139	-10PEHVPSBD	TATTCT	Motif found in the barley (H.v.) chloroplast psbD gene promoter	AL5, Alfin-Like family protein containing PhD domain
Ca-CNMS606	28367332	CaChr7	32383066	(TTC)4	CTGAAAACCTCTTCAACAGTG	ATCTCTTTCAACCCTAAAACG	55.0	156	-10PEHVPSBD	TATTCT	Motif found in the barley (H.v.) chloroplast psbD gene promoter	AL5, Alfin-Like family protein containing PhD domain
Ca-CNMS607	28367333	CaChr7	32837971	(CT)11	GAGGAGTGATGACAGGTGGG	GAGAAGGGGGTGAATGGAAT	60.5	178	CTRMCAMV35S	TCTCTCTCT	CaMV 35S (Inverted GAGA) found in a 60-nucleotide region downstream of the transcription start site of the	NA
Ca-CNMS608	28367334	CaChr7	32855564	(TTG)7	GGGGTTTTGGTAAGATTGTGAA	TCAAGAAAGCCTGTGGAACC	60.1	249	CAREOSREP1	CAACTC	Motif found in REP-1 gene promoter	WRC
Ca-CNMS609	28367335	CaChr7	33279606	(ACT)5	ATCAATGCCGCATCTCTTTT	TTTGGTGAGAAACATGGACAA	59.7	128	CACTFTPPCA1	YACT	Tetranucleotide (CACT) is a key component of Mem1 found in the cis-regulatory element in the distal region of	Protein kinase, catalytic domain
Ca-CNMS610	28367337	CaChr7	34236278	(CAA)5	TCCCTCCTTTTGCAAGATTC	TCTGGTGAATTGCCATCTTT	59.2	130	RAV1AAT	CAACA	AP2 & B3 like domiane in RAV1	Protein kinase, catalytic domain
Ca-CNMS611	28367338	CaChr7	35086618	(TCT)6	ACGAAATCCAACGTATACAAA	TCCTTGAAGAATCCACAAATA	55.0	153	CTRMCAMV35S	TCTCTCTCT	CaMV 35S (Inverted GAGA) found in a 60-nucleotide region downstream of the transcription start site of the	Auxin response factor 9 (ARF9)
Ca-CNMS612	28367339	CaChr7	35086618	(CTTT)3	TTCATTAGTCACAAGCAAACA	GAAAATGGTTGATCAGTTGAA	55.0	151	DOFCOREZM	AAAG	Site required for binding of Dof proteins in maize	Pseudo-response regulator 2 (APRR2)
Ca-CNMS613	28367340	CaChr7	35889089	(TC)6	GGATGGACAATGTTTTTGTTTTT	TTAAATTGGATGCTTTGTTTTGT	59.2	240	CTRMCAMV35S	TCTCTCTCT	CaMV 35S (Inverted GAGA) found in a 60-nucleotide region downstream of the transcription start site of the	HhH-GPD domain
Ca-CNMS614	28367341	CaChr7	36331715	(AACA)5	GCAAAGCAAAATGTTCCCAT	AAACCGAGTTCGAAGGTTTG	59.9	201	AACACOREOSGLUB1	AACAAAC	Motif found in rice (O.s.) glutelin genes, involved in controlling the endosperm-specific expression	Protein kinase, catalytic domain
Ca-CNMS615	28367342	CaChr7	36358644	(AG)10	GGGTTGTGTGCACTTGTACCT	TGACCTCCTTGACCTTGGTT	59.9	218	GAGA8HVBKN3	GAGAGAGAGAGAGAGA	Motif found in intron IV of the barley (H.v.) gene Bkn3	ARID/BRIGHT DNA-binding domain
Ca-CNMS616	28367343	CaChr7	36409689	(CAG)4	AGTTAGGGTTTGAATCTTTGC	TGAGAAATTGTTACTCCCAAA	55.0	170	RAV1AAT	CAACA	AP2 & B3 like domians in RAV1	SWI/SNF chromatin remodeling ATPase
Ca-CNMS617	28367344	CaChr7	36409689	(CAA)4	AATTAGGCCTGATCTTGTGTT	ACACAGCCTAACAAACTTACG	56.0	154	ANAERO2CONSENSUS	AGCAGC	Motif found in silico in promoters of 13 anaerobic genes involved in the fermentative pathway	SWI/SNF chromatin remodeling ATPase
Ca-CNMS618	28367345	CaChr7	36409689	(GCT)4	GTAAGATAACAGCTGCCAAAA	CTCCAGAAAACGATTCCTAAT	55.0	155	ANAERO2CONSENSUS	AGCAGC	Motif found in silico in promoters of 13 anaerobic genes involved in the fermentative pathway	SWI/SNF chromatin remodeling ATPase
Ca-CNMS619	28367346	CaChr7	38216093	(ACT)5	AAAATCACCGTCACTCCAGC	AGGAGAAAGCGCAATTGAAA	60.1	211	CACTFTPPCA1	YACT	Tetranucleotide (CACT) is a key component of Mem1 found in the cis-regulatory element in the distal region of	Zinc finger, CCCH-type
Ca-CNMS620	28367348	CaChr7	39752897	(ACAA)5	CAATCGCATCTTTGTTTTAGGA	TCTGCCCATATTTTTCTGGA	59.3	280	AACACOREOSGLUB1	AACAAAC	Motif found in rice (O.s.) glutelin genes, involved in controlling the endosperm-specific expression	Integrase, catalytic core
Ca-CNMS621	28367349	CaChr7	39766430	(ATTTCA)3	TTCTTCCCAATTTCATTTAC	CTTTGGTTGAAATCAGAATGA	52.0	172	CAATBOX1	CAAT	CAAT promoter consensus sequence found in legA gene of pea	CCCH-type zinc finger protein with ARM repeat domain
Ca-CNMS622	28367350	CaChr7	39766430	(TCT)6	TCTTCCCAATTTCATTTACA	AACAATGAGATCTGGAACATC	53.0	140	NODCON2GM	CTCTT	One of two putative nodulin consensus sequences	CCCH-type zinc finger protein with ARM repeat domain
Ca-CNMS623	28367351	CaChr7	41350588	(AAC)5	TATCACCACCATCTCCACCA	GGATTGGTTAGGTGTCCGAA	59.8	217	RAV1AAT	CAACA	AP2 & B3 like domiane in RAV1	Glutaredoxin
Ca-CNMS624	28367352	CaChr7	41846301	(TC)7	CAAACTCTCCGATCCTTAAATCA	GAAAATCGGTCAACGGAAAA	59.6	149	CTRMCAMV35S	TCTCTCTCT	CaMV 35S (Inverted GAGA) found in a 60-nucleotide region downstream of the transcription start site of the	Peptidase A1
Ca-CNMS625	28367353	CaChr7	42101871	(CT)31	TGGGTTTGGTTTGGTTCATT	ACCTCGGAATTTTGATGACG	60.1	245	CTRMCAMV35S	TCTCTCTCT	CaMV 35S (Inverted GAGA) found in a 60-nucleotide region downstream of the transcription start site of the	Basic-leucine zipper (bZIP) transcription factor
Ca-CNMS626	28367354	CaChr7	46477036	(GTT)5	TGATTATTGAAATATTGTTGTTGCT	TTTCCTCCTGCATTTCATCA	57.0	227	CAREOSREP1	CAACTC	Motif found in REP-1 gene promoter	Protein kinase, catalytic domain
Ca-CNMS627	28367355	CaChr7	46618587	(AGC)5	TTCATTTGTACAATGGGCGA	AAAAAGGCAGCAGACCTCAA	59.9	238	ANAERO2CONSENSUS	AGCAGC	Motif found in silico in promoters of 13 anaerobic genes involved in the fermentative pathway	Peptidase C48, SUMO/Sentrin/Ubl1
Ca-CNMS628	28367356	CaChr7	47146804	(TC)6	TTTCCCCAAAAGCTGGACTA	GTTCGAGCCATGGTTGTTTT	59.7	100	CTRMCAMV35S	TCTCTCTCT	CaMV 35S (Inverted GAGA) found in a 60-nucleotide region downstream of the transcription start site of the	NA
Ca-CNMS629	28367357	CaChr7	47643175	(TC)6	GAAGCTGGAGTGCTGACACA	TGGTGAAGAAGATGAACAGTGAA	60.2	226	CTRMCAMV35S	TCTCTCTCT	CaMV 35S (Inverted GAGA) found in a 60-nucleotide region downstream of the transcription start site of the	Pathogenesis-related transcriptional factor/ERF, DNA-
Ca-CNMS630	28367359	CaChr8	1261671	(AG)6	CTGAGAAATCTACCGCTCTG	CGTGAGTTTTGGTAATGAGTC	55.0	147	CTRMCAMV35S	TCTCTCTCT	CaMV 35S (Inverted GAGA) found in a 60-nucleotide region downstream of the transcription start site of the	CCCH-type zinc fingerfamily protein with RNA-binding domain
Ca-CNMS631	28367360	CaChr8	1261671	(TAAGAG)3	CTGAGAAATCTACCGCTCTG	CAACATACCTTACCAGTAGCC	55.0	147	CACTFTPPCA1	YACT	Tetranucleotide (CACT) is a key component of Mem1 found in the cis-regulatory element in the distal region of	CCCH-type zinc fingerfamily protein with RNA-binding domain
Ca-CNMS632	28367361	CaChr8	1743959	(ACT)6	CTAGGTTTACCCTTTCCCATA	TTGACTCTTCTTCTTCAAACG	55.0	161	CACTFTPPCA1	YACT	Tetranucleotide (CACT) is a key component of Mem1 found in the cis-regulatory element in the distal region of	Transcription factor jumonji (jmjC) domain-containing protein
Ca-CNMS633	28367362	CaChr8	2394632	(GA)6	TTGTGTACCCCACTCTGCAA	AGTAAAGCTCCTGCATTGGC	60.2	261	CTRMCAMV35S	TCTCTCTCT	CaMV 35S (Inverted GAGA) found in a 60-nucleotide region downstream of the transcription start site of the	SWIB/MDM2 domain
Ca-CNMS634	28367363	CaChr8	2528722	(ATGC)5	AAAGAGGGAAGTGGAACCAA	TGTTCTCCACAAATCACAATCC	58.6	279	RYREPEATBNNAPA	CATGCA	Transactivation of the Brassica napus napin promoter by ABI3	Leucine-rich repeat
Ca-CNMS635	28367364	CaChr8	2773050	(CAA)5	GGTTGCAAAGAAAGCAAAGC	GTGAATGAAGAAGGCGTTGG	60.0	135	RAV1AAT	CAACA	AP2 & B3 like domiane in RAV1	Protein of unknown function DUF581
Ca-CNMS636	28367365	CaChr8	3457000	(GCG)4	TATATATGGCCATGAAGTTCG	AGATAAGGGTTTTGAGGAATG	55.0	140	CGCGBOXAT	VCGCGB	Recognized by AtSR1-6 signal-responsive genes	N-acetyl-l-glutamate synthase 2 (NAGS2)
Ca-CNMS637	28367366	CaChr8	3609212	(GTT)7	GGAAGGTTCCACAGTTCCAA	ACGTTAGGAATGTTGTCGGC	59.9	198	CAREOSREP1	CAACTC	Motif found in REP-1 gene promoter	Actin-binding FH2/DRF autoregulatory
Ca-CNMS638	28367367	CaChr8	3997981	(AG)6	CACCTTCCATCTTCTTCTTCT	AAGCTACTGAAGTTGGTGCTA	55.0	155	CTRMCAMV35S	TCTCTCTCT	CaMV 35S (Inverted GAGA) found in a 60-nucleotide region downstream of the transcription start site of the	DREB subfamily A-5 of ERF/AP2 transcription factor family
Ca-CNMS639	28367368	CaChr8	3997981	(CT)6	TGACAAACTTAATGGGAGAGA	TCAACGACACTTCTCTTTCTC	55.0	154	CTRMCAMV35S	TCTCTCTCT	CaMV 35S (Inverted GAGA) found in a 60-nucleotide region downstream of the transcription start site of the	DREB subfamily A-5 of ERF/AP2 transcription factor family
Ca-CNMS640	28367370	CaChr8	3997981	(TC)9	CACCTTCCATCTTCTTCTTCT	TGACAAACTTAATGGGAGAGA	55.0	155	CTRMCAMV35S	TCTCTCTCT	CaMV 35S (Inverted GAGA) found in a 60-nucleotide region downstream of the transcription start site of the	DREB subfamily A-5 of ERF/AP2 transcription factor family
Ca-CNMS641	28367371	CaChr8	3997981	(AG)9	TGACAAACTTAATGGGAGAGA	CACCTTCCATCTTCTTCTTCT	55.0	154	GAGA8HVBKN3	(GA)8	Motif found in intron IV of the barley (H.v.) gene Bkn3	DREB subfamily A-5 of ERF/AP2 transcription factor family
Ca-CNMS642	28367372	CaChr8	3997981	(AAG)4	ACATAACAACAAACCCTTTCA	CAGGAATCTTGTTCAAGTCAA	55.0	154	DOFCOREZM	AAAG	Site required for binding of Dof proteins in maize	DREB subfamily A-5 of ERF/AP2 transcription factor family
Ca-CNMS643	28367373	CaChr8	4019973	(ACTTTC)3	GAGAAGGGGACTGTGAACTAC	GTTGAAGGTTGAAGAAAGAAA	55.0	162	INRNTPSADB	YTCANTYY	Motif found in the tobacco psaDb gene promoter without TATA boxes	Histone acetyltransferase

Marker IDs	NCBI Probe IDs (PUIDs)	Chromosomes	Physical positions (bp)	Microsatellite repeat-motifs	Forward Primer (5'-3')	Reverse Primer (5'-3')	Actual annealing tempearture (0C)	Size (bp) of alleles amplified	Regulatory elements	Signal sequences of known regulatory element-binding sites	Function of known regulatory elements/ transcription factor-binding sites	Putative gene function
Ca-CNMS644	28367374	CaChr8	4078811	(GTA)5	GGAATGTAATGGGATGTGGC	AAAGGATTTGTGTGGGGGTT	60.0	100	CACTFTPPCA1	YACT	Tetranucleotide (CACT) is a key component of Mem1 found in the cis-regulatory element in the distal region of	Helix-loop-helix DNA-binding domain
Ca-CNMS645	28367375	CaChr8	4405422	(CT)12	CGCTCTCATTCCTCAACACA	AAAAACAAATCGGAGCGTTG	60.0	265	CTRMCAMV35S	TCTCTCTCT	CaMV 35S (Inverted GAGA) found in a 60-nucleotide region downstream of the transcription start site of the	Lateral organ boundaries, LOB
Ca-CNMS646	28367376	CaChr8	4627430	(TGT)5	GGATGTGGAGTTGGAGGAGA	AAAACAACAACAATTGGTATGGTAAC	60.0	184	CAREOSREP1	CAACTC	Motif found in REP-1 gene promoter	Homeobox
Ca-CNMS647	28367377	CaChr8	4679425	(AGC)5	TTCATTTGTACAATGGGCGA	AAAAAGGCAGCAGACCTCAA	59.9	238	ANAERO2CONSENSUS	AGCAGC	Motif found in silico in promoters of 13 anaerobic genes involved in the fermentative pathway	Cytochrome P450
Ca-CNMS648	28367378	CaChr8	5411127	(AG)6	TGTTGAGTGGAGTGGAGTGG	GCCCACTTATCTGTCGCACT	59.7	249	CTRMCAMV35S	TCTCTCTCT	CaMV 35S (Inverted GAGA) found in a 60-nucleotide region downstream of the transcription start site of the	Protein of unknown function DUF702
Ca-CNMS649	28367379	CaChr8	5412127	(AG)6	TCTACAGACACAAAAGGGGTA	TCCTTCGTTATTGTTGTTGTT	55.0	150	GATABOX	GATA	GATA motif in CaMV 35S promoter required for high level, light regulated and tissue specific expression	SHI gene family protein
Ca-CNMS650	28367381	CaChr8	5412127	(TAG)5	GGGTTAAATTTGATTGGTTGT	CACTTATCTGTCGCACTCTTT	56.0	150	CACTFTPPCA1	YACT	Tetranucleotide (CACT) is a key component of Mem1 found in the cis-regulatory element in the distal region of	SHI gene family protein
Ca-CNMS651	28367382	CaChr8	5564048	(TTGAG)4	AATCCAATCGAAGAGAGAGAG	AGTTACCATCTCAATCCACAG	55.0	154	CAATBOX1	CAAT	CAAT promoter consensus sequence" found in legA gene of pea	DNA binding
Ca-CNMS652	28367383	CaChr8	5564048	(TCTT)3	ATACACCCTCAACTTTGGTTT	CAACAACAACAACACAAGCTA	55.0	153	POLLEN1LELAT52	AGAAA	One of two co-dependent regulatory elements responsible for pollen specific activation of tomato lat52	Basic helix loop helix domain protein
Ca-CNMS653	28367384	CaChr8	5568871	(AG)8	GGATGATACGCTTTAGGGCA	GCGATTGCGATGGTTTTAAT	60.1	262	CTRMCAMV35S	TCTCTCTCT	CaMV 35S (Inverted GAGA) found in a 60-nucleotide region downstream of the transcription start site of the	Zinc finger, CCCH-type
Ca-CNMS654	28367385	CaChr8	6995627	(CT)15	TGGTTTGTTAGCATTTTGCTTG	AAGAAGCGGGTATGTTCCCT	60.2	117	CTRMCAMV35S	TCTCTCTCT	CaMV 35S (Inverted GAGA) found in a 60-nucleotide region downstream of the transcription start site of the	Protein kinase, catalytic domain
Ca-CNMS655	28367386	CaChr8	7163805	(TCTCTT)3	AAGCTTTATGAAGGAGAGACAA	AAGCTCAGACACAACTGAGAC	55.0	133	NODCON2GM	CTCTT	Putative nodulin consensus sequences	Auxin response factor 9 (ARF9)
Ca-CNMS656	28367387	CaChr8	7842261	(TC)8	TCCTTGGTCACACCCTTTTC	GATTGGATCGGTGAAACGAC	59.9	137	CTRMCAMV35S	TCTCTCTCT	CaMV 35S (Inverted GAGA) found in a 60-nucleotide region downstream of the transcription start site of the	Pleckstrin homology domain
Ca-CNMS657	28367388	CaChr8	7868769	(GGA)5	ACGATCGTGGATAACGGAAC	GGATCTAAAACGACGTCATCA	59.8	271	UPRMOTIFIIAT	CCNNNNNNNNNNNNCCACG	conserved UPR (unfolded protein response) cis-acting element in Arabidopsis	UDP-glucuronosyl/UDP-glucosyltransferase
Ca-CNMS658	28367389	CaChr8	8639303	(AGA)4	TGAGATTGGTGTAAAGAAAGG	AATTTGTCCACCTTAGCCTAC	55.0	155	TCA1MOTIF	TCATCTTCTT	Related to salicylic acid-inducible expression of many genes	R2R3 factor gene family
Ca-CNMS659	28367390	CaChr8	8914578	(CATTT)3	TAACACCATCATCTCTCTCCA	TTTGGGTTTTGATTTGTGTAG	56.0	173	INRNTPSADB	YTCANTYY	Elements found in the tobacco psaDb gene promoter without TATA boxes	Multiprotein bridging factor 1
Ca-CNMS660	28367392	CaChr8	9319399	(CT)6	CAAACTGACACAACTGATTTGGA	GCTGCGTGTGTTGTGTAACTG	60.1	117	CTRMCAMV35S	TCTCTCTCT	CaMV 35S (Inverted GAGA) found in a 60-nucleotide region downstream of the transcription start site of the	Zinc finger, Dof-type
Ca-CNMS661	28367393	CaChr8	10605380	(TTG)5	CGACCGATGATCCTTTTCAT	AAGCAAATATCGCCAAAACG	59.9	271	CAREOSREP1	CAACTC	Motif found in REP-1 gene promoter	Protein kinase, catalytic domain
Ca-CNMS662	28367394	CaChr8	10605380	(AGT)5	CGTTTTGGCGATATTTGCTT	CTCGTCTCCTTTGCCTGATG	60.1	260	CACTFTPPCA1	YACT	Tetranucleotide (CACT) is a key component of Mem1 found in the cis-regulatory element in the distal region of	Protein kinase, catalytic domain
Ca-CNMS663	28367395	CaChr8	14435336	(TTG)5	CGGCGACATTTTCTTCAACT	AAGCATACCCATCGCTACAAA	60.2	263	CAREOSREP1	CAACTC	Motif found in REP-1 gene promoter	Ribosomal protein S7e
Ca-CNMS664	28367396	CaChr8	14983115	(TG)8	TGATGATTCTTCACTTTCTCC	ACTCATCAATCAAGAGGATCA	54.0	147	CANBNNAPA	CNAACAC	Element in storage protein genes in B napus embryo- and endosperm-specific transcription of napin	YABBY family of transcriptional regulators
Ca-CNMS665	28367397	CaChr8	15082918	(TCT)4	TCTAGCTTTGTTCCACAATTC	TTAATTCATGTTCCAACTTCC	55.0	133	ARR1AT	NGATT	Found in the promoter of rice NSHB gene	BSD domain-containing protein
Ca-CNMS666	28367398	CaChr8	15379006	(CT)6	CACAAATCAGCAACACCTGG	TCGCAGAGCTTTCTCTCTCTG	60.2	187	CTRMCAMV35S	TCTCTCTCT	CaMV 35S (Inverted GAGA) found in a 60-nucleotide region downstream of the transcription start site of the	Pentatricopeptide repeat

Table S3: Genomic distribution of physically mapped CNMS marker-associated genes on eight chickpea chromosomes

**Chromosomes**	**Size (Mb) of chromosomes (Pseudomolecules)**	**Number (%) of markers mapped**	**Average map density (kb)**
CaChr1	48.36	80 (12)	604.5
CaChr2	36.63	63 (9.5)	581.4
CaChr3	39.99	70 (10.5)	571.3
CaChr4	49.19	116 (17.4)	424.1
CaChr5	48.17	85 (12.8)	566.7
CaChr6	59.46	108 (16.2)	550.6
CaChr7	48.96	107 (16.1)	457.6
CaChr8	16.48	37 (5.6)	445.4
**Total**	**347.24**	**666**	**521.4**

CaChr: *Cicer arietinum* Chromosome

Table S4: CNMS marker-associated chickpea genes predicted to function as master regulatory transcription factors in plants.

**CNMS markers IDs***	**NCBI Probe IDs**	***Arabidopsis* orthologous gene Accession IDs**	**Gene identity**	**Functions**
Ca-CNMS16	28366836	AT1G62360	SHOOT MERISTEMLESS (STM)	Class I knotted-like homeodomain protein that is required for shoot apical meristem (SAM) formation during embryogenesis and for SAM function throughout the lifetime of the plant. Functions by preventing incorporation of cells in the meristem center into differentiating organ primordia.
Ca-CNMS310	28367004	AT1G52740	H2A.Z	Encodes HTA9, a histone H2A protein.
Ca-CNMS392	28367094	AT3G26744	ICE1	Encodes a MYC-like bHLH transcriptional activator that binds specifically to the MYC recognition sequences in the CBF3 promoter. Mutants are defective in cold-regulated gene expression. Cold stress triggers protein degradation of nuclear GFPICE1 protein, and the RING finger protein HOS1 is required. Sumoylation of ICE1 controls CBF3/DREB1A expression and freezing tolerance.
Ca-CNMS407	28367111	AT5G20240	PI (PISTILLATA)	Floral homeotic gene encoding a MADS domain transcription factor. Required for the specification of petal and stamen identities.
Ca-CNMS564	28367285	AT4G36920	AP2	Encodes a floral homeotic gene, a member of the AP2/EREBP (ethylene responsive element binding protein) class of transcription factors and is involved in the specification of floral organ identity, establishment of floral meristem identity, suppression of floral meristem indeterminancy, and development of the ovule and seed coat. AP2 also has a role in controlling seed mass. Dominant negative allele I28, revealed a function in meristem maintenance-mutant meristems are smaller than normal siblings. AP2 appears to act on the WUS-CLV pathway in an AG independent manner.
Ca-CNMS581	28367304	AT1G69120	AP1 (APETALA1)	Floral homeotic gene encoding a MADS domain protein homologous to SRF transcription factors. Specifies floral meristem and sepal identity. Required for the transcriptional activation of AGAMOUS. Interacts with LEAFY.Binds to promoter and regulates the expression of flowering time genes SVP, SOC1 and AGL24.

*Detailed information on CNMS markers are provided in the Supplemental Table S2
